# Rumen microbial community composition varies with diet and host, but a core microbiome is found across a wide geographical range

**DOI:** 10.1038/srep14567

**Published:** 2015-10-09

**Authors:** Gemma Henderson, Faith Cox, Siva Ganesh, Arjan Jonker, Wayne Young, Global Rumen Census Collaborators, Leticia Abecia, Erika Angarita, Paula Aravena, Graciela Nora Arenas, Claudia Ariza, Graeme T. Attwood, Jose Mauricio Avila, Jorge Avila-Stagno, André Bannink, Rolando Barahona, Mariano Batistotti, Mads F. Bertelsen, Aya Brown-Kav, Andres M. Carvajal, Laura Cersosimo, Alexandre Vieira Chaves, John Church, Nicholas Clipson, Mario A. Cobos-Peralta, Adrian L. Cookson, Silvio Cravero, Omar Cristobal Carballo, Katie Crosley, Gustavo Cruz, María Cerón Cucchi, Rodrigo de la Barra, Alexandre B. De Menezes, Edenio Detmann, Kasper Dieho, Jan Dijkstra, William L. S. dos Reis, Mike E. R. Dugan, Seyed Hadi Ebrahimi, Emma Eythórsdóttir, Fabian Nde Fon, Martín Fraga, Francisco Franco, Chris Friedeman, Naoki Fukuma, Dragana Gagić, Isabelle Gangnat, Diego Javier Grilli, Le Luo Guan, Vahideh Heidarian Miri, Emma Hernandez-Sanabria, Alma Ximena Ibarra Gomez, Olubukola A. Isah, Suzanne Ishaq, Elie Jami, Juan Jelincic, Juha Kantanen, William J. Kelly, Seon-Ho Kim, Athol Klieve, Yasuo Kobayashi, Satoshi Koike, Jan Kopecny, Torsten Nygaard Kristensen, Sophie Julie Krizsan, Hannah LaChance, Medora Lachman, William R. Lamberson, Suzanne Lambie, Jan Lassen, Sinead C. Leahy, Sang-Suk Lee, Florian Leiber, Eva Lewis, Bo Lin, Raúl Lira, Peter Lund, Edgar Macipe, Lovelia L. Mamuad, Hilário Cuquetto Mantovani, Gisela Ariana Marcoppido, Cristian Márquez, Cécile Martin, Gonzalo Martinez, Maria Eugenia Martinez, Olga Lucía Mayorga, Tim A. McAllister, Chris McSweeney, Lorena Mestre, Elena Minnee, Makoto Mitsumori, Itzhak Mizrahi, Isabel Molina, Andreas Muenger, Camila Munoz, Bostjan Murovec, John Newbold, Victor Nsereko, Michael O’Donovan, Sunday Okunade, Brendan O’Neill, Sonia Ospina, Diane Ouwerkerk, Diana Parra, Luiz Gustavo Ribeiro Pereira, Cesar Pinares-Patino, Phil B. Pope, Morten Poulsen, Markus Rodehutscord, Tatiana Rodriguez, Kunihiko Saito, Francisco Sales, Catherine Sauer, Kevin Shingfield, Noriaki Shoji, Jiri Simunek, Zorica Stojanović-Radić, Blaz Stres, Xuezhao Sun, Jeffery Swartz, Zhi Liang Tan, Ilma Tapio, Tasia M. Taxis, Nigel Tomkins, Emilio Ungerfeld, Reza Valizadeh, Peter van Adrichem, Jonathan Van Hamme, Woulter Van Hoven, Garry Waghorn, R. John Wallace, Min Wang, Sinéad M. Waters, Kate Keogh, Maren Witzig, Andre-Denis G. Wright, Hidehisa Yamano, Tianhai Yan, David R. Yanez-Ruiz, Carl J. Yeoman, Ricardo Zambrano, Johanna Zeitz, Mi Zhou, Hua Wei Zhou, Cai Xia Zou, Pablo Zunino, Peter H. Janssen

**Affiliations:** 1 AgResearch Limited, Grasslands Research Centre, Palmerston North 4442, New Zealand; 2 Estacion Experimental del Zaidin, Consejo Superior de Investigaciones Cientificas, 18100 Armilla, Granada, Spain; 3 Animal Nutrition, Biotechnology and Bioindustry Center, Corporación Colombiana de Investigaciones Agropecuarias, Bogota, Colombia; 4 Facultad de Ciencias Veterinarias, Universidad de Concepción, Chillan, Chile; 5 Área Microbiología, Departamento de Patología, Facultad de Ciencias Médicas, Universidad Nacional de Cuyo, Centro Universitario, Parque General San Martín S/N, 5500 Mendoza, Argentina; 6 University of Cundinamarca, Cundinamarca, Colombia; 7 Animal Nutrition, Wageningen UR Livestock Research, 6700 AH Wageningen, The Netherlands; 8 Departamento de Producción Animal, National University of Colombia, AA 1779, Medellín, Colombia; 9 Copenhagen Zoo, DK 2000, Frederiksberg, Denmark; 10 Instituto de Investigaciones Agropecuarias, INIA Remehue, Osorno, Región de Los Lagos, Chile; 11 Department of Animal Science, University of Vermont, Burlington, Vermont 05405, USA; 12 Faculty of Veterinary Science, The University of Sydney, New South Wales 2006, Australia; 13 Department of Biological Sciences, Thompson Rivers University, Kamloops, B.C., V2C 0C8, Canada; 14 Microbiology, School of Biology and Environmental Science, Science Centre West, University College Dublin, Belfield, Dublin 4, Ireland; 15 Colegio de Postgraduados, Institución de Ensenanza e Investigación en Ciensias Agrícolas, CP 56230, Montecillo, Mexico; 16 Instituto de Biotecnología, Instituto Nacional de Tecnología Agropecuaria, Los Reseros y Repetto, 1686 Hurlingham, Argentina; 17 Campo Experimental La Posta, Instituto Nacional de Investigaciones Forestales, Agricolas y Pecuarias, C.P. 94277, Col. Paso del Toro Municipio de Medellín de Bravo, Veracruz, Ver., México; 18 Rowett Institute of Nutrition and Health, University of Aberdeen, Bucksburn, Aberdeen, AB21 9SB, Scotland; 19 Cargill Animal Nutrition Innovation Center, Elk River, Minnesota 55330, USA; 20 Instituto de Patobiología, Instituto Nacional de Tecnología Agropecuaria, Los Reseros y Repetto, 1686 Hurlingham, Argentina; 21 Departamento de Microbiologia, Universidade Federal de Viçosa, Campus UFV, 36570-000 Viçosa, Minas Gerais, Brazil; 22 Animal Nutrition Group, Wageningen University, 6700 AH Wageningen, The Netherlands; 23 Agriculture & Agri-Food Canada, Lacombe, Alberta T4L 1W1, Canada; 24 Department of Animal Sciences, Faculty of Agriculture, Ferdowsi University of Mashhad, Mashhad, Iran; 25 Faculty of Land and Animal Resources, Agricultural University of Iceland, Keldnaholt, IS-112 Reykjavik, Iceland; 26 Department of Agriculture, University of Zululand, KwaDlangezwa, Empangeni, 3886, South Africa; 27 Departamento de Microbiología, Instituto de Investigaciones Biológicas Clemente Estable, Av. Italia 3318, CP 11600, Montevideo, Uruguay; 28 IVITA Marangani, Universidad Nacional Mayor de San Marcos, Lima, Peru; 29 Ministry for Primary Industries Verification Services Hawkes Bay, Silver Fern Farms—Pacific, Whakatu, Hastings, New Zealand; 30 Laboratory of Animal Nutrition, Research Faculty of Agriculture, Hokkaido University, N9W9, Kita-ku, Sapporo 060–8589, Japan; 31 Institute of Agricultural Sciences, Animal Nutrition, ETH Zürich, CH-8092 Zürich, Switzerland; 32 Agricultural, Food & Nutritional Science, University of Alberta, Edmonton, Alberta, T6G 2P5, Canada; 33 Quality Control Department, Mashhad Feed Mill, Mashhad, Iran; 34 Department of Animal Nutrition, Federal University of Agriculture, Abeokuta (FUNAAB), Nigeria; 35 Department of Ruminant Sciences, Agricultural Research Center (ARO), Volcani Institute, Bet Dagan 50250, Israel; 36 Investigación en Ciencias Animales, Instituto de Investigaciones Agropecuarias Kampenaike, Angamos 1056, Punta Arenas, Región de Magallanes, Chile; 37 Natural Resources Institute Finland, FI-31600 Jokioinen, Finland; 38 Department of Biology, University of Eastern Finland, FI-70211 Kuopio, Finland; 39 Department of Animal Science & Technology, Sunchon National University, Suncheon, Jeonnam 540–743, Korea; 40 Animal Studies Building, Gatton Campus and Ecosciences Precinct, The University of Queensland, Dutton Park, Queensland, Australia; 41 Institute of Animal Physiology and Genetics, Laboratory of Anaerobic Microbiology, Academy of Sciences of the Czech Republic, Videnska 1083, 142 20, Prague 4, Czech Republic; 42 Nordic Genetic Resource Center, NO-1431 As, Norway; 43 Swedish University of Agricultural Sciences, Department of Agricultural Research for Northern Sweden, SE-901 83 Umea, Sweden; 44 Department of Animal and Range Sciences, Montana State University, Bozeman, Montana, 59717, USA; 45 Animal Science Genetics, University of Missouri-Columbia, Columbia, Missouri 65211, USA; 46 Department of Molecular Biology and Genetics, Aarhus University, DK-8830 Tjele, Denmark; 47 Buffalo Research Institute, The Chinese Academy of Agricultural Science, Nanning, Guangxi, China; 48 Department of Animal Science, Aarhus University, DK-8830 Tjele, Denmark; 49 Institut National de la Recherche Agronomique, UMR1213 Herbivores, F-63122 Saint-Genes-Champanelle, France; 50 Clermont Université, VetAgro Sup, UMR1213 Herbivores, F-63000 Clermont-Ferrand, France; 51 Université de Lyon, VetAgro Sup, UMR1213 Herbivores, F-69280 Marcy l’Etoile, France; 52 Lethbridge Research Centre, Agriculture & Agri-Food Canada, Lethbridge, Alberta, Canada T1J 4B1; 53 CSIRO Agriculture, Queensland Bioscience Precinct, St. Lucia, Queensland 4067, Australia; 54 DairyNZ, Cnr Ruakura and Morrinsville Roads, Newstead, Hamilton 3240, New Zealand; 55 NARO Institute of Livestock and Grassland Science, 2 Ikenodai, Tsukuba, Ibaraki, 305-0901 Japan; 56 Agroscope, Institute for Livestock Sciences ILS, CH-1725 Posieux, Switzerland; 57 University of Ljubljana, Biotechnical Faculty, Department of Animal Science, SI-1230 Domzale, Slovenia; 58 Cargill Animal Nutrition, Cargill Innovation Center Velddriel, 5334 LD Velddriel, The Netherlands; 59 Research and Innovation Center, Diamond V, Cedar Rapids, Iowa 52404-5260, USA; 60 Forestry Research Institute of Nigeria, Jericho, Ibadan, Oyo State, Nigeria; 61 Agri-Science Queensland, Department of Agriculture and Fisheries (DAF), Ecosciences Precinct Dutton Park, Queensland 4012, Australia; 62 Embrapa Gado de Leite, Rua Eugenio do Nascimento, EP 36038-330 Juiz de Fora (MG), Brazil; 63 Department of Chemistry, Biotechnology and Food Science, Norwegian University of Life Sciences, Aas, Norway; 64 University of Hohenheim, Institute of Animal Science, Animal Nutrition Group, 70599 Stuttgart, Germany; 65 Department of Technology, National Livestock Breeding Center 1, Shibahara, Mafune, Nishigou, Nishishirakawa, Fukushima 961-8511, Japan; 66 Natural Resources Institute Finland, FI-31600 Jokioinen, Finland; 67 Yamagata Prefectural Animal Industrial Institute, Agricultural Research Center, 1076 Torigoe, Shinjo, Yamagata 996-0041, Japan; 68 Department of Biology and Ecology, Faculty of Science and Mathematics, 18000 Niš, Serbia; 69 Institute of Subtropical Agriculture, The Chinese Academy of Sciences, Changsha, China; 70 CSIRO Animal Food and Health Sciences, James Cook University, Townsville, Queensland, Australia; 71 Instituto de Investigaciones Agropecuarias INIA Carillanca, Temuco, Chile; 72 Animal and Bioscience Research Department, Animal & Grassland Research and Innovation Centre, Teagasc, Grange, Dunsany, Co. Meath, Ireland; 73 Agri-Food and Biosciences Institute, Hillsborough, County Down BT26 6DR, Northern Ireland

## Abstract

Ruminant livestock are important sources of human food and global greenhouse gas emissions. Feed degradation and methane formation by ruminants rely on metabolic interactions between rumen microbes and affect ruminant productivity. Rumen and camelid foregut microbial community composition was determined in 742 samples from 32 animal species and 35 countries, to estimate if this was influenced by diet, host species, or geography. Similar bacteria and archaea dominated in nearly all samples, while protozoal communities were more variable. The dominant bacteria are poorly characterised, but the methanogenic archaea are better known and highly conserved across the world. This universality and limited diversity could make it possible to mitigate methane emissions by developing strategies that target the few dominant methanogens. Differences in microbial community compositions were predominantly attributable to diet, with the host being less influential. There were few strong co-occurrence patterns between microbes, suggesting that major metabolic interactions are non-selective rather than specific.

Ruminants are one of the most successful groups of herbivorous mammals on the planet, with around 200 species represented by approximately 75 million wild and 3.5 billion domesticated individuals worldwide^1^. Ruminants are defined by their mode of plant digestion, and have evolved a forestomach, the rumen, that allows partial microbial digestion of feed before it enters the true stomach. Ruminants themselves do not produce the enzymes needed to degrade most complex plant polysaccharides, and the rumen provides an environment for a rich and dense consortium of anaerobic microbes that fulfil this metabolic role. These rumen microbes ferment feed to form volatile fatty acids that are major nutrient sources for the host animal and contribute significantly to ruminant productivity. The host also uses microbial biomass and some unfermented feed components once these exit the rumen to the remainder of the digestive tract. Ruminants have evolved various rumen anatomies and behaviours to thrive on a range of plant species, and this flexibility has enabled them to occupy many different habitats spanning a wide range of climates^2^. These were also important factors in their domestication, allowing conversion of human-indigestible plant material into readily-accessible animal goods, especially dairy products, meat, and useful fibres. Ruminants have thus played a vital role in sustaining and developing many human cultures, as well as being used as draft animals and having religious and status values.

Rumen microbes can be assigned to different functional groups, such as cellulolytics, amylolytics, proteolytics, etc., which degrade the wide variety of feed components or further metabolize some of the products formed by other microbes^3^. For example, methanogens, the methane-forming archaea, are among those that metabolize hydrogen formed by some fermentative microbes to form methane. The methane generated during this fermentation contributes to global anthropogenic greenhouse gas emissions^4^ and represents a 2–12% loss of feed energy for the animal^5^. Differences in rumen microbial communities underlie variations in methane formation^6^ and the conversion of feed to animal products^7^,^8^. Therefore, understanding these communities is key to understanding ruminal transformations of plant material to both undesirable and useful ruminant products.

The aim of this study was to determine the composition of the microbiota in rumen and foregut samples from 742 individual animals from around the world. The resulting dataset allowed us to determine that dietary factors dominate over host species in determining microbial community composition, identify the dominant microbes and their potential associations, and describe the degree of similarity of rumen microbial communities worldwide.

## Results and Discussion

This is the largest single study to examine microbial communities across a range of ruminant and camelid species, diets, and geographical regions. A standardised pipeline was used to process samples in order to minimise variation introduced by processing steps such as DNA extraction or PCR amplification. This is important for detecting authentic patterns rather than ones introduced by methodological differences between different studies^9^. The primers chosen amplify, to the best of our current knowledge, the target gene regions from nearly all known bacteria, archaea, and rumen ciliates.

### Dominant rumen microbes

**Figure 1:**
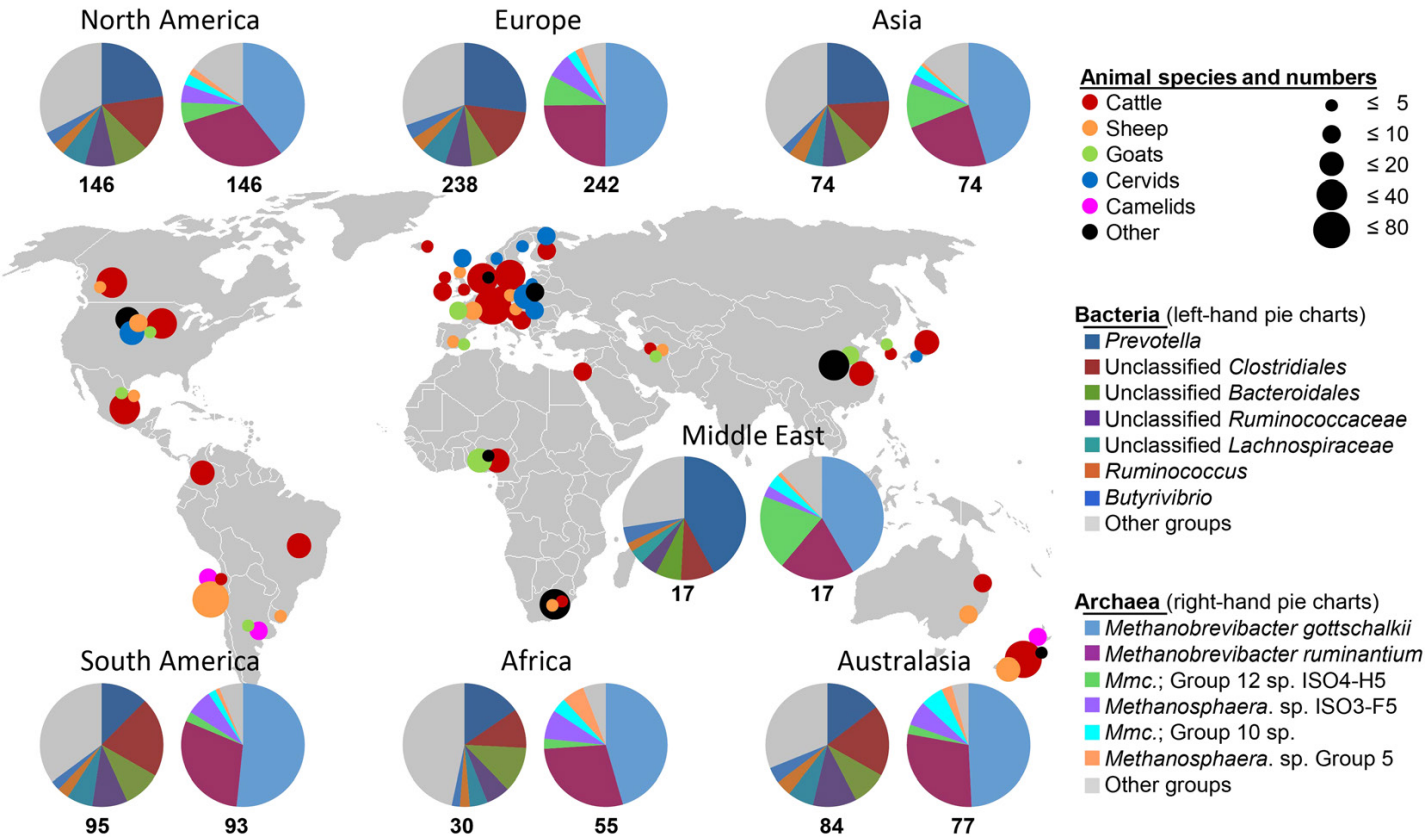
Origins of samples and their bacterial and archaeal community compositions in different regions. Numbers below pie charts represent the number of samples for which data were obtained. The most abundant bacteria and archaea are named in clockwise order starting at the top of the pie chart. Further details of samples and community composition are given in Supplementary Tables 1, 2, 3, and 4 and Supplementary Data 1. *Mmc*. *Methanomassiliicoccales.* The map was sourced from Wikimedia Commons (http://commons.wikimedia.org/wiki/File:BlankMap-World-v2.png, original uploader Roke, accessed May 2013). Pie charts were produced in Microsoft Excel and the composite image generated with Microsoft PowerPoint and Adobe Illustrator. https://creativecommons.org/licenses/by-sa/3.0/deed.en

Despite the range of ruminants with different feeding strategies and diets, similar rumen bacteria were abundant around the world (Fig. 1). There was some variation in bacterial community compositions in animals from different regions, likely to be caused by differences in diet, climate, and farming practices. The 30 most abundant bacterial groups (Greengenes^10^ taxonomy summarised at the genus-level) were all found in over 90% of samples, and together comprised 89.4% of all sequence data (Supplementary Table 1) and were similar to those described in an earlier meta-analysis of rumen microbial communities^11^. All 30 are known rumen-inhabiting bacteria. Because the samples came from a wide range of ruminant species, diets, and geographical locations, these data suggest that new dominant bacteria are not likely to be found in future studies. The seven most abundant bacterial groups comprised 67.1% of all bacterial sequence data, were detected in all samples (Supplementary Fig. 1), and can be considered the “dominant” rumen bacteria. They were *Prevotella*, *Butyrivibrio*, and *Ruminococcus*, as well as unclassified *Lachnospiraceae*, *Ruminococcaceae*, *Bacteroidales*, and *Clostridiales*. These might be considered a “core bacterial microbiome” at the genus level or higher, because they are present in a large selection of ruminants, so confirming the suggestion that there is a core rumen microbiome^9^. However, these bacterial groups were not equally abundant in all animal species (*P* ≤ 0.005; Supplementary Table 2). With the exception of *Butyrivibrio*^12^, these groups are not adequately represented by characterised cultures^13^, and their functions are not well understood.

**Figure 2:**
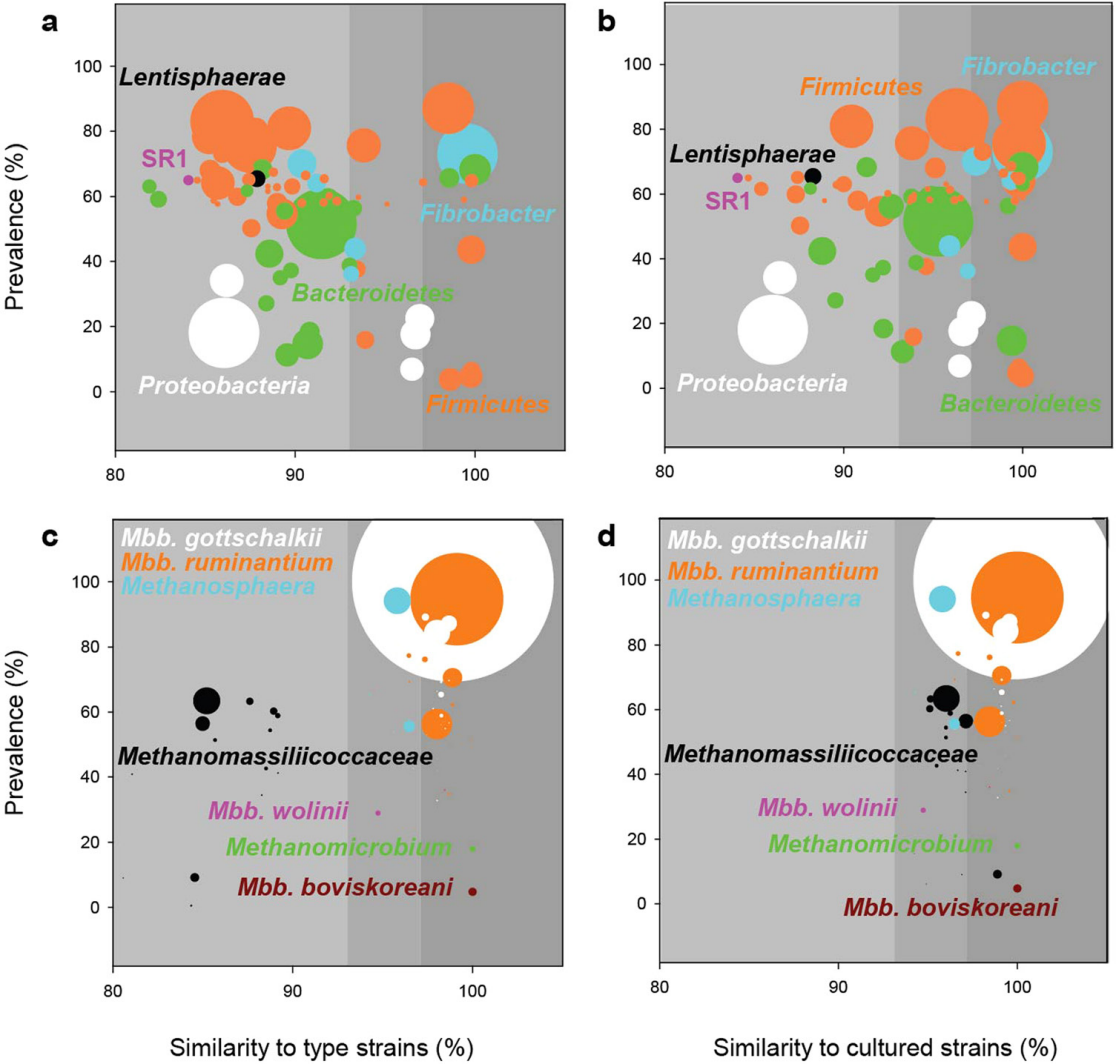
Dominant bacterial and archaeal operational taxonomic units (OTUs). Similarities (Supplementary Tables 8 and 9) of the 50 most abundant and 50 most prevalent bacterial (77 unique OTUs, (**a,b**) and archaeal (64 unique OTUs, **c,d**) OTUs to the most closely related type (**a,c**) and cultured (**b,d**) strains are plotted together with prevalence and abundance data. Background shading indicates nominal within-species (dark grey), within-genus (mid grey) and below genus (light grey) similarities. Prevalence indicates the percentage of samples that an OTU occurs in. The size of each circle indicates the mean abundance of each OTU (Supplementary Tables 8 and 9). Bacterial OTU abundances were multiplied by a factor of 15 relative to archaeal OTUs. *Mbb*. *Methanobrevibacter.*

Inspection of the most abundant and prevalent bacterial operational taxonomic units (OTUs) in the dataset showed that only 14% fell within a named species, and 70% were not even within a formally recognised genus (Fig. 2a). When cultured isolates from as-yet unnamed species were included in the analysis, the dominant OTUs were better (35%) but still poorly represented by cultures that belonged to potentially the same species (Fig. 2b). This study shows that, while we appear to recognize the dominant rumen bacteria, considerable microbiological effort is still required to understand them. Some efforts have been made to isolate more cultures and gather more information about these bacteria^13^,^14^. For example, the genomes of *Prevotella* aff. *ruminicola* Tc2-24, rumen bacterium R-7, and other isolates whose 16S rRNA gene sequences are similar to those of dominant rumen bacterial OTUs (Fig. 2b), have been sequenced as part of the Hungate1000 project^15^.

Because there is a flux of both liquids and solids through the rumen^16^, microbes must actively metabolize to gain energy and multiply to counteract washout and so maintain populations in the rumen^17^. The dominant bacteria found in this study are therefore likely to be responsible for the majority of the transformation of ingested feed in the rumen and camelid foregut, especially of cellulose, hemicellulose, pectin, starch, fructan, organic acids, and protein, as these are the major energy-yielding substrates used for microbial growth^17^. There is also a convergence of bacterial community structure in the rumen and in the crop of the hoatzin, a bird that relies on a foregut fermentation of ingested leaves^18^. Thus microbial community structure seems to be driven by the similarity of organ function extending across the rumen, the camelid foregut, and the crop of this unusual bird. More efforts should go into characterizing the metabolism and roles of these bacteria that are the responsible for the majority of feed fermentation, with the aim of enhancing animal productivity and reducing methane emissions.

Nearly all the archaea were identified as methanogens known to be residents of the rumen (Supplementary Table 3, Supplementary Text 1), and their relative abundances were comparable to previous studies^19^. The dominant archaeal groups were remarkably similar in all regions of the world (Fig. 1). This universality and limited diversity was also recently noted in survey of archaea in New Zealand ruminants^20^ and could make it possible to successfully mitigate methane emissions by developing strategies, such as vaccines or small-molecule inhibitors, that target the few dominant methanogens.

Members of the *Methanobrevibacter gottschalkii* and *Methanobrevibacter ruminantium* clades were found in almost all samples, and were the two largest groups, accounting for 74% of all archaea. Together with a *Methanosphaera* sp. and two *Methanomassiliicoccaceae*-affiliated groups, the five dominant methanogen groups comprised 89.2% of the archaeal communities (Supplementary Fig. 1), showing that rumen archaea are much less diverse than rumen bacteria. This likely reflects the narrow range of substrates they use. *Methanomicrobium* has previously been reported as abundant in ruminants in Asia^19^. In our study, they were found to comprise >5% of the archaeal community of some Australian, Brazilian, Chinese, North American, and South African cattle, as well as South African sheep, showing them to be widely distributed, but not universally prevalent. The five dominant methanogen groups were not equally abundant in all animal species groups (*P* ≤ 0.005; Supplemental Table 4). In contrast to bacteria, the rumen archaea are better represented by cultures, with 58% of the most abundant and prevalent OTUs falling within a named species, and all but 22% within named genera (Fig. 2c). All of the latter were members of *Methanomassiliicoccales*, which is an order of relatively poorly-characterised methanogens^21^ for which representative cultures of as-yet unnamed species and genera are available (Fig. 2d)^22^. The 50 most abundant OTUs accounted for 74.5% of the archaeal sequence data, again indicating a much lower diversity than in the bacteria, where the 50 most abundant OTUs made up only 11.0%.

By assigning physiologies (Supplementary Table 5) to the sequence abundance information (Supplementary Table 3), it can be concluded that 77.7% of archaea were hydrogenotrophic methanogens, while 22.1% had the ability to grow with hydrogen plus methyl groups derived from methanol or methylamines. Methanogens able to form methane from acetate (*Methanosarcina* spp. and *Methanosaeta* spp.) were extremely rare (<0.015%; Supplemental Data 1), as expected based on their general slow growth rates that would not allow them to be maintained in the rumen under normal conditions.

Almost all protozoal sequence data (>99.9%) were assigned to 12 genus-equivalent protozoal groups (Supplementary Table 6). It was apparent that the variability of protozoa between and within cohorts of co-located animals was much greater than that of bacteria and archaea (Supplementary Fig. 2). It has been reported that there is strong host individuality of rumen protozoal community structure^9^, and this is evident in our study. The genera *Entodinium* and *Epidinium* dominated, occurring in more than 90% of samples and representing 54.7% of protozoal sequence data (Supplementary Fig. 1). Many of the protozoal genera were present in greater than 70% of the samples, indicating a wide prevalence. Genera such as *Enoploplastron* and *Ophryoscolex* had a wider than expected host distribution. They are considered to be mainly present in sheep and cattle, respectively^23^, but we also found *Enoploplastron* in cattle, deer, and reindeer samples from twelve countries, and *Ophryoscolex* in buffalo, goats, deer, sheep, and giraffe samples from 18 countries. Although different rumen protozoa are reported to have limited host and geographical distributions, host specificity has been questioned^24^. It seems likely that further investigation will demonstrate greater ubiquity of the rumen protozoa.

### Effects of diet and host on microbial community composition

Because the abundance of microbial groups varied between animal species groups and cohorts (Supplementary Tables 2 and 4; Supplementary Fig. 2), we looked for factors that might underlie this. Rumen and camelid foregut microbial community structure could be expected to be shaped by morphological, physiological, and even behavioural characteristics that evolved along with the varied feeding strategies in the various ruminant lineages^2^. Indeed, adaptation has resulted in a diversity of rumen sizes and passage rates of rumen contents, allowing ruminant species to exploit a range of feed types. In addition to feed composition effects^25^, these host adaptations might also play a role in regulating rumen microbial community structure. Because our dataset was from ruminants and camelids from different lineages consuming a range of diets, host and diet effects on rumen microbial community structure could be separated.

**Figure 3:**
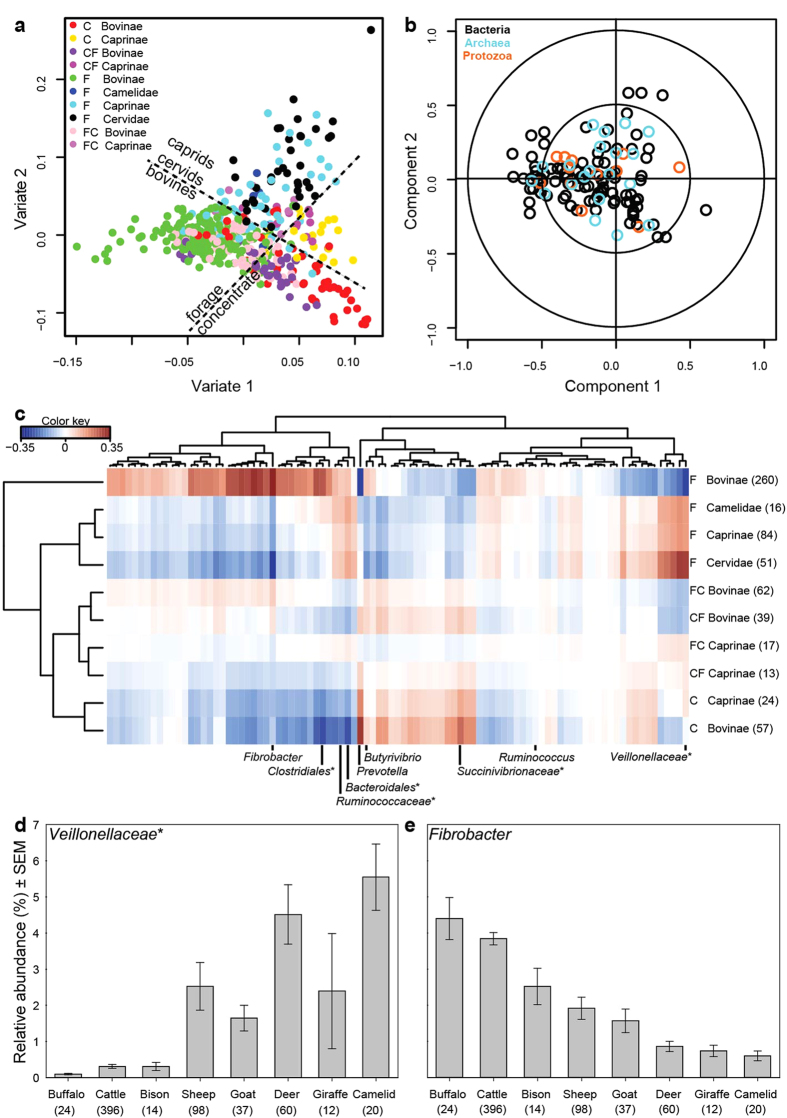
Effect of host species and dietary forage to concentrate ratios on microbial communities. Diets were grouped (Supplementary Table 7) as forage-dominated (F), mixed forage-concentrate (50–70% forage, FC), mixed concentrate-forage (50–70% concentrate, CF), or concentrate-dominated (C). (**a**) Discriminant analysis of microbial communities in samples (represented by points coloured by animal and diet) revealed that both host and diet determined community composition. (**b**) Bi-plot that shows microbial groups (identified by colours) underlying the separation of samples in panel (**a**). Several bacterial groups strongly discriminate the samples by host and diet, indicated by their presence towards the outside of the bi-plot. Archaeal and protozoal groups are less discriminatory, and so are clustered nearer the centre. (**c**) The heatmap shows that bacterial abundances are differentially associated with diet and host (colour key shows the association score; see Supplementary Figs 3–5 for additional data). (**d**) Unclassified *Veillonellaceae*, and (**e**) *Fibrobacter* are examples of bacteria that caused bovines and caprids to cluster separately from other species in the heat map. The number of samples in each category is given in parentheses in panels (**c–e**). *indicates unclassified bacteria within an order or family.

To look at diet and host effects, we classified the diets based on forage and browse or concentrate content (Supplementary Table 7) and grouped the animals according to their lineage (Supplementary Data 1). Microbial communities could clearly be discriminated by both host and diet (Fig. 3a), with bacteria being the main drivers behind the observed differences (Fig. 3b). This probably reflects their more diverse metabolic capabilities compared with the less versatile archaea and protozoa. We investigated the patterns of microbial abundances across hosts and diets (Fig. 3c, Supplementary Fig. 3–6). *Ruminococcus*, one of the dominant bacteria, was relatively evenly distributed, but this was an exception. For many bacteria, diet was the major factor determining relative abundance. Bacterial communities from forage-fed animals were similar to each other, those from concentrate-fed animals were similar to each other, but distinct from those in forage-fed animals, and those from animals fed mixed diets were intermediate between these. Unclassified *Bacteroidales* and *Ruminococcaceae* were more abundant in all animals fed forages. Some as-yet poorly characterised *Bacteroidales* are postulated to be able to degrade cellulose, and their genomes encode a broad range of plant polysaccharide degrading capabilities^26^,^27^, which could explain their pattern of distribution. In contrast, members of *Prevotella* and unclassified *Succinivibrionaceae* were more abundant in animals fed diets containing concentrate. Based on the physiologies of cultured relatives^28^,^29^, these are probably major producers of propionate and the propionate-precursor succinate, and so are responsible for the greater levels of propionate formed from concentrate-rich diets^25^. The abundance of only a few other major bacterial groups was associated with host lineage (Fig. 3c). For example, unclassified *Veillonellaceae* were proportionally more abundant in sheep, deer, and camelids (Fig. 3d). This may be related to differences in rumen and camelid foregut sizes, anatomy, and feeding frequencies compared to bovines^2^.

The relative abundances of several major bacterial groups were affected by both host and diet (Fig. 3c). Unclassified *Clostridiales* were most abundant in bovines fed forage and least abundant in bovines fed high concentrate diets, while in caprids, cervids, and camelids these diet differences were far less pronounced. *Butyrivibrio* was most abundant in rumen samples from bovines fed mixes of forage and concentrates. *Fibrobacter* was most abundant in bovines fed forage. When concentrate was included in bovine diets, the relative abundance of *Fibrobacter* was decreased, but it was still more abundant than in other animals. To examine its distribution in more detail, we compared *Fibrobacter* abundances across different ruminant species and found significantly higher levels in bovines compared to deer, sheep, or camelids (Fig. 3e). These data suggest that *Fibrobacter* is favoured in the bovine rumen and, given that it is cellulose degrader^30^, may play an essential role in the degradation of plant fibre in cattle.

Overall, diet was a major determinant of bacterial community structure. This may be because physical and chemical characteristics of the feed determine the different microbial niches available. In contrast to the post-gastric mammalian digestive tract^31^, and due to the sheer volume of digesta and feed input, there is probably less shaping of the rumen microbial community by local host biological factors such as the immune system, secreted antimicrobial peptides, host-cell glycosylation, and host-derived nutrients.

### Associations between rumen microbes

**Figure 4:**
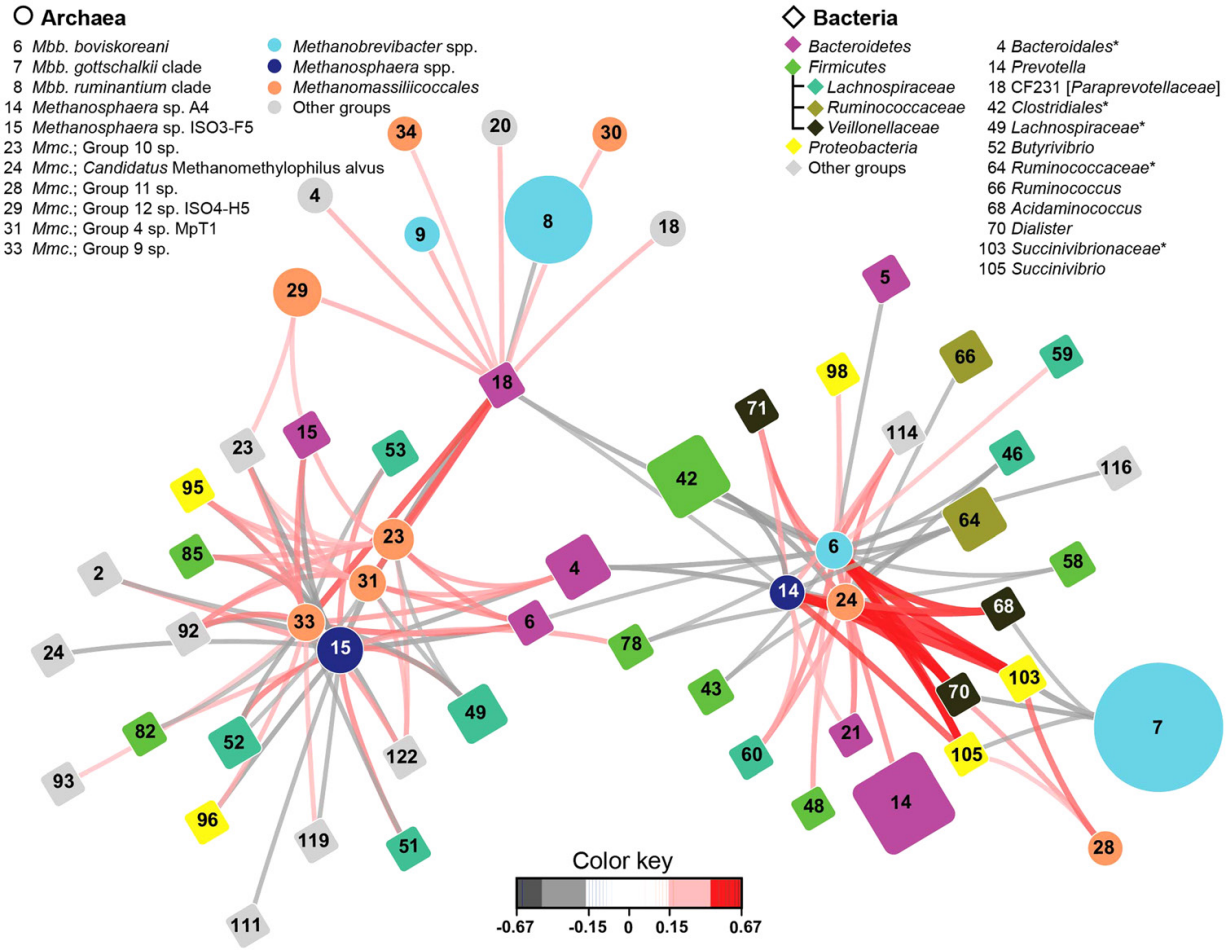
Associations between bacteria and archaea. The network is based on association scores computed via regularised canonical correlation analysis with an absolute association score greater than 0.15. The colour of the lines indicates the strength of the association. The sizes of the diamonds and circles indicate the mean average abundance and microbial groups are identified by numbers (Supplementary Tables 1 and 3). *Mbb*. *Methanobrevibacter*, *Mmc*. *Methanomassiliicoccales*, *indicates unclassified bacteria within a family.

The abundance patterns within bacterial, archaeal, and protozoal communities in different hosts fed different diets showed that certain microbes exhibited parallel patterns of relative abundance (Fig. 3c, Supplementary Figs 2–6). We therefore looked for correlations *within* and *between* bacteria, archaea, and protozoa (Fig. 4 and Supplementary Fig. 7), reasoning that specific associations should be seen across diets, hosts, and geography. Negative correlations of abundances of groups were observed *within* the bacteria, archaea, and protozoa, including replacement effects between dominant groups within each of these (Supplementary Text 1 and Supplementary Fig. 7). Few strong positive correlations were found within bacteria, archaea, and protozoa. For example, there was a strong correlation between *Veillonellaceae* and the TG5 group, driven by their co-occurrence within cervids and caprids. These microbes may cooperate in the rumen, or they may share similar requirements and so certain hosts and diets would offer better opportunities for their growth. This explanation could also underlie the strong positive correlations observed between different groups of methylotrophic methanogens (Supplementary Fig. 7). They may be responding to diets rich in methyl groups, such as feeds with high levels of pectins or osmolytes such as betaine. The strongest correlation within protozoa was a positive one between *Dasytricha* and *Isotricha*. These two genera of holotrichous protozoa display very similar spectra of substrate use, including use of plant soluble sugars and storage carbohydrates^24^, again suggesting that co-occurrence may be due to exploitation of similar opportunities.

We also investigated associations *between* bacteria, archaea, and protozoa. Strikingly, no strong correlations were detected between archaea and protozoa (Supplementary Fig. 7). Methanogens are known to colonize protozoa, and this mutualistic relationship is believed to enhance methane formation in the rumen^32^. The occurrence of specific symbioses between methanogens and rumen protozoa has been speculated on, but not convincingly demonstrated^33^. The lack of strong co-occurrence patterns within this study indicates that these undoubtedly important associations are probably non-specific, or occur at a strain level. Further investigation is required to corroborate this interesting finding, as mechanisms that mediate the colonization of protozoa by archaea remain to be elucidated. These could have interesting evolutionary aspects if they allow non-specific interactions to form or are mediated by strain-specific mechanisms that confer different partner specificities within archaeal or protozoal species. In contrast, there were some positive associations between bacterial and protozoal groups. Most noticeable were the associations of *Isotricha* and *Dasytricha* with *Fibrobacter*. *Fibrobacter* were reported to decrease in abundance in animals where protozoa were eliminated^34^, indicating that there may be a mutually beneficial relationship between these protozoa and *Fibrobacter*, which are surface colonizers of plant material^23^.

No strong associations were found *between* the most abundant bacteria and archaea (Fig. 4). This was surprising, since rumen bacteria degrade feed and produce the substrates that methanogens use for growth, mainly hydrogen and methyl groups. In contrast, there were distinct positive associations between some less abundant bacteria and archaea. The strongest association was between bacteria such as the succinate-producing *Succinivibrionaceae*, the succinate-using *Dialister*, and the amino-acid-fermenting *Acidaminococcus*, and methanogens belonging to the *Methanomassiliicoccaceae*, *Methanosphaera* sp. A4, and *Methanobrevibacter boviskoreani*. *Succinivibrio* spp. degrade pectin^28^, and methanol is required for growth of *Methanomassiliicoccaceae*^35^ and *Methanosphaera*^36^, explaining part of this pattern. Other associations were between the methylotrophic methanogen *Methanosphaera* sp. ISO3-F5 and different bacteria, including members of *Lachnospiraceae*. These associations may be based on the ability of *Lachnospiraceae* to degrade pectin and so provide methanol as a substrate for the methylotrophs^37^. The associations between other *Methanomassiliicoccaceae* groups and various unclassified members of *Bacteroidales* suggest the possibility of yet further methanol-dependent metabolic interactions. In contrast to archaeal-protozoal interactions, these findings suggest that some archaeal-bacterial interactions are specific, inferring specialised mechanisms for partner recognition or very similar requirements for growth. The basis for these associations remains to be determined. However, the general lack of strong association patterns between protozoa and the major bacteria on the one hand, and the major methanogen groups on the other, suggests that conserved mechanisms may mediate the interactions between hydrogen producing and hydrogen consuming microbes, allowing flexible interactions. This may aid methane mitigation research, since interfering with these potentially universal mechanisms could slow the rate of hydrogen transfer and so slow methane formation^38^. It may also be that the interactions mainly occur via pools of common metabolites, especially where the end products of one group form the substrates of another.

The results of this survey showed that the rumen microbial ecosystem is dominated by a core community composed of poorly-characterised microbes, especially amongst the bacteria. Diet had more influence than animal species on rumen or camelid foregut microbial community composition. Rumen ecosystems are typified by strong metabolic interactions between microbes that facilitate the fermentation of plant material to products useful for both the host and other rumen microbes^3^,^17^,^25^,^32^. The relatively few co-occurrence patterns seen in this study suggest that these microbial interactions do not rely on exclusive associations, and could indicate considerable promiscuity between members of interacting functional groups. Analysis at metagenomic and metatranscriptomic levels could in future uncover whether common functional elements that facilitate interactions are shared among multiple species. It seems plausible that functional redundancy among the microbes^9^ means that multiple microbial species can fulfil the same function, with different combinations of microbes being co-selected depending on the diet. This flexibility of rumen microbial community structure would confer on the ruminant host the ability to exploit a variety of different plant feeds.

## Methods

### Geographical distribution and diversity of gastrointestinal tract content samples

A total of 742 samples from 32 species or sub-species of ruminants and other foregut fermenters in 35 countries and seven global regions were selected for sequencing of microbial marker genes (Fig. 1, Supplementary Data 1). The samples were from cattle, bison, and buffalo (bovines), sheep and goats (caprids), deer (cervids), and alpacas, llamas, and guanacos (camelids), including diverse breeds of domestic cattle, sheep, and goats, and were largely made up of small cohorts of four or more co-located individuals consuming the same diet. We included foregut samples of camelids in this study, recognizing that these organs have a common function but evolved separately^39^. The use of animals, including welfare, husbandry, experimental procedures, and the collection of samples used for this study, was, where applicable, approved by named institutional and/or licensing committees and performed in accordance with approved institutional and regulatory guidelines (please refer to Supplementary Data 1 for details of these).

### Sample collection, DNA extraction, amplification and processing of samples for high-throughput sequencing

To minimise variation introduced by differing methodologies, such as choice of sampling or DNA extraction method^40^ and primer-driven gene amplification biases^41^, we used a standardised pipeline to process samples (unless indicated otherwise in Supplementary Data 1). Briefly, approximately 20 g of whole (i.e., solid and liquid) mid-rumen or camelid foregut contents were collected via stomach tube, cannula, or post mortem as previously described^35^. Samples were immediately frozen, freeze-dried, and then couriered to AgResearch. Freeze-dried samples were homogenised in a coffee blender and DNA was extracted from a representative 30 mg subsample using the PCQI method^40^,^42^. We assessed the structure of microbial communities by sequencing regions of bacterial and archaeal 16S rRNA genes and ciliate protozoal 18S rRNA genes in triplicate as described previously^35^,^37^ using primers comprised of (5′ to 3′) a sequencing adapter (A or B), a sample-unique 12-base error-correcting Golay barcode on one of each primer pair, a two-base linker, and a group-specific sequence targeting the marker gene. For bacteria, the primers were Ba515Rmod1 (adapter A-barcode-GT-CCGCGGCKGCTGGCAC) and Ba9F (adapter B-AC-GAGTTTGATCMTGGCTCAG). For archaea, the primers were Ar915aF (adapter A-barcode-GT-AGGAATTGGCGGGGGAGCAC) and Ar1386R (adapter B-CA- GCGGTGTGTGCAAGGAGC). For protozoa, the primers were Reg1320R (adapter A-barcode-TC-AATTGCAAAGATCTATCCC) and RP841F (adapter B-AA-GACTAGGGATTGGARTGG). Linker A was CCATCTCATCCCTGCGTGTCTCCGACTCAG and linker B was CCTATCCCCTGTGTGCCTTGGCAGTCTCAG. Amplicons were sequenced using 454 GS FLX Titanium chemistry at Eurofins MWG Operon (Ebersberg, Germany). Sample processing and pipeline reproducibility controls were performed to identify variation introduced during sample processing (Supplementary Text 1). Sequence data are available from GenBank [accession numbers PRJNA272135, PRJNA272136, and PRJNA273417].

### Phylogenetic analysis of sequencing data

Pyrosequence data were processed and analysed using the QIIME software package version 1.8^43^. Sequences over 400 bp in length with an average quality score over 25 were assigned to a specific sample via the barcodes. The number of bacterial, archaeal, and ciliate protozoal sequencing reads available for analysis are summarised in Supplementary Data 1. Sequence data were grouped into operational taxonomic units (OTUs) sharing over 97% (bacteria – UCLUST^44^), 99% (archaea - UCLUST) or 100% (ciliate protozoa – prefix_suffix option in QIIME) sequence similarity. Sequences were assigned to phylogenetic groups by BLAST^45^. Bacterial 16S rRNA genes were assigned using the Greengenes database version 13_5^10^, archaeal 16S rRNA genes using RIM-DB version 13_11_13^22^ and ciliate protozoal 18S rRNA genes against an in-house database^46^. Bacterial and ciliate protozoal data were summarised at the genus level. Archaea were summarised at the species level. Samples for which low read numbers were obtained or that contained high proportions of sequences from “exogenous” bacteria (i.e., likely environmental contaminants such as *Stenotrophomonas*) were excluded from further analyses (Supplementary Text 1).

The identity of the most abundant and prevalent OTUs was determined using BLAST^45^ against sequences from type material and against all sequences (excluding sequences from model organisms or environmental samples) in the nt database^47^. Bellerophon (version 3, 200 bp window, Huber-Hugenholtz correction^48^) was used to identify chimeric OTU sequences. Sequence similarities greater than 97% and 93% were used as cut-offs to classify OTUs at species- and genus level, respectively. The rationale for these cut-offs was discussed by Kenters *et al.*^49^.

### Simplified classification of dietary information and other factors

The range of diets consumed by the animals from which the samples came was highly diverse and complex. For this reason, and where the information was available, diets were categorised in terms of forage type, forage plant, and forage to concentrate ratio (Supplementary Table 7). Diets likely to contain >5% starch (e.g., whole or grain crops of maize, barley, wheat, rice, as well as pea, potato, sorghum, etc.) or >5% pectin (e.g., beets or legumes such as alfalfa and clover) were also identified. Animals that had been fed their respective diets for less than a two-week period were noted in Supplementary Data 1. Factors such as gender, age, modifications (e.g., cannulation), treatments (e.g., antibiotics, drench, surgery), farming conditions, season, contact with other animals, and sample processing steps that may affect apparent microbial community compositions (e.g., DNA extraction method, sample fraction used, sample storage, etc.) were also recorded (Supplementary Data 1). Where details were not provided, latitude, longitude, and elevation were estimated using http://www.mapcoordinates.net/en. Climate zones were designated according to the Köppen-Geiger climate classification scheme^50^.

### Statistical analyses

The resulting dataset allowed us to establish whether animal or dietary factors relate to rumen and camelid foregut microbial community composition, identify the dominant microbes and their potential associations, and describe the degree of similarity of rumen and camelid foregut microbial communities worldwide. Statistical analyses of microbial data were performed using GenStat for Windows^51^, R software^52^, and QIIME^43^. Principal coordinate analysis of Bray-Curtis dissimilarity matrices, analysis of variance, sparse partial least squares discriminant analysis (sPLS-DA, using a sPLS regression approach), and canonical discriminant analyses (CDA) of microbial community composition data in context of the metadata (Supplementary Data 1) were used to identify impacts of factors such as host lineage, diet, etc. on rumen and camelid foregut microbial communities and to identify the groups associated with these factors. Pearson, Spearman, SparCC^53^, and regularised canonical correlation analyses (CCA) were used to identify associations within and between archaeal, bacterial, and protozoal groups. Association scores were visualised as relevance networks and clustered image maps (CIM, heatmaps) representing the first two dimensions. González *et al.* provides a comprehensive overview of sPLS-DA, CCA and the corresponding ‘pairwise associations’, network and CIM techniques and their application^54^.

## Additional Information

**How to cite this article**: Henderson, G. *et al.* Rumen microbial community composition varies with diet and host, but a core microbiome is found across a wide geographical range. *Sci. Rep.* **5**, 14567; doi: 10.1038/srep14567 (2015).

## Supplementary Material

Supplementary Information

Supplementary Dataset 1

## References

[b1] Hackmann T. J. & Spain J. N. Invited review: ruminant ecology and evolution: perspectives useful to ruminant livestock research and production. J. Dairy Sci. 93, 1320–1334 (2010). 20338409 10.3168/jds.2009-2071

[b2] Hofmann R. R. Evolutionary steps of ecophysiological adaptation and diversification of ruminants: a comparative view of their digestive system. Oecologia 78, 443–457 (1989). 10.1007/BF00378733 28312172

[b3] Hungate R. E. The Rumen and its Microbes. (Academic Press, 1966).

[b4] Ripple W. J. *et al.* Ruminants, climate change and climate policy. Nat. Clim. Change 4, 2–5 (2014).

[b5] Johnson D. E. & Ward G. M. Estimates of animal methane emissions. Environ. Monit. Assess. 42, 113–141 (1996). 24193497 10.1007/BF00394046

[b6] Kittelmann S. *et al.* Two different bacterial community types are linked with the low-methane emission trait in sheep. PLoS ONE 9, e103171 (2014). 25078564 10.1371/journal.pone.0103171 PMC4117531

[b7] Jami E., White B. A. & Mizrahi I. Potential role of the bovine rumen microbiome in modulating milk composition and feed efficiency. PLoS ONE 9, e85423 (2014). 24465556 10.1371/journal.pone.0085423 PMC3899005

[b8] Carberry C. A., Kenny D. A., Han S., McCabe M. S. & Waters S. M. Effect of phenotypic residual feed intake and dietary forage content on the rumen microbial community of beef cattle. Appl. Environ. Microbiol. 78, 4949–4958 (2012). 22562991 10.1128/AEM.07759-11 PMC3416373

[b9] Weimer P. J. Redundancy, resilience, and host specificity of the ruminal microbiota: implications for engineering improved ruminal fermentations. Front. Microbiol. 6, 296 (2015). 25914693 10.3389/fmicb.2015.00296 PMC4392294

[b10] McDonald D. *et al.* An improved Greengenes taxonomy with explicit ranks for ecological and evolutionary analyses of bacteria and archaea. ISME J. 6, 610–618 (2012). 22134646 10.1038/ismej.2011.139 PMC3280142

[b11] Kim M., Morrison M. & Yu Z. Status of the phylogenetic diversity census of ruminal microbiomes. FEMS Microbiol. Ecol. 76, 49–63 (2011). 21223325 10.1111/j.1574-6941.2010.01029.x

[b12] Paillard D. *et al.* Relation between phylogenetic position, lipid metabolism and butyrate production by different *Butyrivibrio*-like bacteria from the rumen. Antonie van Leeuwenhoek 91, 417–422 (2007). 17077990 10.1007/s10482-006-9121-7

[b13] Creevey C. J., Kelly W. J., Henderson G. & Leahy S. C. Determining the culturable accessibility of the rumen bacterial microbiome. Microb. Biotechnol. 7, 467–479 (2014). 24986151 10.1111/1751-7915.12141 PMC4229327

[b14] Nyonyo T., Shinkai T., Tajima A. & Mitsumori M. Effect of media composition, including gelling agents, on isolation of previously uncultured rumen bacteria. Lett. Appl. Microbiol. 56, 63–70 (2013). 23106798 10.1111/lam.12019

[b15] McAllister T. A. *et al.* Ruminant Nutrition Symposium: Use of genomics and transcriptomics to identify strategies to lower ruminal methanogenesis. J. Anim. Sci. 93, 1431–1449 (2015). 26020166 10.2527/jas.2014-8329

[b16] Uden P., Rounsaville T. R., Wiggans G. R. & Van Soest P. J. The measurement of liquid and solid digesta retention in ruminants, equines and rabbits given timothy (*Phleum pratense*) hay. Br. J. Nutr. 48, 329–339 (1982). 6810917 10.1079/bjn19820117

[b17] Van Soest P. J. Nutritional Ecology of the Ruminant. 2 edn, (Cornell University Press, 1994).

[b18] Godoy-Vitorino F. *et al.* Comparative analyses of foregut and hindgut bacterial communities in hoatzins and cows. ISME J. 6, 531–541 (2012). 21938024 10.1038/ismej.2011.131 PMC3280141

[b19] Janssen P. H. & Kirs M. Structure of the archaeal community of the rumen. Appl. Environ. Microbiol. 74, 3619–3625 (2008). 18424540 10.1128/AEM.02812-07 PMC2446570

[b20] Seedorf H., Kittelmann S. & Janssen P. H. Few highly abundant operational taxonomic units dominate within rumen methanogenic archaeal species in New Zealand sheep and cattle. Appl. Environ. Microbiol. 81, 986–995 (2015). 25416771 10.1128/AEM.03018-14 PMC4292475

[b21] Borrel G. *et al.* Comparative genomics highlights the unique biology of *Methanomassiliicoccales*, a *Thermoplasmatales*-related seventh order of methanogenic archaea that encodes pyrrolysine. BMC Genomics 15, 679 (2014). 25124552 10.1186/1471-2164-15-679 PMC4153887

[b22] Seedorf H., Kittelmann S., Henderson G. & Janssen P. H. RIM-DB: a taxonomic framework for community structure analysis of methanogenic archaea from the rumen and other intestinal environments. PeerJ 2, e494 (2014). 25165621 10.7717/peerj.494 PMC4137658

[b23] Williams A. G. & Coleman G. S. The Rumen Protozoa. (Springer-Verlag New York Inc., 1992).

[b24] Dehority B. A. in Parasitic Protozoa Vol. 3 (eds Kreier J. P. & Baker J. R.) Ch. 1, 1–42 (Academic Press, Inc., 1993).

[b25] Russell J. B. & Rychlik J. L. Factors that alter rumen microbial ecology. Science 292, 1119–1122 (2001). 11352069 10.1126/science.1058830

[b26] Pope P. B. *et al.* Metagenomics of the Svalbard reindeer rumen microbiome reveals abundance of polysaccharide utilization loci. PLoS ONE 7, e38571 (2012). 22701672 10.1371/journal.pone.0038571 PMC3368933

[b27] Naas A. E. *et al.* Do rumen *Bacteroidetes* utilize an alternative mechanism for cellulose degradation? mBio 5, e01401–e01414 (2014). 25096880 10.1128/mBio.01401-14 PMC4128358

[b28] Bryant M. P. & Small N. Characteristics of two new genera of anaerobic curved rods isolated from the rumen of cattle. J. Bacteriol. 72, 22–26 (1956). 13345770 10.1128/jb.72.1.22-26.1956 PMC289716

[b29] Strobel H. J. Vitamin B_12_-dependent propionate production by the ruminal bacterium *Prevotella ruminicola* 23. Appl. Environ. Microbiol. 58, 2331–2333 (1992). 1637169 10.1128/aem.58.7.2331-2333.1992 PMC195777

[b30] Suen G. *et al.* The complete genome sequence of *Fibrobacter succinogenes* S85 reveals a cellulolytic and metabolic specialist. PLoS ONE 6, e18814 (2011). 21526192 10.1371/journal.pone.0018814 PMC3079729

[b31] Hooper L. V., Littman D. R. & Macpherson A. J. Interactions between the microbiota and the immune system. Science 336, 1268–1273 (2012). 22674334 10.1126/science.1223490 PMC4420145

[b32] Newbold C. J., Lassalas B. & Jouany J. P. The importance of methanogens associated with ciliate protozoa in ruminal methane production *in vitro*. Lett. Appl. Microbiol. 21, 230–234 (1995). 7576513 10.1111/j.1472-765x.1995.tb01048.x

[b33] Hackstein J. H. P. in (Endo)symbiotic Methanogenic Archaea Microbiology Monographs 19 (ed. Hackstein J. H. P.) 13–23 (Springer-Verlag Berlin Heidelberg, 2010).

[b34] Ozutsumi Y., Tajima K., Takenaka A. & Itabashi H. Real-time PCR detection of the effects of protozoa on rumen bacteria in cattle. Curr. Microbiol. 52, 158–162 (2006). 16467991 10.1007/s00284-005-0266-9

[b35] Paul K., Nonoh J. O., Mikulski L. & Brune A. “*Methanoplasmatales*”, *Thermoplasmatales*-related archaea in termite guts and other environments, are the seventh order of methanogens. Appl. Environ. Microbiol. 78, 8245–8253 (2012). 23001661 10.1128/AEM.02193-12 PMC3497382

[b36] Miller T. L. & Wolin M. J. *Methanosphaera stadtmaniae* gen. nov., sp. nov.: a species that forms methane by reducing methanol with hydrogen. Arch. Microbiol. 141, 116–122 (1985). 3994486 10.1007/BF00423270

[b37] Dehority B. A. Pectin-fermenting bacteria isolated from the bovine rumen. J. Bacteriol. 99, 189–196 (1969). 5802604 10.1128/jb.99.1.189-196.1969 PMC249986

[b38] Stams A. J. & Plugge C. M. Electron transfer in syntrophic communities of anaerobic bacteria and archaea. Nat. Rev. Microbiol. 7, 568–577 (2009). 19609258 10.1038/nrmicro2166

[b39] Bohlken H. Remarks on the stomach and the systematic position of the *Tylopoda*. J. Zool. 134, 207–215 (2009).

[b40] Henderson G. *et al.* Effect of DNA extraction methods and sampling techniques on the apparent structure of cow and sheep rumen microbial communities. PLoS ONE 8, e74787 (2013). 24040342 10.1371/journal.pone.0074787 PMC3770609

[b41] Tymensen L. D. & McAllister T. A. Community structure analysis of methanogens associated with rumen protozoa reveals bias in universal archaeal primers. Appl. Environ. Microbiol. 78, 4051–4056 (2012). 22447586 10.1128/AEM.07994-11 PMC3346394

[b42] Rius A. G. *et al.* Nitrogen metabolism and rumen microbial enumeration in lactating cows with divergent residual feed intake fed high-digestibility pasture. J.Dairy Sci. 95, 5024–5034 (2012). 22916906 10.3168/jds.2012-5392

[b43] Caporaso J. G. *et al.* QIIME allows analysis of high-throughput community sequencing data. Nat. Methods 7, 335–336 (2010). 20383131 10.1038/nmeth.f.303 PMC3156573

[b44] Edgar R. C. Search and clustering orders of magnitude faster than BLAST. Bioinformatics 26, 2460–2461 (2010). 20709691 10.1093/bioinformatics/btq461

[b45] Altschul S. F., Gish W., Miller W., Myers E. W. & Lipman D. J. Basic local alignment search tool. J. Mol. Biol. 215, 403–410 (1990). 2231712 10.1016/S0022-2836(05)80360-2

[b46] Kittelmann S. & Janssen P. H. Characterization of rumen ciliate community composition in domestic sheep, deer, and cattle, feeding on varying diets, by means of PCR-DGGE and clone libraries. FEMS Microbiol. Ecol. 75, 468–481 (2011). 21204869 10.1111/j.1574-6941.2010.01022.x

[b47] Federhen S. Type material in the NCBI Taxonomy Database. Nucleic Acids Res. 43, D1086–D1098 (2014). 25398905 10.1093/nar/gku1127 PMC4383940

[b48] Huber T., Faulkner G. & Hugenholtz P. Bellerophon: a program to detect chimeric sequences in multiple sequence alignments. Bioinformatics 20, 2317–2319 (2004). 15073015 10.1093/bioinformatics/bth226

[b49] Kenters N., Henderson G., Jeyanathan J., Kittelmann S. & Janssen P. H. Isolation of previously uncultured rumen bacteria by dilution to extinction using a new liquid culture medium. J. Microbiol. Methods 84, 52–60 (2011). 21034781 10.1016/j.mimet.2010.10.011

[b50] Rubel F. & Kottek M. Observed and projected climate shifts 1901-2100 depicted by world maps of the Köppen-Geiger climate classification. Meteorol. Z. 19, 134–141 (2010).

[b51] GenStat for Windows 14th Edition (Hemel Hempstead, UK, 2011).

[b52] Core Team. R R: A language and environment for statistical computing. (R Foundation for Statistical Computing, Vienna, Austria, 2014).

[b53] Friedman J. & Alm E. J. Inferring correlation networks from genomic survey data. PLoS Comput. Biol. 8, e1002687 (2012). 23028285 10.1371/journal.pcbi.1002687 PMC3447976

[b54] González I., Lê Cao K.-A., Davis M. J. & Déjean S. Visualising associations between paired ‘omics’ data sets. BioData Min. 5, 19 (2012). 23148523 10.1186/1756-0381-5-19 PMC3630015

