# Erratum: Rumen microbial community composition varies with diet and host, but a core microbiome is found across a wide geographical range

**DOI:** 10.1038/srep19175

**Published:** 2016-01-20

**Authors:** Gemma Henderson, Faith Cox, Siva Ganesh, Arjan Jonker, Wayne Young, Global Rumen Census Collaborators, Leticia Abecia, Erika Angarita, Paula Aravena, Graciela Nora Arenas, Claudia Ariza, Graeme T. Attwood, Jose Mauricio Avila, Jorge Avila-Stagno, André Bannink, Rolando Barahona, Mariano Batistotti, Mads F. Bertelsen, Aya Brown-Kav, Andres M. Carvajal, Laura Cersosimo, Alexandre Vieira Chaves, John Church, Nicholas Clipson, Mario A. Cobos-Peralta, Adrian L. Cookson, Silvio Cravero, Omar Cristobal Carballo, Katie Crosley, Gustavo Cruz, María Cerón Cucchi, Rodrigo de la Barra, Alexandre B. De Menezes, Edenio Detmann, Kasper Dieho, Jan Dijkstra, William L. S. dos Reis, Mike E. R. Dugan, Seyed Hadi Ebrahimi, Emma Eythórsdóttir, Fabian Nde Fon, Martín Fraga, Francisco Franco, Chris Friedeman, Naoki Fukuma, Dragana Gagić, Isabelle Gangnat, Diego Javier Grilli, Le Luo Guan, Vahideh Heidarian Miri, Emma Hernandez-Sanabria, Alma Ximena Ibarra Gomez, Olubukola A. Isah, Suzanne Ishaq, Elie Jami, Juan Jelincic, Juha Kantanen, William J. Kelly, Seon-Ho Kim, Athol Klieve, Yasuo Kobayashi, Satoshi Koike, Jan Kopecny, Torsten Nygaard Kristensen, Sophie Julie Krizsan, Hannah LaChance, Medora Lachman, William R. Lamberson, Suzanne Lambie, Jan Lassen, Sinead C. Leahy, Sang-Suk Lee, Florian Leiber, Eva Lewis, Bo Lin, Raúl Lira, Peter Lund, Edgar Macipe, Lovelia L. Mamuad, Hilário Cuquetto Mantovani, Gisela Ariana Marcoppido, Cristian Márquez, Cécile Martin, Gonzalo Martinez, Maria Eugenia Martinez, Olga Lucía Mayorga, Tim A. McAllister, Chris McSweeney, Lorena Mestre, Elena Minnee, Makoto Mitsumori, Itzhak Mizrahi, Isabel Molina, Andreas Muenger, Camila Muñoz, Bostjan Murovec, John Newbold, Victor Nsereko, Michael O’Donovan, Sunday Okunade, Brendan O’Neill, Sonia Ospina, Diane Ouwerkerk, Diana Parra, Luiz Gustavo Ribeiro Pereira, Cesar Pinares-Patiño, Phil B. Pope, Morten Poulsen, Markus Rodehutscord, Tatiana Rodriguez, Kunihiko Saito, Francisco Sales, Catherine Sauer, Kevin Shingfield, Noriaki Shoji, Jiri Simunek, Zorica Stojanović-Radić, Blaz Stres, Xuezhao Sun, Jeffery Swartz, Zhi Liang Tan, Ilma Tapio, Tasia M. Taxis, Nigel Tomkins, Emilio Ungerfeld, Reza Valizadeh, Peter van Adrichem, Jonathan Van Hamme, Woulter Van Hoven, Garry Waghorn, R. John Wallace, Min Wang, Sinéad M. Waters, Kate Keogh, Maren Witzig, Andre-Denis G. Wright, Hidehisa Yamano, Tianhai Yan, David R. Yáñez-Ruiz, Carl J. Yeoman, Ricardo Zambrano, Johanna Zeitz, Mi Zhou, Hua Wei Zhou, Cai Xia Zou, Pablo Zunino, Peter H. Janssen

Scientific Reports 5: Article number: 14567; 10.1038/srep14567 published online: 10092015; updated: 01202016.

The original version of this Article contained typographical errors in the spelling of the authors David R. Yáñez-Ruiz, Cesar Pinares-Patiño and Camila Muñoz, which were incorrectly given as David R. Yanez-Ruiz, Cesar Pinares-Patino and Camila Munoz respectively.

In addition, Leticia Abecia, Gonzalo Martinez and David R. Yáñez-Ruiz were incorrectly listed as being affiliated with ‘AgResearch Limited, Grasslands Research Centre, Palmerston North 4442, New Zealand’. The correct affiliation is listed below:

Estacion Experimental del Zaidin, Consejo Superior de Investigaciones Cientificas, 18100 Armilla, Granada, Spain

Erika Angarita, Claudia Ariza, Edgar Macipe, Olga Lucía Mayorga, Lorena Mestre, Sonia Ospina, Tatiana Rodriguez and Ricardo Zambrano were incorrectly listed as being affiliated with ‘Estacion Experimental del Zaidin, Consejo Superior de Investigaciones Cientificas, 18100 Armilla, Granada, Spain’. The correct affiliation is listed below:

Animal Nutrition, Biotechnology and Bioindustry Center, Corporación Colombiana de Investigaciones Agropecuarias, Bogota, Colombia

Paula Aravena and Jorge Avila-Stagno were incorrectly listed as being affiliated with ‘Animal Nutrition, Biotechnology and Bioindustry Center, Corporación Colombiana de Investigaciones Agropecuarias, Bogota, Colombia’. The correct affiliation is listed below:

Facultad de Ciencias Veterinarias, Universidad de Concepción, Chillan, Chile

Graciela Nora Arenas & Diego Javier Grilli were incorrectly listed as being affiliated with ‘Facultad de Ciencias Veterinarias, Universidad de Concepción, Chillan, Chile’. The correct affiliation is listed below:

Área Microbiología, Departamento de Patología, Facultad de Ciencias Médicas, Universidad Nacional de Cuyo, Centro Universitario, Parque General San Martín S/N, 5500 Mendoza, Argentina

Graeme T. Attwood, Mariano Batistotti, Adrian L. Cookson, Dragana Gagić, William J. Kelly, Suzanne Lambie, Sinead C. Leahy, Cesar Pinares-Patiño and Xuezhao Sun were incorrectly listed as being affiliated with ‘Área Microbiología, Departamento de Patología, Facultad de Ciencias Médicas, Universidad Nacional de Cuyo, Centro Universitario, Parque General San Martín S/N, 5500 Mendoza, Argentina’. The correct affiliation is listed below:

AgResearch Limited, Grasslands Research Centre, Palmerston North 4442, New Zealand

In addition, there was a typographical error in Affiliation 39 which was incorrectly listed as ‘Department of Animal Science & Technology, Sunchon National University, Suncheon, Jeonnam 540–743, Korea’. The correct affiliation is listed below:

Department of Animal Science & Technology, Sunchon National University, Suncheon, Jeonnam 540–742, Korea

Lastly, there was a typographical error in the project number in the Acknowledgements section.

“Cooperative Research Program for Agriculture Science & Technology Development (project number PJ0074512012)”

now reads:

“Cooperative Research Program for Agriculture Science & Technology Development (project number PJ010906)”

These errors have now been corrected in the PDF and HTML versions of the Article.

