# Supplementary material for: Rumen microbial community composition varies with diet and host, but a core microbiome is found across a wide geographical range
Source: Sci Rep. 2015 Oct 9;5:14567. doi: 10.1038/srep14567 (PMC4598811; doi:10.1038/srep14567)
Supplement: Supplementary Information [file srep14567-s1.pdf]

Supplementary Information for

Rumen microbial community composition varies with diet and host, but a core microbiome is found across a wide geographical range

Gemma Henderson, Faith Cox, Siva Ganesh, Arjan Jonker, Wayne Young, Global Rumen Census Collaborators, and Peter H. Janssen

Correspondence to: peter.janssen@agresearch.co.nz, gemma.henderson@agresearch.co.nz

This file includes:

Supplementary Text 1.

Supplementary Fig. 1. Relative abundances of dominant bacteria, archaea, and protozoa.

Supplementary Fig. 2. Variability of bacterial, archaeal, and protozoal communities.

Supplementary Fig. 3. Effect of host species and dietary forage to concentrate ratios on rumen microbial communities.

Supplementary Fig. 4. Effect of forage to concentrate ratios on A) bacterial, B) archaeal, and C) protozoal communities.

Supplementary Fig. 5 Effect of host lineage on A) bacterial, B) archaeal, and C) protozoal communities.

Supplementary Fig. 6. Effect of forage type on A) bacterial, B) archaeal, and C) protozoal communities.

Supplementary Fig. 7. Correlations between major rumen bacteria, archaea, and protozoa.

Supplementary Fig. 8. Reproducibility of the sample processing pipeline.

Supplementary Fig. 9. Effect of forage plant on A) bacterial, B) archaeal, and C) protozoal communities.

Supplementary Table 1. The most abundant and prevalent rumen bacterial (Ba) genus-level groups.

Supplementary Table 2. Mean relative abundances of bacterial groups summarised by animal species group.

Supplementary Table 3. The most abundant and prevalent rumen archaeal (Ar) species-level groups.

Supplementary Table 4. Mean relative abundances of archaeal groups summarised by animal species group.

Supplementary Table 5. Metabolic classification of rumen methanogens.

Supplementary Table 6. The most abundant and prevalent rumen protozoal (Pr) genus-level groups.

Supplementary Table 7. Diet classifications.

Supplementary Table 8. Identities of the 50 most abundant and prevalent bacterial operational taxonomic units (OTUs[a]).

Supplementary Table 9. Identities of the 50 most abundant and prevalent archaeal operational taxonomic units (OTUs[a]).

Supplementary Table 10. Bacterial groups considered to be exogenous to the rumen.

Other Supplementary Information for this manuscript includes the following:

Supplementary Data 1 as an Excel file: Supplementary _Data_1.xlsx

Supplementary Text 1

Supplementary Methods

Sample processing pipeline reproducibility and negative controls

Due to the high number of samples, these were processed in four batches (and sequencing rounds at MWG Biotech). We assessed the reproducibility of the DNA extraction, PCR, and sequencing steps using 12 rumen samples (sample numbers 0333-0336, 0468-0471, 0544, and 0546-0548; Supplementary Fig. 8). These samples were originally processed in sequencing round 2. In sequencing round 3, the samples were reanalysed in three ways: by starting at the DNA extraction step using the homogenised rumen sample from sequencing round 2 (DNA.REP); starting at the PCR step using the DNA extract from sequencing round 2 (PCR.REP); and re-sequencing the PCR product from sequencing round 2 (SEQ.REP). Additionally, DNA was extracted from these samples using the RBB+C method of Yu and Morrison [1] from 30 mg of freeze-dried samples as previously described [2] (RBBC). A negative control sample, which was subject to all sample processing steps, was also sequenced.

Criteria for sample exclusion

Samples were excluded from analyses if an insufficient number of sequencing reads were obtained. Five samples with less than 500 sequencing reads of bacterial 16S rRNA genes were excluded, for archaea 38 samples with less than 100 sequencing

reads of archaeal 16S rRNA genes were excluded, and 150 samples with less than 100 sequencing reads of protozoal 18S rRNA genes were excluded. These exclusions were done independently, so that a sample was only excluded in the dataset for the microbial group (i. e., bacteria, archaea, or protozoa) that failed to reach this minimum, but retained for the others for which sufficient read data were obtained. In most of these cases, PCR amplification yielded less PCR product, but the amount of this product that was used for sequencing was increased so that it was the same for all samples. There were no obvious factors that caused bacterial, archaeal, or protozoal reads to be low for some samples.

Differentiation of rare and outlier taxa from noise

To be included in analyses, microbial taxa (hereafter referred to as groups) had to have a relative abundance greater than 0.5% in at least one of the 742 samples. Groups that fulfilled this criterion represent 99.6, 100.0, and 100.0% of sequences of bacteria, archaea, and protozoa, respectively (Supplementary Tables 1, 3, and 5). Groups that did not meet this criterion were summarised as “other groups”. The relative abundance of these “other groups” in any one sample was at most 2.8, 0.5, and 0.0% of the bacterial, archaeal, and protozoal communities, respectively.

Some samples contained high levels of potentially exogenous bacteria that are not typically found in the rumen (Supplementary Table 10). Many of these exogenous bacteria may have “legitimately” entered the rumen via the ingestion of feed, water, and soil and could thus be considered transient residents of the rumen. However, some exogenous bacteria may have increased or been introduced during sample processing, for example, due to prolonged storage without adequate cooling, and

should thus be considered as sample contaminants. It was difficult to decide whether bacterial groups were transient rumen residents or contaminants, thus these were summarised as “exogenous groups”. On average these “exogenous groups” comprised 3.0±0.4% of bacterial communities and were found in 88% (*n* = 737) of samples. Samples (*n* = 53) with a relative abundance of “exogenous groups” greater than 10% were excluded from further analyses. On the whole, less DNA was extracted from these samples, which could be due to degradation of the DNA during sample processing or the presence of substances that may interfere with DNA extraction.

Archaeal and protozoal communities of samples that contained high abundances of exogenous bacterial groups appeared to be comparable with those of other samples. This is possibly because archaea and protozoa are not ubiquitous environmental microbes, and they are less likely than bacteria to enter the rumen as transients, contaminate the samples, or be introduced during sample processing. For these reasons, we used archaeal or protozoal data from these samples.

Supplementary Results

Sample processing pipeline reproducibility and negative controls

Principal coordinate analysis of microbial community composition was used to assess the reproducibility of the DNA extraction, PCR, and sequencing steps of the sample processing pipeline (Supplementary Fig. 8). As expected, data generated from the same samples were more similar if fewer steps of the pipeline were repeated, and if the same rather than a different DNA extraction method was used. The smallest difference was observed between different sequencing rounds on the same PCR product (SEQ.REP; see Supplementary Fig. 8), with greater differences with different PCRs on the same DNA (PCR.REP), even greater with replicate DNA extractions using the same (PCQI) method on the same sample (DNA.REP), and the greatest with a different DNA extraction method on the same samples (RBBC). Even so, overall, reproducibility was high (Supplementary Fig. 8). Samples from pasture-fed deer, pasture-fed Belgian Blue cattle, and concentrate/silage-fed Icelandic cattle generally formed distinct clusters by sample type rather than by variations in sample processing and data generation. Data points from the same animals clustered together closely in the majority of cases, the few exceptions nonetheless indicating that some variability can be introduced during sample processing. For example, the protozoal community results were quite disparate for deer rumen sample GRC0467 (sample ii), possibly due to repetition of the DNA extraction and PCR amplification steps resulting in fewer protozoal sequencing reads being obtained. Repeating the sequencing on the same PCR sample that had been stored at -20°C for two months generally resulted in fewer sequencing reads being obtained. In some cases, e. g., archaea in some of the Icelandic cattle and bacteria in some of the deer, samples

from different animals processed using the RBB+C DNA extraction method grouped more closely together than samples from the same animals. It has previously been shown that changes in sample processing and DNA extraction methods can result in differences in apparent microbial community compositions [2]. This study confirms this finding, and it also provides evidence that performing DNA extraction and PCR steps on separate days can result in subtle changes being introduced to apparent microbial community compositions. However, compared to the variation of the whole dataset, these changes were minor. Therefore, despite subtle differences in sample processing, the data generated by this study can validly be used to survey the diversity of rumen microbial communities.

No products were observed from negative controls following PCR amplification, and no sequencing reads were obtained for archaea or protozoa, and only ten for bacteria. These bacterial reads were assigned to the *Lachnospiraceae* (4 reads), *Pseudomonas* (3 reads), *Treponema* (1 read), *Clostridiales* (1 read), and *Bacteroidales* (1 read), and thus possibly stemmed from extremely low-level background (rumen) environmental contamination or were due to barcode sequencing errors.

Transient bacteria found potentially originating from feed and soil

Grazing ruminants are observed to ingest soil [3], and soil microbes would thus enter the rumen. However, sequences from highly diagnostic groups of soil bacteria were very rare in the dataset. Bacteria from classes (subdivisions) 1, 3, 4, and 6 of the phylum *Acidobacteria*, from the classes *Acidimicrobiia*, *Rubrobacteria*, and *Thermoleophilia* of the phylum *Actinobacteria*, from the class *Flavobacteria* of the

phylum *Bacteroidetes*, and from classes *Spartobacteria* and 3 of the phylum *Verrucomicrobia* are globally distributed and ubiquitous soil bacteria [4]. Sequences from these groups made up 32.5% of 16S rRNA gene sequences in soil [4], but only 0.007% of the sequences from the rumen samples. Bacteria of the order *Actinomycetales* of the phylum *Actinobacteria* are also considered to be soil bacteria, with an abundance of about 4.7% [4]. These were far more abundant in the rumen samples than other soil bacteria, but still only made up 0.5% of all sequences. The discrepancy between their abundance and that of other soil bacteria suggests a different source for *Actinomycetales*. These bacteria are common airborne contaminants and feed spoilage organisms [5], and may have been ingested with feed rather than soil. Overall, the data suggest that only a very small part of the rumen microbial community is made up of transients from feed, and even fewer from soil.

Members of the class *Bacilli*, such as *Carnobacterium*, *Enterococcus*, *Lactobacillus*, and *Streptococcus*, were generally not prevalent and abundant, but are known to occur in the rumen, are used as silage inoculants, and are sometimes associated with acidosis [6].

*Additional factors influencing microbial communities (*Supplementary Figs. *3, 4, 5, 6, and 9)*

Within those animals consuming diets containing at least 30% forage, higher relative abundances of unclassified *Succinivibrionaceae*, *Ruminococcus*, *Prevotella*, *Butyrivibrio*, *Coprococcus*, and *Methanomassiliicoccaceae*-affiliated group 12 sp. ISO4-H5 were characteristic of animals fed preserved whole crops such as silage, whereas *Pseudobutyrivibrio* and *Selenomonas* were more abundant in animals fed

diets comprised of both fresh and preserved whole crops. Grasses were the most widely used forage plant and unclassified *Clostridiales*, *Mogibacteriaceae, Christensenellaceae*, and *Ruminococcaceae*, as well as the *Methanobrevibacter gottschalkii* clade, were typically found in ruminants fed pasture. When grass forage was supplemented with another forage derived from a cereal plant, unclassified *Lachnospiraceae* and *Butyrivibrio*, *Coprococcus*, and *Prevotella* became more evident, as did members of the archaeal *Methanomassiliicoccaceae*. Samples from animals on diets with high levels of starch were characterised by *Prevotella*, *Methanomassiliicoccaceae* group 12 sp. ISO4-H5, and to some extent *Entodinium*, whereas those with high levels of pectin were typified by *Succiniclasticum*, *Entodinium*, *Ophryoscolex*, and *Methanosphaera* sp. ISO3-F5. Pectin is a rich source of microbially-available methyl groups [7] and *Methanosphaera* spp. are known to use methyl groups for methane formation [8]. Samples from animals on diets that contained low levels of starch and pectin could be discriminated by the greater relative abundances of unclassified *Clostridiales*, *Christensenellaceae*, and *Ruminococcaceae*, the *Methanobrevibacter gottschalkii* clade, and *Eudiplodinium*.

Co-occurrence and associations within and between rumen microbial communities

Prevotella showed strong negative correlations with other major bacterial groups, but this is probably because they are a large proportion of the total, and changes in their abundance will have arithmetic effects when all data as expressed as proportions of the total bacterial community. Similarly, the two main archaeal groups, *Methanobrevibacter gottschalkii* and *Methanobrevibacter ruminantium* display this replacement effect. The same effect was observed within the protozoa, where the

abundant genus *Entodinium* was found to correlate negatively with other abundant protozoal groups.

Supplementary Acknowledgements

We thank the following, who provided assistance that allowed Global Rumen Census collaborators to supply samples and metadata: Andre Abraham (Northstar Bison Processing), Seppo Ahvenjärvi (Natural Resources Institute Finland), Ole Sommer Bach (Randers Rainforest), Tim Bell (Swiss Federal Institute of Technology), Maria Benson (University College Dublin), Laurence Bernard (Institut National de la Recherche Agronomique), Jane Ann Boles (Montana State University), Cecilia Cajarville (Facultad de Veterinaria, Universidad de la República), Fergal Coughlan (Teagasc), Joost de Groot (Cargill Animal Nutrition), Michel Doreau (Institut National de la Recherche Agronomique), Obioha N. Durunna (The University of Queensland), Vanina Egea (Instituto Argentino de Investigaciones en Zonas Áridas), Tryggvi Eiríksson (Agricultural University of Iceland), Conrad Ferris (Agri-Food and Biosciences Institute), Diego Franco (Instituto Nacional de Tecnología Agropecuaria, Argentina), Lucette Genestoux (Institut National de la Recherche Agronomique), Marziye Ghadami (Ferdowsi University of Mashhad), Pablo Gutierrez-Toral (Institut National de la Recherche Agronomique), Grétar H. Harðarson (Agricultural University of Iceland), Joe Heemskerk (Westwold View Farms), Lisbeth Høgh (Knuthenborg Safari Park), Keith Joblin (AgResearch Ltd), Fumiko Kasai (Chiba Prefectural Livestock Research Center), Dave Kasper (Cargill Animal Nutrition), Kenji Kawashima (Chiba Prefectural Livestock Research Center), David Kenny (Teagasc), Kate Keogh (Teagasc), Amil Kleinert (Memphis Meat Processing), Larry Kolles (Cargill Animal Nutrition), Andrej Lavrencic (University of Ljubljana), Rasmus Levinsen (Mølgaarde), Matevz Likar (University of Ljubljana), Jason Lin (Diamond V), Mahdi Madaeni (Ferdowsi University of Mashhad), Anita Maguire (Department of Agriculture, Fisheries and Forestry), C. Mapiye (Agriculture & Agri-Food Canada),

Marcos Inácio Marcondes (Universidade Federal de Viçosa), Samuel Martin (Agri-Food and Biosciences Institute), Ignacio Martin (Consejo Superior de Investigaciones Cientificas), Sarah Meale (Agriculture & Agri-Food Canada), Roberta Mila-Arango (Colegio de Posgraduados), Stephen Miller (University of Guelph), David Mills (Agri-Food and Biosciences Institute), Ole Mols, Stephen Moore (The University of Queensland), Sithembile Ndlela (University of KwaZulu-Natal), Emma O'Flaherty (The University of Sydney), Ibrahim O. Oguntusi (Federal University of Agriculture), Sebastián Paez (Instituto Argentino de Investigaciones en Zonas Áridas), Simo Pärssinen (HAMK-University of Applied Sciences), Nicolle Pomies (Facultad de Veterinaria, Universidad de la República), Kim Rasmussen (Givskud Zoo), Moufida Rira (Institut National de la Recherche Agronomique), Mayra I. Rivas-Martínez (Colegio de Posgraduados), Dora Raquel Rojas (Instituto Nacional de Tecnología Agropecuaria), Rodolfo Saldaña (Instituto de Investigaciones Agropecuarias, INIA Remehue), Paulino Sánchez Santilla (Colegio de Posgraduados), Jan Šefl (Czech Academy of Sciences), Adriana E. Severianos-Corral (Colegio de Posgraduados), T. Turner (Agriculture & Agri-Food Canada), Urban Udovic (University of Ljubljana), Koichiro Ueda (Hokkaido University), Jan Vávra (Czech Academy of Sciences), Laura Ventto (Natural Resources Institute Finland), Franco Medone Vergara (Instituto de Investigaciones Agropecuarias, INIA Remehue), Darko Veternik (University of Ljubljana), Cristian Villaroel (Instituto de Investigaciones Agropecuarias, INIA Remehue), Tracy Werner (Diamond V), Kelsey Westveer (Montana State University), and Pamela Williams (Universidad de Concepcion).

Supplementary References

1 Yu, Z. & Morrison, M. Improved extraction of PCR-quality community DNA from digesta and fecal samples. *Biotechniques* **36,** 808-812 (2004).

2 Henderson, G. *et al.* Effect of DNA extraction methods and sampling techniques on the apparent structure of cow and sheep rumen microbial communities. *PLoS ONE* **8,** e74787 (2013).

3 Thornton, I. & Abrahams, P. Soil ingestion - a major pathway of heavy metals into livestock grazing contaminated land. *Sci. Total Environ.* **28,** 287-294 (1983).

4 Janssen, P. H. Identifying the dominant soil bacterial taxa in libraries of 16S rRNA and 16S rRNA genes. *Appl. Environ. Microbiol.* **72,** 1719-1728 (2006).

5 Dalphin, J. C. *et al.* Influence of mode of storage and drying of fodder on thermophilic actinomycete aerocontamination in dairy farms of the Doubs region of France. *Thorax* **46,** 619-623 (1991).

6 Petri, R. M. *et al.* Changes in the rumen epimural bacterial diversity of beef cattle as affected by diet and induced ruminal acidosis. *Appl. Environ. Microbiol.* **79,** 3744-3755 (2013).

7 Schink, B., Ward, J. C. & Zeikus, J. G. Microbiology of wetwood: importance of pectin degradation and *Clostridium* species in living trees. *Appl. Environ. Microbiol.* **42,** 526-532 (1981).

8 Fricke, W. F. *et al.* The genome sequence of *Methanosphaera stadtmanae* reveals why this human intestinal archaeon is restricted to methanol and H_2 for methane formation and ATP synthesis. *J. Bacteriol.* **188,** 642-658 (2006).

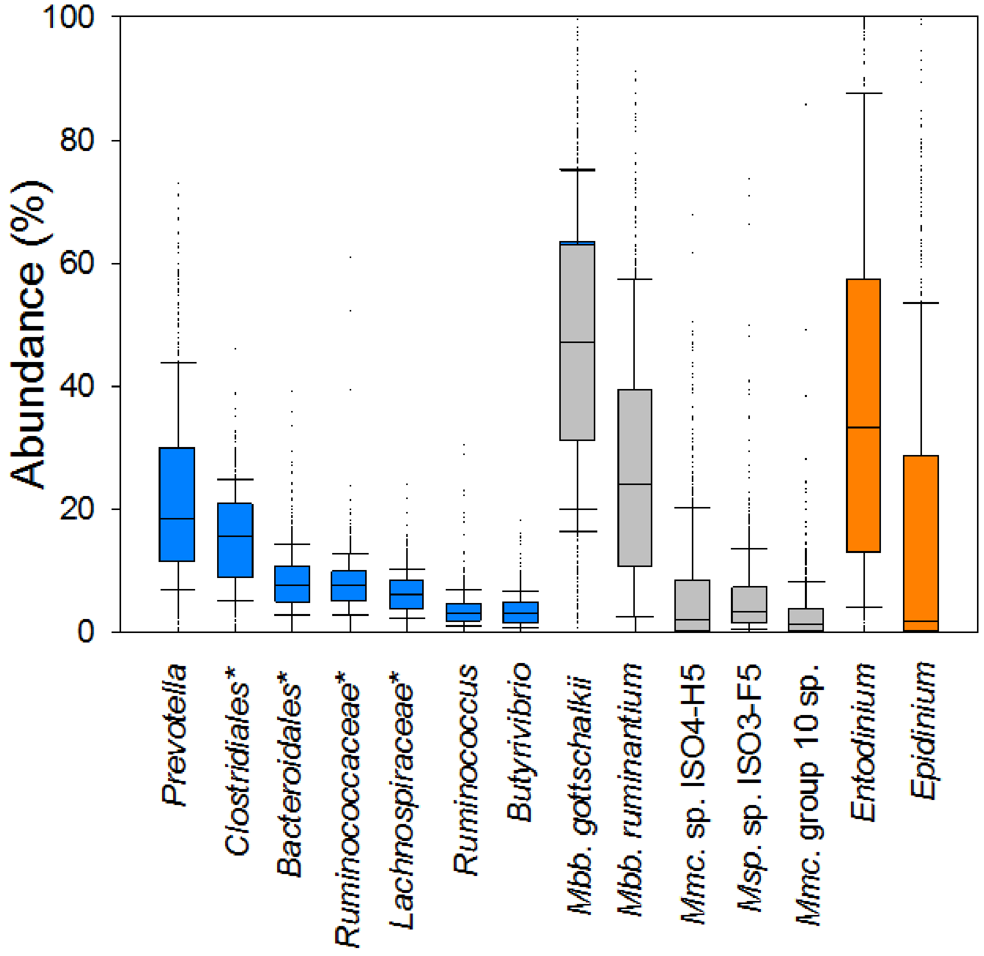

Supplementary Fig. 1

Relative abundances of dominant bacteria, archaea, and protozoa. Boxplots represent the 25th to 75th percentiles, lines within boxes depict medians, whiskers represent the 10th and 90th percentiles, and outliers are plotted as individual points. *indicates unclassified bacteria within an order or family. *Mbb.*, *Methanobrevibacter*; *Mmc.*, *Methanomassiliicoccaceae*; *Msp.*, *Methanosphaera*.

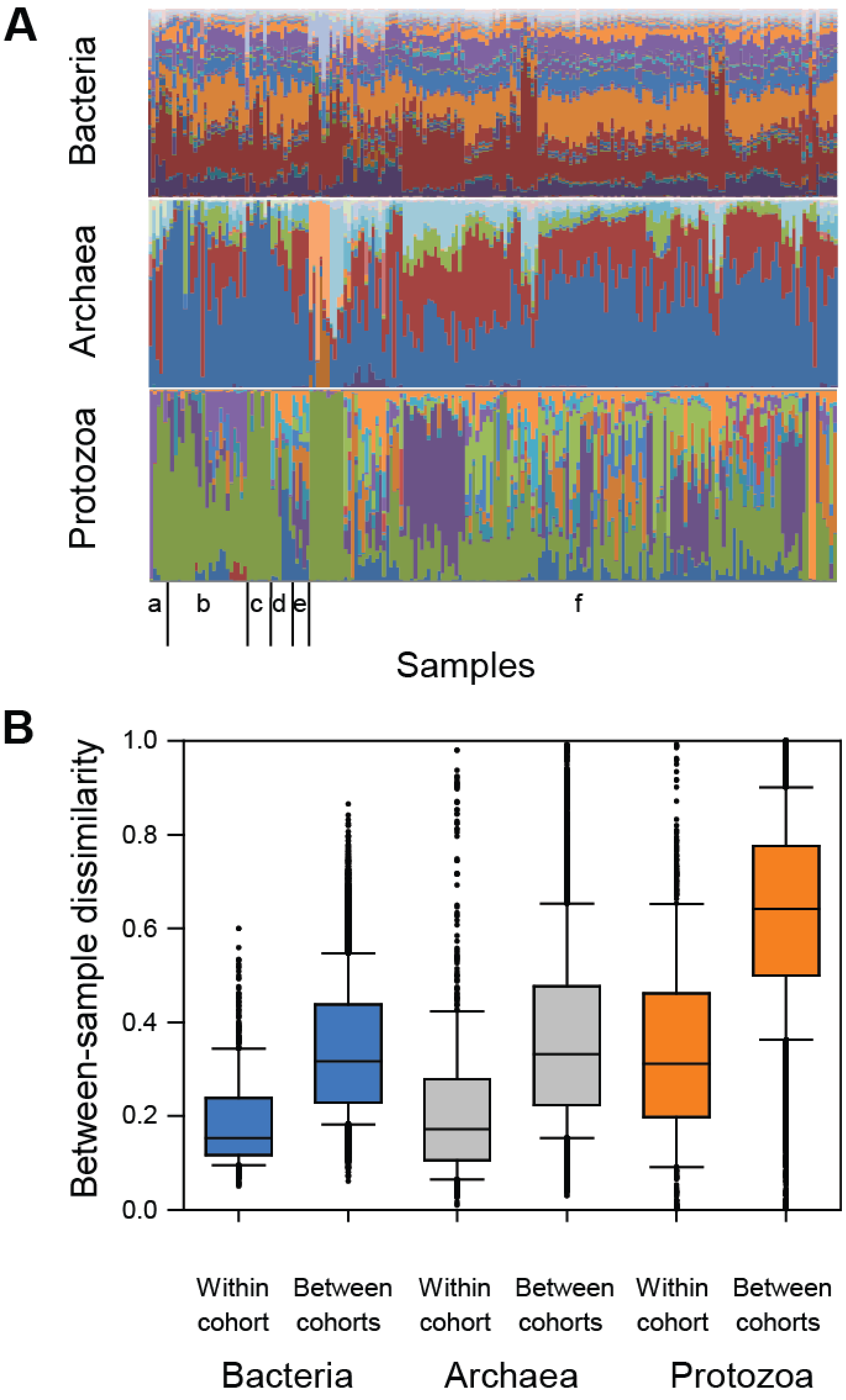
A
Bacteria
Archaea
Protozoa
a
b
c
d
e
f
Samples
B
Between-sample dissimilarity
1.0
0.8
0.6
0.4
0.2
0.0
Within cohort
Between cohorts
Within cohort
Between cohorts
Within cohort
Between cohorts
Bacteria
Archaea
Protozoa

Supplementary Fig. 2

Variability of bacterial, archaeal, and protozoal communities. A) Relative abundances of microbial groups in individual samples (from 24 cohorts with at least five samples, each stacked bar represents one sample, and the colours represent different microbial groups within each panel); Samples were from a, giraffes (n = 5); b, deer (n = 23); c, goats (n = 37); d, sheep (n = 6); e, bison (n = 5); and cattle (n = 152). B) Boxplots showing Bray-Curtis dissimilarities between samples within and between cohorts. Boxplots represent the 25th to 75th percentiles, lines within boxes depict medians, whiskers represent the 10th and 90th percentiles, and outliers are plotted as individual points.

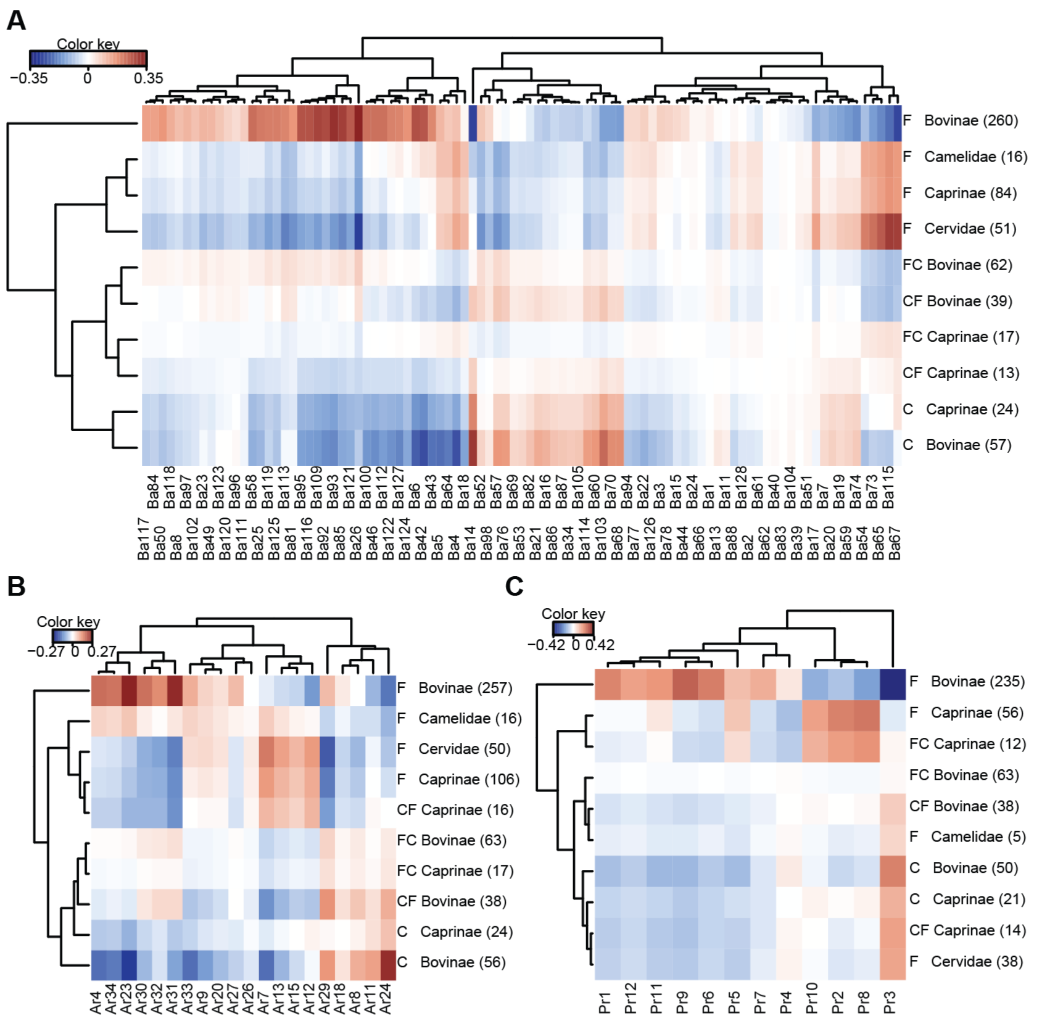

Supplementary Fig. 3

Effect of host species and dietary forage to concentrate ratios on rumen microbial communities. Diets were grouped (Supplementary Table 7) as forage-dominated (F), mixed forage-concentrate (50-70% forage, FC), mixed concentrate-forage (50-70% concentrate, CF), or concentrate-dominated (C). Sparse partial least squares discriminant analysis was used to discriminate between groupings and results represented as heat maps, where the colour represents the association score. Microbial groups (A) bacteria, B) archaea, and C) protozoa) are given on the x-axis, and identified by numbers (Supplementary Tables 1, 3, and 5). The number of samples in each category is in parentheses.

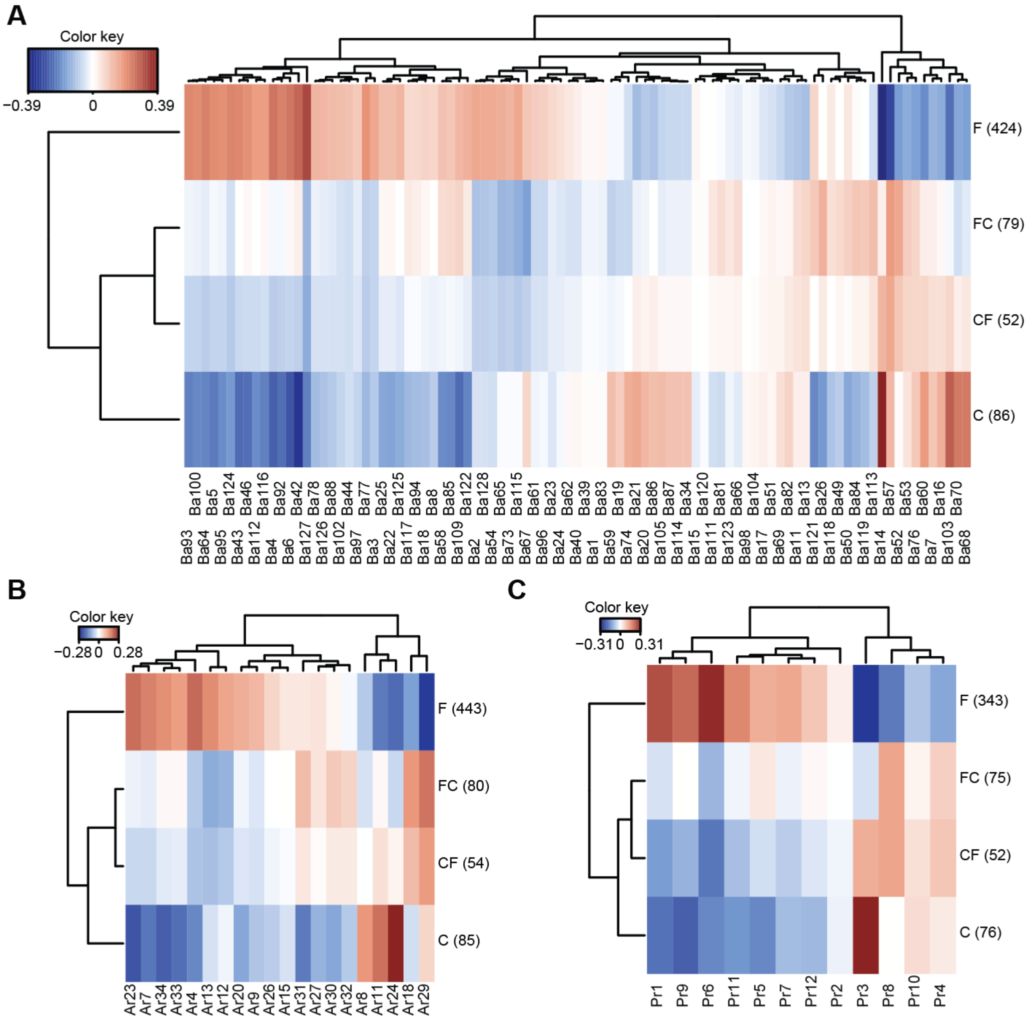

Supplementary Fig. 4

Effect of forage to concentrate ratios on A) bacterial, B) archaeal, and C) protozoal communities. Diets were grouped (Supplementary Table 7) as forage-dominated (F), mixed forage-concentrate (50-70% forage, FC), mixed concentrate-forage (50-70% concentrate, CF), or concentrate-dominated (C). Sparse partial least squares discriminant analysis was used to discriminate between groupings and results represented as heat maps, where the colour represents the association score. Microbial groups are given on the x-axis, and identified by numbers (Supplementary Tables 1, 3, and 5). The number of samples in each category is in parentheses.

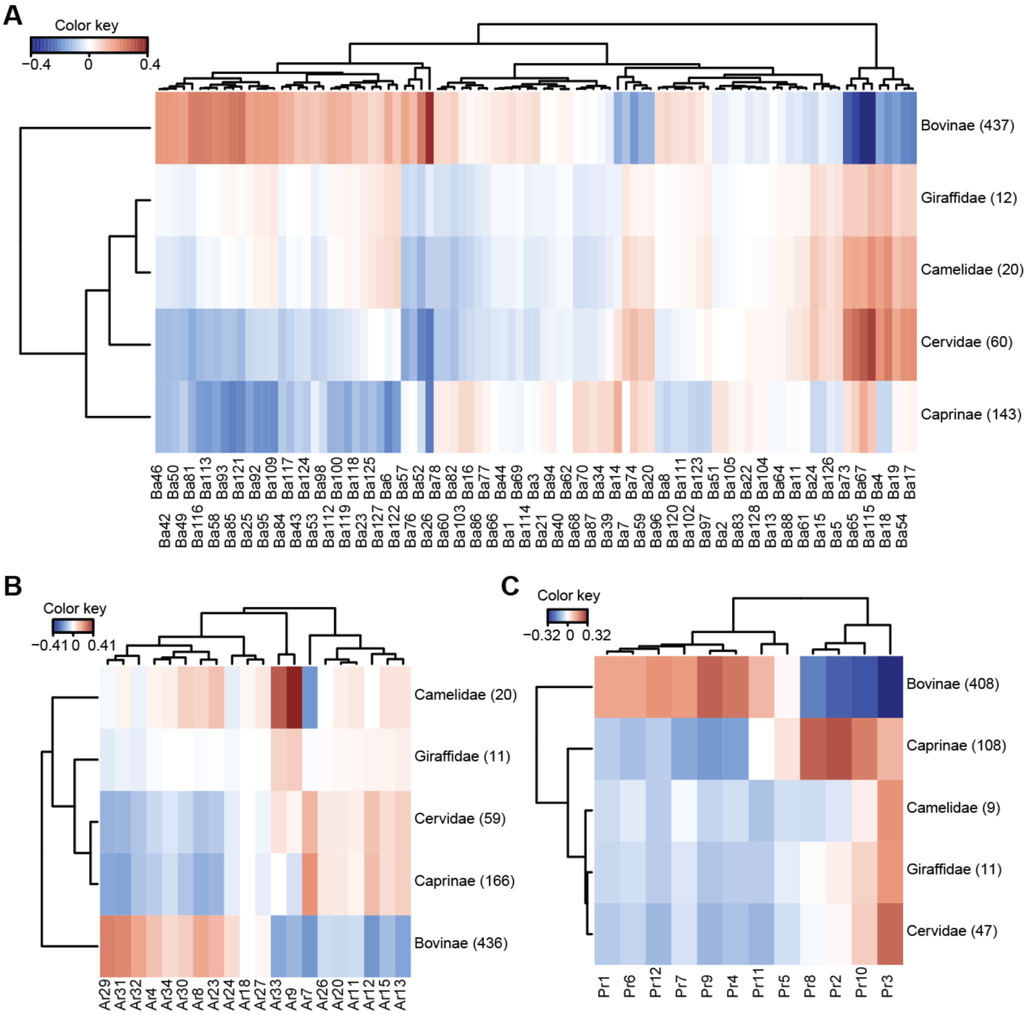

Supplementary Fig. 5

Effect of host lineage on A) bacterial, B) archaeal, and C) protozoal communities. Sparse partial least squares discriminant analysis was used to discriminate between groupings and results represented as heat maps, where the colour represents the association score. Microbial groups are given on the x-axis, and identified by numbers (Supplementary Tables 1, 3, and 5). The number of samples in each category is in parentheses.

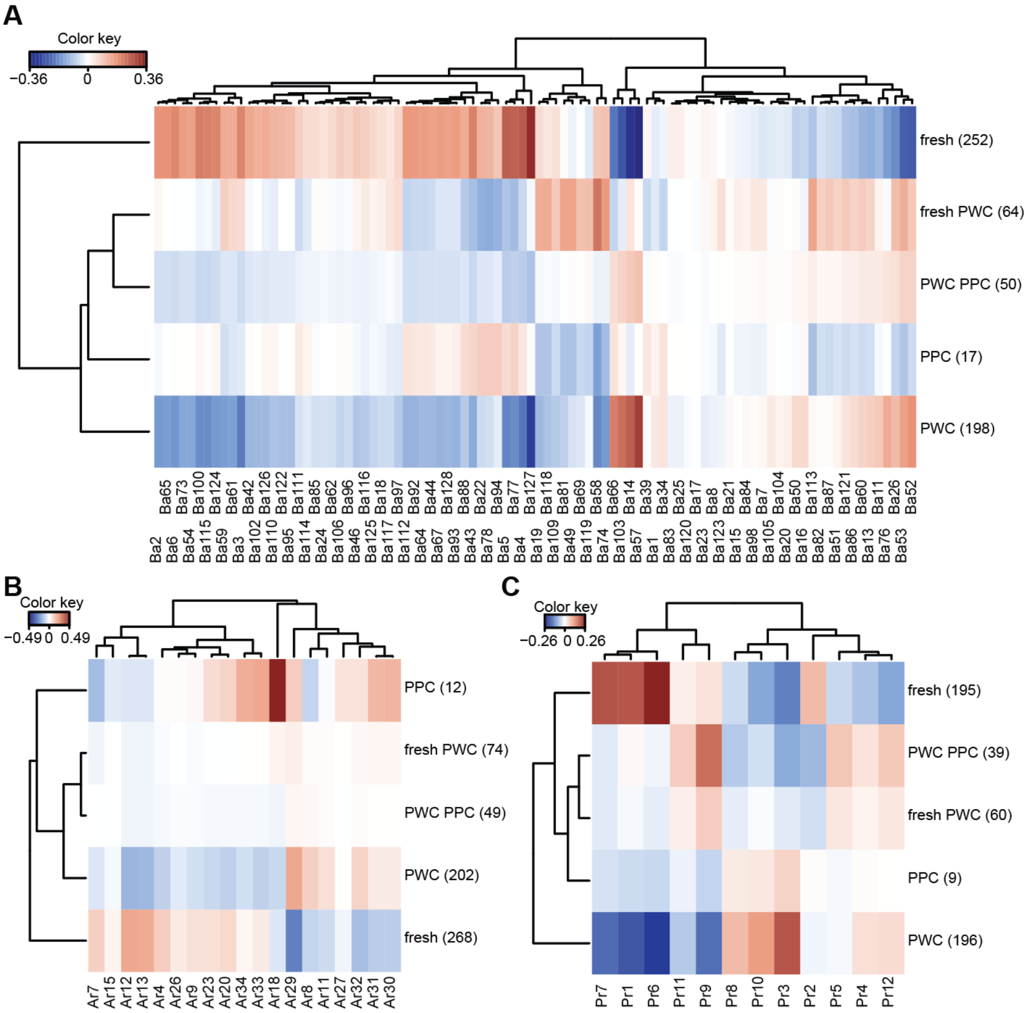

Supplementary Fig. 6

Effect of forage type on A) bacterial, B) archaeal, and C) protozoal communities. Diets were classified according to forage type (Supplementary Table 7), i. e., preserved whole crops (PWC), preserved partial crop residues (PPC), and fresh (e. g., pasture, browse) and combinations. Sparse partial least squares discriminant analysis was used to discriminate between groupings and results represented as heat maps, where the colour represents the association score. Microbial groups are given on the x-axis, and identified by numbers (Supplementary Tables 1, 3, and 5). The number of samples in each category is in parentheses.

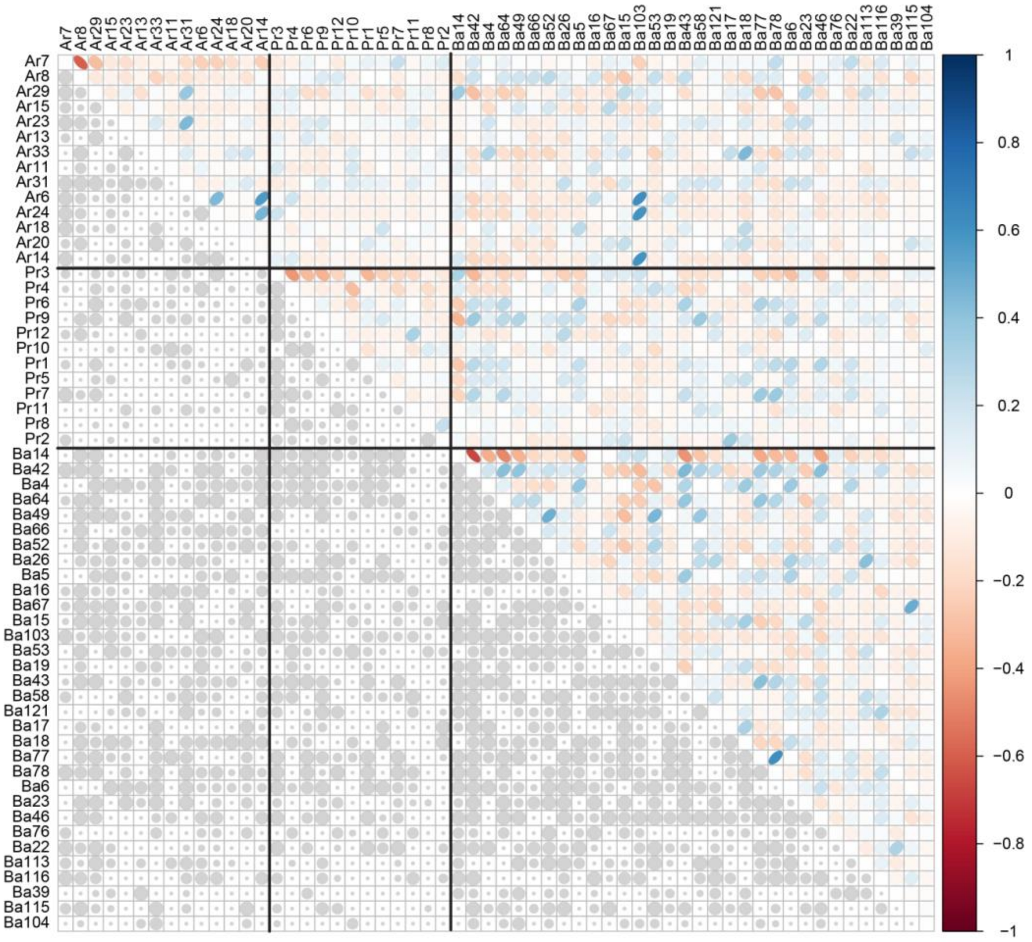

Supplementary Fig. 7

Correlations between major rumen bacteria, archaea, and protozoa. The upper right triangle depicts Pearson correlation coefficients colour-coded to the scale on the right, and the lower left-hand triangle depicts *P* values (the larger the circle, the greater the significance). Microbial groups are identified by numbers (Supplementary Tables 1, 3, and 5).

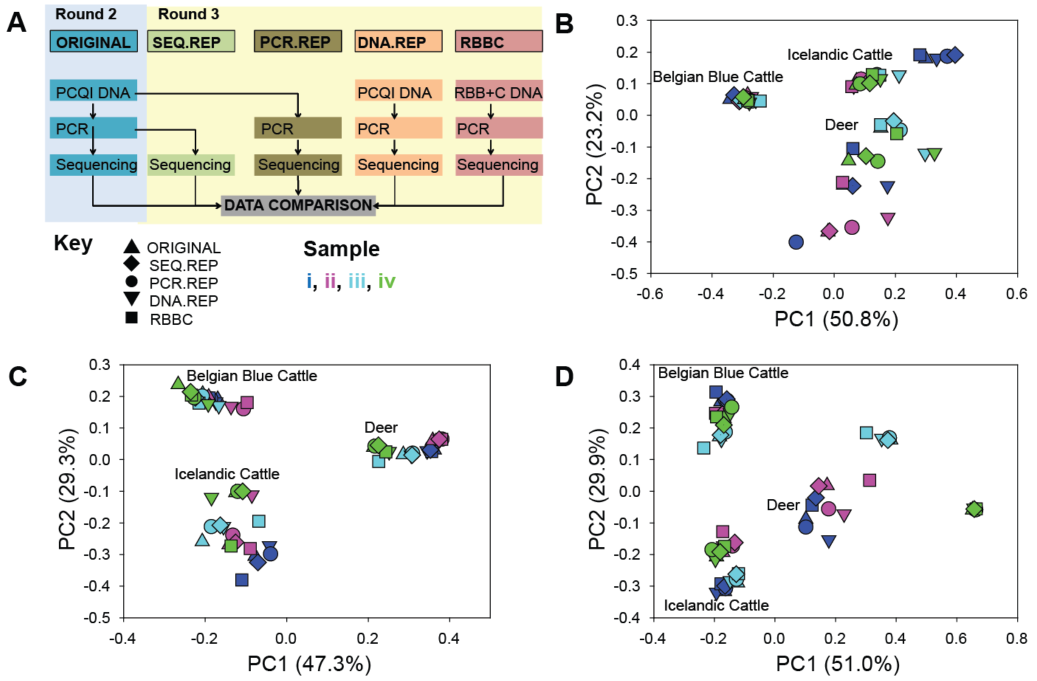

Supplementary Fig. 8

Reproducibility of the sample processing pipeline. The reproducibility of the DNA extraction, PCR and sequencing steps was assessed using three sample cohorts (samples 0333-0336, 0468-0471, and 0544, 0546-0548; see Supplementary Data 1) of four samples each (i, ii, iii, iv). A) These samples were originally processed in sequencing round 2 (ORIGINAL). In sequencing round 3, DNA extraction (DNA.REP), PCR (PCR.REP), and sequencing (SEQ.REP) were repeated for these samples. Additionally, DNA was extracted using the RBB+C (RBBC) method instead of the PCQI method. The resulting B) bacterial, C) archaeal, and D) protozoal community data were compared using principal coordinate analysis. The variation explained by each coordinate is given in parentheses. Microbial community compositions and Bray-Curtis dissimilarity matrices have been provided in Supplementary Data 1.

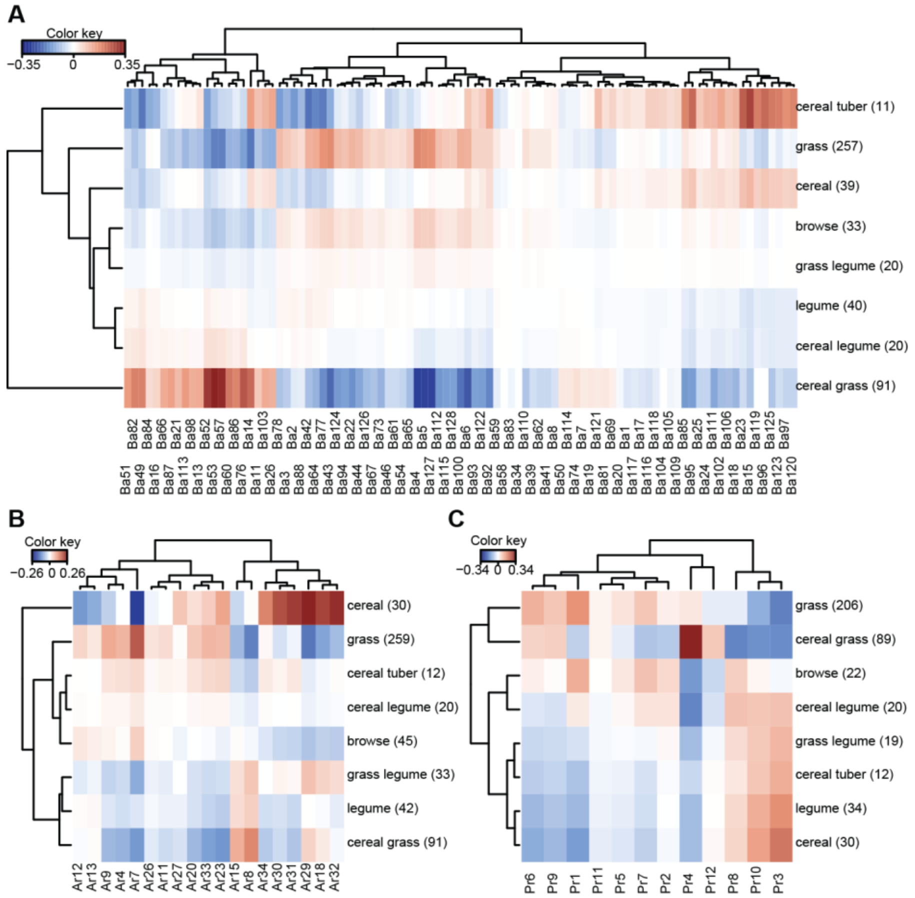

Supplementary Fig. 9

Effect of forage plant on A) bacterial, B) archaeal, and C) protozoal communities. Diets were classified according to forage type (Supplementary Table 7) and combinations. Sparse partial least squares discriminant analysis was used to discriminate between groupings and results represented as heat maps, where the colour represents the association score. Microbial groups are given on the x-axis, and identified by numbers (Supplementary Tables 1, 3, and 5). The number of samples in each category is in parentheses.

Supplementary Table 1

The most abundant and prevalent rumen bacterial (Ba) genus-level groups. The 30 most abundant groups are indicated in bold font.

Ba[a] ID	Group	Mean relative abundance (%, ±SEM[b]) All samples (n = 684)[c]			Adjusted for prevalence[d]			Maximum (%)[e]	Prevalence (%)[f]
1	*Corynebacterium*	0.1	±	0.0	0.3	±	0.0	12.8	37.9
**2**	**Unclassified[g] *Coriobacteriaceae***	0.5	±	0.0	0.5	±	0.0	6.2	97.5
3	*Adlercreutzia*	0.0	±	0.0	0.1	±	0.0	1.0	37.9
**4**	**Unclassified *Bacteroidales***	8.4	±	0.2	8.4	±	0.3	39.2	100
**5**	**BS11 (*Bacteroidales*)**	1.6	±	0.1	1.7	±	0.1	16.6	93.9
**6**	**BF311 (*Bacteroidaceae*)**	0.7	±	0.0	0.8	±	0.0	10.3	89.5
7	*Bacteroides*	0.0	±	0.0	0.0	±	0.0	0.4	49.9
8	Unclassified *Marinilabiaceae*	0.0	±	0.0	0.1	±	0.0	1.1	22.2
9	Unclassified *Porphyromonadaceae*	0.0	±	0.0	0.0	±	0.0	0.2	3.2
10	*Dysgonomonas*	0.0	±	0.0	0.4	±	0.1	4.1	1.6
11	*Paludibacter*	0.1	±	0.0	0.1	±	0.0	2.1	54.5
12	*Parabacteroides*	0.0	±	0.0	0.0	±	0.0	0.1	4.7
13	Unclassified *Prevotellaceae*	0.3	±	0.0	0.3	±	0.0	2.8	93.7
**14**	***Prevotella***	22.0	±	0.6	22.0	±	0.8	72.9	100
**15**	**RF16 (*Bacteroidales*)**	1.3	±	0.1	1.4	±	0.1	15.4	95.5
**16**	**S24-7 (*Bacteroidales*)**	1.6	±	0.1	1.6	±	0.1	18.4	98.8
**17**	**Unclassified [*Paraprevotellaceae*][h]**	0.8	±	0.0	0.8	±	0.0	18.9	98.7
**18**	**CF231 [*Paraprevotellaceae*]**	0.8	±	0.0	0.9	±	0.0	5.7	98.2
**19**	**YRC22 [*Paraprevotellaceae*]**	1.0	±	0.0	1.1	±	0.0	15.4	98.2
20	[*Prevotella*] [*Paraprevotellaceae*]	0.0	±	0.0	0.1	±	0.0	1.8	60.1
21	p-2534-18B5 (*Bacteroidales*)	0.1	±	0.0	0.4	±	0.0	5.8	17.3
**22**	**SHD-231 (*Anaerolinaceae*)**	0.6	±	0.0	0.6	±	0.0	14.3	90.5
**23**	**YS2 (*Cyanobacteria*)**	0.7	±	0.0	0.8	±	0.0	13.2	91.5
24	Unclassified *Elusimicrobiaceae*	0.0	±	0.0	0.0	±	0.0	0.8	32.7
25	Unclassified *Endomicrobia*	0.0	±	0.0	0.1	±	0.0	0.7	38.5

Ba[a] ID	Group	Mean relative abundance (%, ±SEM[b]) All samples (*n* = 684)[c]			Adjusted for prevalence[d]			Maximum (%)[e]	Prevalence (%)[f]
**26**	***Fibrobacter***	2.9	±	0.1	3.1	±	0.1	34.7	93.0
27	Unclassified *Lactobacillales*	0.0	±	0.0	0.1	±	0.0	1.1	4.8
28	*Aerococcus*	0.0	±	0.0	0.1	±	0.0	0.7	2.2
29	*Carnobacterium*	0.2	±	0.1	3.2	±	0.5	39.4	6.7
30	*Desemzia*	0.0	±	0.0	0.7	±	0.1	5.0	4.5
31	*Trichococcus*	0.0	±	0.0	1.3	±	0.3	25.2	3.5
32	*Enterococcus*	0.1	±	0.0	1.1	±	0.1	24.6	10.5
33	*Vagococcus*	0.0	±	0.0	2.1	±	0.7	18.6	1.5
34	*Lactobacillus*	0.3	±	0.1	1.0	±	0.1	35.7	25.7
35	*Pediococcus*	0.0	±	0.0	0.9	±	0.2	4.9	1.9
36	Unclassified *Leuconostocaceae*	0.2	±	0.1	2.3	±	0.3	72.5	9.8
37	*Leuconostoc*	0.0	±	0.0	0.1	±	0.0	1.2	4.4
38	*Lactococcus*	0.0	±	0.0	0.9	±	0.2	15.6	4.7
39	*Streptococcus*	0.5	±	0.2	0.8	±	0.0	70.4	63.0
40	*Turicibacter*	0.1	±	0.0	0.3	±	0.0	12.0	17.0
41	Unclassified *Clostridia*	0.0	±	0.0	0.0	±	0.0	0.6	23.0
**42**	**Unclassified *Clostridiales***	15.3	±	0.3	15.3	±	0.6	46.0	100
**43**	**Unclassified *Christensenellaceae***	0.9	±	0.0	1.0	±	0.0	8.2	92.4
44	Unclassified *Clostridiaceae*	0.3	±	0.0	0.3	±	0.0	14.4	90.6
45	02d06 (*Clostridiaceae*)	0.0	±	0.0	0.4	±	0.2	1.0	0.4
**46**	***Clostridium***	0.7	±	0.0	0.7	±	0.0	11.2	98.0
47	*Proteiniclasticum*	0.0	±	0.0	1.2	±	0.4	13.3	1.6
48	*Pseudoramibacter_Eubacterium*	0.0	±	0.0	0.1	±	0.0	0.5	15.8
**49**	**Unclassified *Lachnospiraceae***	6.3	±	0.1	6.3	±	0.2	24.2	100
50	*Anaerostipes*	0.2	±	0.0	0.2	±	0.0	1.6	85.2
51	*Blautia*	0.4	±	0.0	0.4	±	0.0	4.8	95.0
**52**	***Butyrivibrio***	3.4	±	0.1	3.4	±	0.1	18.2	100
**53**	***Coprococcus***	1.1	±	0.0	1.1	±	0.0	5.9	98.4

Ba[a] ID	Group	Mean relative abundance (%, ±SEM[b]) All samples (*n* = 684)[c]	Mean relative abundance (%, ±SEM[b]) Adjusted for prevalence[d]	Maximum (%)[e]	Prevalence (%)[f]
54	*Dorea*	0.0 ± 0.0	0.1 ± 0.0	1.5	57.0
55	*Epulopiscium*	0.0 ± 0.0	0.1 ± 0.0	1.0	2.8
56	*Lachnospira*	0.0 ± 0.0	0.1 ± 0.0	1.7	13.5
57	*Moryella*	0.2 ± 0.0	0.2 ± 0.0	1.1	83.8
**58**	***Pseudobutyrivibrio***	0.9 ± 0.0	0.9 ± 0.0	7.7	94.0
59	*Roseburia*	0.1 ± 0.0	0.1 ± 0.0	2.6	50.7
60	*Shuttleworthia*	0.1 ± 0.0	0.2 ± 0.0	4.2	71.5
61	[*Ruminococcus*] (*Lachnospiraceae*)	0.0 ± 0.0	0.0 ± 0.0	1.5	45.5
62	Unclassified *Peptostreptococcaceae*	0.1 ± 0.1	0.5 ± 0.0	32.3	26.2
63	*Peptostreptococcus*	0.0 ± 0.0	0.3 ± 0.2	0.9	0.4
**64**	**Unclassified *Ruminococcaceae***	7.9 ± 0.2	7.9 ± 0.3	61.1	100
65	*Oscillospira*	0.2 ± 0.0	0.2 ± 0.0	6.4	89.2
**66**	***Ruminococcus***	3.6 ± 0.1	3.6 ± 0.1	30.6	100
**67**	**Unclassified *Veillonellaceae***	1.5 ± 0.2	1.6 ± 0.1	49.1	93.0
68	*Acidaminococcus*	0.0 ± 0.0	0.1 ± 0.0	1.4	12.4
69	*Anaerovibrio*	0.0 ± 0.0	0.1 ± 0.0	0.5	55.0
70	*Dialister*	0.1 ± 0.0	0.5 ± 0.0	6.1	17.1
71	*Megasphaera*	0.0 ± 0.0	0.3 ± 0.0	3.8	8.9
72	*Phascolarctobacterium*	0.0 ± 0.0	0.2 ± 0.0	1.6	2.3
73	*Schwartzia*	0.0 ± 0.0	0.1 ± 0.0	0.7	27.6
74	*Selenomonas*	0.2 ± 0.0	0.3 ± 0.0	4.3	84.1
75	*Sporomusa*	0.0 ± 0.0	0.3 ± 0.0	2.4	7.7
**76**	***Succiniclasticum***	0.7 ± 0.0	0.8 ± 0.0	7.3	97.4
**77**	**Unclassified [*Mogibacteriaceae*]**	0.8 ± 0.0	0.8 ± 0.0	9.2	99.4
**78**	***Mogibacterium***	0.8 ± 0.0	0.8 ± 0.0	12.6	98.8
79	Unclassified [*Tissierellaceae*]	0.0 ± 0.0	0.0 ± 0.0	0.2	1.5
80	*Tissierella-Soehngenia*	0.0 ± 0.0	0.0 ± 0.0	0.2	1.0
81	Unclassified *Erysipelotrichaceae*	0.0 ± 0.0	0.0 ± 0.0	0.5	47.7

Ba[a] ID	Group	Mean relative abundance (%, ±SEM[b])						Maximum (%)[e]	Prevalence (%)[f]
		All samples (n = 684)[c]			Adjusted for prevalence[d]				
82	*Bulleidia*	0.3	±	0.0	0.3	±	0.0	4.5	93.6
83	*Erysipelothrix*	0.0	±	0.0	0.2	±	0.0	16.1	15.5
84	L7A_E11 (*Erysipelotrichaceae*)	0.1	±	0.0	0.1	±	0.0	0.8	74.7
85	RFN20 (*Erysipelotrichaceae*)	0.4	±	0.0	0.4	±	0.0	3.8	93.7
86	*Sharpea*	0.2	±	0.0	0.5	±	0.0	13.2	47.5
87	[*Eubacterium*] (*Erysipelotrichaceae*)	0.0	±	0.0	0.1	±	0.0	2.7	32.5
88	p-75-a5 (*Erysipelotrichaceae*)	0.1	±	0.0	0.1	±	0.0	1.8	81.7
89	*Fusobacterium*	0.0	±	0.0	0.1	±	0.0	0.5	6.0
90	Unclassified *Leptotrichiaceae*	0.0	±	0.0	0.1	±	0.0	0.5	1.6
91	*Leptotrichia*	0.0	±	0.0	0.3	±	0.1	1.1	0.6
92	Unclassified *Victivallaceae*	0.4	±	0.0	0.4	±	0.0	8.5	82.5
93	R4-45B [*Lentisphaeria*]	0.1	±	0.0	0.1	±	0.0	1.9	59.2
94	Unclassified *Pirellulaceae*	0.1	±	0.0	0.1	±	0.0	3.1	70.2
95	Unclassified *Alphaproteobacteria*	0.4	±	0.0	0.5	±	0.0	8.5	87.0
96	RF32 (*Alphaproteobacteria*)	0.3	±	0.1	0.4	±	0.0	37.7	78.8
97	Unclassified *Rickettsiales*	0.1	±	0.0	0.2	±	0.0	9.7	67.5
98	*Sutterella*	0.0	±	0.0	0.1	±	0.0	1.1	33.5
99	*Pelobacter*	0.0	±	0.0	0.1	±	0.0	0.7	8.2
100	0319-6G20 (*Myxococcales*)	0.0	±	0.0	0.1	±	0.0	0.7	30.4
101	*Arcobacter*	0.0	±	0.0	0.1	±	0.0	0.5	0.9
102	*Campylobacter*	0.0	±	0.0	0.0	±	0.0	0.5	25.9
**103**	**Unclassified *Succinivibrionaceae***	1.2	±	0.2	1.7	±	0.1	43.9	72.1
104	*Ruminobacter*	0.3	±	0.1	0.7	±	0.0	47.6	52.2
105	*Succinivibrio*	0.2	±	0.1	0.4	±	0.0	29.5	54.1
106	Unclassified *Enterobacteriaceae*	0.0	±	0.0	0.2	±	0.0	4.4	14.6
107	*Klebsiella*	0.0	±	0.0	0.3	±	0.1	3.7	5.0
108	*Serratia*	0.0	±	0.0	0.1	±	0.0	0.3	1.9
109	Unclassified (SR1)	0.2	±	0.0	0.3	±	0.0	3.3	76.5

Ba[a] ID	Group	Mean relative abundance (%, ±SEM[b])						Maximum (%)[e]	Prevalence (%)[f]
		All samples (*n* = 684)[c]			Adjusted for prevalence[d]				
110	PL-11B10 (*Spirochaetes*)	0.0	±	0.0	0.1	±	0.0	0.6	18.0
111	*Sphaerochaeta*	0.1	±	0.0	0.1	±	0.0	1.7	63.3
112	Unclassified *Spirochaetaceae*	0.0	±	0.0	0.1	±	0.0	0.9	54.5
**113**	***Treponema***	0.6	±	0.0	0.7	±	0.0	4.0	97.2
114	*Pyramidobacter*	0.0	±	0.0	0.1	±	0.0	0.9	61.5
115	TG5 (*Dethiosulfovibrionaceae*)	0.4	±	0.1	0.7	±	0.0	17.6	53.1
**116**	**F16 (TM7)**	0.6	±	0.0	0.7	±	0.0	6.5	96.1
117	Unclassified *Mollicutes*	0.0	±	0.0	0.0	±	0.0	0.6	32.0
118	Unclassified *Anaeroplasmataceae*	0.0	±	0.0	0.1	±	0.0	0.6	39.2
119	*Anaeroplasma*	0.3	±	0.0	0.3	±	0.0	3.4	81.0
120	Unclassified *Mycoplasmataceae*	0.0	±	0.0	0.1	±	0.0	1.7	22.4
**121**	**RF39 (*Mollicutes*)**	0.9	±	0.0	0.9	±	0.0	7.0	99.0
122	ML615J-28 (*Tenericutes*)	0.0	±	0.0	0.1	±	0.0	0.6	46.9
123	Unclassified (*Opitutae*; HA64)	0.0	±	0.0	0.1	±	0.0	1.1	30.7
124	LD1-PB3 (*Verrucomicrobia*)	0.0	±	0.0	0.1	±	0.0	0.6	23.7
125	WCHB1-41 (*Verrucomicrobia*)	0.1	±	0.0	0.1	±	0.0	2.9	63.6
126	Unclassified (WPS-2)	0.1	±	0.0	0.1	±	0.0	0.8	59.9
127	Other groups[i]	0.4	±	0.0	0.4	±	0.0	2.8	99.1
128	Exogenous groups[j]	0.5	±	0.0	0.6	±	0.0	7.9	86.7

Almost all (>99.1%) bacterial sequences were assigned to the above 126 groups of bacteria. [a]Bacteria; [b]standard error of the mean; [c]all samples for which at least 500 bacterial sequencing reads were available for analysis; [d]mean relative abundance in samples that contain that group; [e]the highest relative abundance observed in any one sample; [f]the prevalence of a group in all samples (*n* = 684); [g]indicates the group contains sequences not classified down to the genus level; [h]square brackets indicate suggested but not verified taxon designations; [i]a small proportion of sequences (0.364%) were assigned to a further 556 groups, collectively labelled "other groups", that were not present at >0.5% in even one of the samples; [j]groups (*n* = 66, Supplementary Table 10, representing 0.537% of sequences) that are probably transiently introduced to the rumen *via* the environment.

Supplementary Table 2

Mean relative abundances (%, ± standard error of the mean) of bacterial groups summarised by animal species group. The 30 most abundant bacterial groups are indicated in bold font.

	buffalo	cattle	bison	sheep	goat	deer	giraffes	camelids
Number of samples	24	394	14	98	37	60	11	20
Corynebacterium	0.1 ± 0.0	0.2 ± 0.0	0.0 ± 0.0	0.0 ± 0.0	0.0 ± 0.0	0.0 ± 0.0	0.1 ± 0.0	0.0 ± 0.0
**Unclassified *Coriobacteriaceae***	0.1 ± 0.0	0.5 ± 0.0	0.8 ± 0.2	0.6 ± 0.1	0.4 ± 0.1	0.8 ± 0.1	0.8 ± 0.4	0.2 ± 0.0
Adlercreutzia	0.0 ± 0.0	0.0 ± 0.0	0.0 ± 0.0	0.0 ± 0.0	0.0 ± 0.0	0.0 ± 0.0	0.0 ± 0.0	0.0 ± 0.0
**Unclassified *Bacteroidales***	6.9 ± 0.8	7.5 ± 0.2	9.1 ± 0.7	8.7 ± 0.5	8.2 ± 0.7	10.2 ± 0.8	15.4 ± 2.7	15.0 ± 1.4
**BS11 (*Bacteroidales*)**	2.8 ± 0.6	1.4 ± 0.1	2.3 ± 0.4	1.5 ± 0.3	2.6 ± 0.6	2.0 ± 0.4	0.5 ± 0.2	2.6 ± 0.5
**BF311 (*Bacteroidaceae*)**	0.8 ± 0.1	0.8 ± 0.1	0.5 ± 0.1	0.3 ± 0.0	0.4 ± 0.1	0.3 ± 0.1	0.2 ± 0.1	1.6 ± 0.4
Bacteroides	0.0 ± 0.0	0.0 ± 0.0	0.0 ± 0.0	0.0 ± 0.0	0.0 ± 0.0	0.0 ± 0.0	0.1 ± 0.0	0.1 ± 0.0
Unclassified *Marinilabiaceae*	0.0 ± 0.0	0.0 ± 0.0	0.0 ± 0.0	0.0 ± 0.0	0.0 ± 0.0	0.0 ± 0.0	0.0 ± 0.0	0.0 ± 0.0
Unclassified *Porphyromonadaceae*	0.0 ± 0.0	0.0 ± 0.0	0.0 ± 0.0	0.0 ± 0.0	0.0 ± 0.0	0.0 ± 0.0	0.0 ± 0.0	0.0 ± 0.0
Dysgonomonas	0.0 ± 0.0	0.0 ± 0.0	0.0 ± 0.0	0.0 ± 0.0	0.0 ± 0.0	0.0 ± 0.0	0.0 ± 0.0	0.0 ± 0.0
Paludibacter	0.2 ± 0.0	0.1 ± 0.0	0.1 ± 0.0	0.1 ± 0.0	0.2 ± 0.1	0.1 ± 0.0	0.2 ± 0.1	0.0 ± 0.0
Parabacteroides	0.0 ± 0.0	0.0 ± 0.0	0.0 ± 0.0	0.0 ± 0.0	0.0 ± 0.0	0.0 ± 0.0	0.0 ± 0.0	0.0 ± 0.0
Unclassified *Prevotellaceae*	0.3 ± 0.1	0.3 ± 0.0	0.2 ± 0.0	0.3 ± 0.0	0.2 ± 0.0	0.3 ± 0.1	0.2 ± 0.1	0.3 ± 0.1
Prevotella	25.8 ± 2.8	21.4 ± 0.8	15.1 ± 1.3	21.5 ± 1.8	30.8 ± 2.2	22.0 ± 1.1	28.4 ± 4.1	19.8 ± 2.0
**RF16 (*Bacteroidales*)**	7.1 ± 0.8	1.1 ± 0.1	0.9 ± 0.2	0.9 ± 0.2	1.4 ± 0.2	1.5 ± 0.2	1.7 ± 0.5	1.1 ± 0.3
**S24-7 (*Bacteroidales*)**	0.9 ± 0.2	1.7 ± 0.1	2.5 ± 0.8	1.7 ± 0.2	2.3 ± 0.6	1.3 ± 0.2	1.6 ± 0.6	0.7 ± 0.1
**Unclassified [*Paraprevotellaceae*]**	0.6 ± 0.0	0.6 ± 0.0	0.8 ± 0.1	0.8 ± 0.1	0.7 ± 0.1	1.9 ± 0.4	1.0 ± 0.4	1.3 ± 0.2
**CF231 [*Paraprevotellaceae*]**	1.1 ± 0.1	0.7 ± 0.0	0.9 ± 0.2	0.5 ± 0.0	1.1 ± 0.2	1.4 ± 0.2	1.9 ± 0.3	1.6 ± 0.3
**YRC22 [*Paraprevotellaceae*]**	0.9 ± 0.1	1.0 ± 0.1	1.0 ± 0.1	0.7 ± 0.1	0.7 ± 0.1	2.0 ± 0.2	2.6 ± 0.5	0.9 ± 0.2
[*Prevotella*] [*Paraprevotellaceae*]	0.1 ± 0.0	0.0 ± 0.0	0.0 ± 0.0	0.1 ± 0.0	0.1 ± 0.0	0.1 ± 0.0	0.2 ± 0.1	0.1 ± 0.0
p-2534-18B5 (*Bacteroidales*)	0.0 ± 0.0	0.1 ± 0.0	0.0 ± 0.0	0.1 ± 0.1	0.0 ± 0.0	0.0 ± 0.0	0.3 ± 0.3	0.0 ± 0.0
**SHD-231 (*Anaerolinaceae*)**	0.3 ± 0.1	0.5 ± 0.0	0.7 ± 0.2	0.6 ± 0.1	1.5 ± 0.6	0.6 ± 0.2	0.5 ± 0.2	0.1 ± 0.0

	buffalo	cattle	bison	sheep	goat	deer	giraffes	camelids
**YS2 (*Cyanobacteria*)**	2.5 ± 0.5	0.7 ± 0.1	0.2 ± 0.1	0.2 ± 0.0	0.4 ± 0.1	0.6 ± 0.1	1.0 ± 0.5	0.6 ± 0.2
Unclassified *Elusimicrobiaceae*	0.0 ± 0.0	0.0 ± 0.0	0.0 ± 0.0	0.0 ± 0.0	0.0 ± 0.0	0.0 ± 0.0	0.0 ± 0.0	0.0 ± 0.0
Unclassified *Endomicrobia*	0.0 ± 0.0	0.0 ± 0.0	0.0 ± 0.0	0.0 ± 0.0	0.0 ± 0.0	0.0 ± 0.0	0.0 ± 0.0	0.0 ± 0.0
Fibrobacter	4.4 ± 0.6	3.8 ± 0.2	2.5 ± 0.5	1.9 ± 0.3	1.6 ± 0.3	0.9 ± 0.1	0.7 ± 0.2	0.6 ± 0.1
Unclassified *Lactobacillales*	0.0 ± 0.0	0.0 ± 0.0	0.0 ± 0.0	0.0 ± 0.0	0.0 ± 0.0	0.0 ± 0.0	0.0 ± 0.0	0.0 ± 0.0
Aerococcus	0.0 ± 0.0	0.0 ± 0.0	0.0 ± 0.0	0.0 ± 0.0	0.0 ± 0.0	0.0 ± 0.0	0.0 ± 0.0	0.0 ± 0.0
Carnobacterium	0.0 ± 0.0	0.3 ± 0.1	0.0 ± 0.0	0.4 ± 0.3	0.0 ± 0.0	0.0 ± 0.0	0.0 ± 0.0	0.0 ± 0.0
Desemzia	0.0 ± 0.0	0.0 ± 0.0	0.0 ± 0.0	0.2 ± 0.1	0.0 ± 0.0	0.0 ± 0.0	0.0 ± 0.0	0.0 ± 0.0
Trichococcus	0.0 ± 0.0	0.1 ± 0.1	0.0 ± 0.0	0.1 ± 0.0	0.0 ± 0.0	0.0 ± 0.0	0.0 ± 0.0	0.0 ± 0.0
Enterococcus	0.0 ± 0.0	0.0 ± 0.0	0.0 ± 0.0	0.6 ± 0.3	0.0 ± 0.0	0.0 ± 0.0	0.0 ± 0.0	0.0 ± 0.0
Vagococcus	0.0 ± 0.0	0.0 ± 0.0	0.0 ± 0.0	0.2 ± 0.2	0.0 ± 0.0	0.0 ± 0.0	0.0 ± 0.0	0.0 ± 0.0
Lactobacillus	0.1 ± 0.0	0.2 ± 0.1	0.0 ± 0.0	0.3 ± 0.3	2.0 ± 1.3	0.0 ± 0.0	0.0 ± 0.0	0.0 ± 0.0
Pediococcus	0.0 ± 0.0	0.0 ± 0.0	0.0 ± 0.0	0.1 ± 0.1	0.1 ± 0.1	0.0 ± 0.0	0.0 ± 0.0	0.0 ± 0.0
Unclassified *Leuconostocaceae*	0.1 ± 0.0	0.0 ± 0.0	0.0 ± 0.0	1.2 ± 0.8	0.6 ± 0.6	0.0 ± 0.0	0.0 ± 0.0	0.0 ± 0.0
Leuconostoc	0.0 ± 0.0	0.0 ± 0.0	0.0 ± 0.0	0.0 ± 0.0	0.0 ± 0.0	0.0 ± 0.0	0.0 ± 0.0	0.0 ± 0.0
Lactococcus	0.0 ± 0.0	0.0 ± 0.0	0.0 ± 0.0	0.1 ± 0.1	0.0 ± 0.0	0.0 ± 0.0	0.0 ± 0.0	0.0 ± 0.0
Streptococcus	0.1 ± 0.0	0.5 ± 0.2	0.1 ± 0.0	0.4 ± 0.2	2.2 ± 0.9	0.1 ± 0.0	0.1 ± 0.0	0.0 ± 0.0
Turicibacter	0.5 ± 0.5	0.1 ± 0.0	0.0 ± 0.0	0.0 ± 0.0	0.0 ± 0.0	0.0 ± 0.0	0.1 ± 0.0	0.0 ± 0.0
Unclassified *Clostridia*	0.0 ± 0.0	0.0 ± 0.0	0.0 ± 0.0	0.0 ± 0.0	0.0 ± 0.0	0.0 ± 0.0	0.0 ± 0.0	0.0 ± 0.0
**Unclassified *Clostridiales***	9.8 ± 0.8	16.3 ± 0.4	18.5 ± 1.3	16.5 ± 1.0	11.5 ± 0.8	15.2 ± 0.9	6.8 ± 0.9	13.8 ± 0.9
**Unclassified *Christensenellaceae***	1.4 ± 0.5	1.0 ± 0.1	0.9 ± 0.1	1.1 ± 0.1	0.5 ± 0.1	0.5 ± 0.1	0.3 ± 0.2	1.6 ± 0.5
Unclassified *Clostridiaceae*	0.9 ± 0.6	0.3 ± 0.0	0.2 ± 0.0	0.4 ± 0.0	0.1 ± 0.0	0.1 ± 0.0	0.2 ± 0.1	0.2 ± 0.0
02d06 (*Clostridiaceae*)	0.0 ± 0.0	0.0 ± 0.0	0.0 ± 0.0	0.0 ± 0.0	0.0 ± 0.0	0.0 ± 0.0	0.0 ± 0.0	0.0 ± 0.0
Clostridium	0.7 ± 0.1	0.8 ± 0.0	0.5 ± 0.1	0.7 ± 0.1	0.4 ± 0.1	0.5 ± 0.1	0.2 ± 0.0	0.7 ± 0.1
Proteiniclasticum	0.0 ± 0.0	0.0 ± 0.0	0.0 ± 0.0	0.0 ± 0.0	0.0 ± 0.0	0.0 ± 0.0	0.0 ± 0.0	0.0 ± 0.0
Pseudoramibacter_Eubacterium	0.0 ± 0.0	0.0 ± 0.0	0.0 ± 0.0	0.0 ± 0.0	0.0 ± 0.0	0.0 ± 0.0	0.0 ± 0.0	0.0 ± 0.0

	buffalo	cattle	bison	sheep	goat	deer	giraffes	camelids
**Unclassified *Lachnospiraceae***	3.0 ± 0.3	6.7 ± 0.2	7.7 ± 0.4	6.0 ± 0.4	4.4 ± 0.4	6.9 ± 0.4	4.3 ± 0.8	5.8 ± 0.5
Anaerostipes	0.2 ± 0.0	0.2 ± 0.0	0.3 ± 0.1	0.2 ± 0.0	0.1 ± 0.0	0.1 ± 0.0	0.0 ± 0.0	0.1 ± 0.0
Blautia	0.1 ± 0.0	0.4 ± 0.0	0.8 ± 0.1	0.4 ± 0.1	0.3 ± 0.1	0.5 ± 0.1	0.2 ± 0.1	0.3 ± 0.1
Butyrivibrio	1.3 ± 0.2	4.1 ± 0.1	4.9 ± 0.5	3.3 ± 0.3	2.1 ± 0.3	1.7 ± 0.2	1.5 ± 0.2	3.4 ± 0.7
Coprococcus	0.7 ± 0.1	1.2 ± 0.0	1.0 ± 0.1	0.8 ± 0.1	0.9 ± 0.2	1.4 ± 0.1	0.5 ± 0.1	0.9 ± 0.1
Dorea	0.0 ± 0.0	0.0 ± 0.0	0.0 ± 0.0	0.0 ± 0.0	0.0 ± 0.0	0.1 ± 0.0	0.0 ± 0.0	0.1 ± 0.0
Epulopiscium	0.0 ± 0.0	0.0 ± 0.0	0.0 ± 0.0	0.0 ± 0.0	0.0 ± 0.0	0.0 ± 0.0	0.0 ± 0.0	0.0 ± 0.0
Lachnospira	0.0 ± 0.0	0.0 ± 0.0	0.0 ± 0.0	0.0 ± 0.0	0.0 ± 0.0	0.0 ± 0.0	0.0 ± 0.0	0.0 ± 0.0
Moryella	0.0 ± 0.0	0.2 ± 0.0	0.3 ± 0.1	0.1 ± 0.0	0.2 ± 0.0	0.1 ± 0.0	0.1 ± 0.0	0.1 ± 0.0
Pseudobutyrivibrio	1.0 ± 0.1	1.1 ± 0.1	1.0 ± 0.2	0.6 ± 0.1	0.8 ± 0.2	0.5 ± 0.1	0.3 ± 0.1	0.7 ± 0.2
Roseburia	0.0 ± 0.0	0.0 ± 0.0	0.0 ± 0.0	0.0 ± 0.0	0.0 ± 0.0	0.1 ± 0.0	0.1 ± 0.1	0.1 ± 0.0
Shuttleworthia	0.1 ± 0.0	0.2 ± 0.0	0.2 ± 0.1	0.1 ± 0.0	0.1 ± 0.0	0.0 ± 0.0	0.0 ± 0.0	0.2 ± 0.0
[*Ruminococcus*] (*Lachnospiraceae*)	0.0 ± 0.0	0.0 ± 0.0	0.0 ± 0.0	0.0 ± 0.0	0.0 ± 0.0	0.0 ± 0.0	0.0 ± 0.0	0.0 ± 0.0
Unclassified *Peptostreptococcaceae*	1.5 ± 1.3	0.1 ± 0.1	0.0 ± 0.0	0.0 ± 0.0	0.0 ± 0.0	0.0 ± 0.0	0.1 ± 0.1	0.0 ± 0.0
Peptostreptococcus	0.0 ± 0.0	0.0 ± 0.0	0.0 ± 0.0	0.0 ± 0.0	0.0 ± 0.0	0.0 ± 0.0	0.0 ± 0.0	0.0 ± 0.0
**Unclassified *Ruminococcaceae***	4.6 ± 0.7	7.8 ± 0.2	8.6 ± 0.4	10.0 ± 0.9	5.3 ± 0.4	9.1 ± 0.4	9.8 ± 2.8	8.2 ± 0.8
Oscillospira	0.1 ± 0.0	0.1 ± 0.0	0.2 ± 0.0	0.2 ± 0.0	0.1 ± 0.0	0.7 ± 0.2	0.3 ± 0.1	0.4 ± 0.1
Ruminococcus	2.0 ± 0.2	3.9 ± 0.1	5.7 ± 1.1	3.5 ± 0.5	3.4 ± 0.7	3.6 ± 0.4	2.4 ± 0.7	3.0 ± 0.4
**Unclassified *Veillonellaceae***	0.1 ± 0.0	0.3 ± 0.1	0.3 ± 0.1	2.5 ± 0.7	1.6 ± 0.4	4.5 ± 0.8	2.4 ± 1.6	5.5 ± 0.9
Acidaminococcus	0.0 ± 0.0	0.0 ± 0.0	0.0 ± 0.0	0.0 ± 0.0	0.0 ± 0.0	0.0 ± 0.0	0.0 ± 0.0	0.0 ± 0.0
Anaerovibrio	0.1 ± 0.0	0.0 ± 0.0	0.1 ± 0.0	0.0 ± 0.0	0.0 ± 0.0	0.0 ± 0.0	0.1 ± 0.0	0.0 ± 0.0
Dialister	0.0 ± 0.0	0.1 ± 0.0	0.0 ± 0.0	0.2 ± 0.1	0.0 ± 0.0	0.0 ± 0.0	0.0 ± 0.0	0.0 ± 0.0
Megasphaera	0.0 ± 0.0	0.0 ± 0.0	0.0 ± 0.0	0.0 ± 0.0	0.0 ± 0.0	0.0 ± 0.0	0.0 ± 0.0	0.0 ± 0.0
Phascolarctobacterium	0.0 ± 0.0	0.0 ± 0.0	0.0 ± 0.0	0.0 ± 0.0	0.0 ± 0.0	0.0 ± 0.0	0.0 ± 0.0	0.0 ± 0.0
Schwartzia	0.0 ± 0.0	0.0 ± 0.0	0.0 ± 0.0	0.0 ± 0.0	0.0 ± 0.0	0.0 ± 0.0	0.0 ± 0.0	0.1 ± 0.0
Selenomonas	0.1 ± 0.0	0.2 ± 0.0	0.3 ± 0.1	0.2 ± 0.1	0.3 ± 0.1	0.3 ± 0.0	0.4 ± 0.2	0.2 ± 0.0

	buffalo	cattle	bison	sheep	goat	deer	giraffes	camelids
Sporomusa	0.0 ± 0.0	0.0 ± 0.0	0.0 ± 0.0	0.0 ± 0.0	0.0 ± 0.0	0.1 ± 0.1	0.0 ± 0.0	0.1 ± 0.0
Succiniclasticum	0.4 ± 0.1	0.8 ± 0.0	1.6 ± 0.3	0.6 ± 0.1	0.6 ± 0.1	0.4 ± 0.0	1.0 ± 0.2	0.5 ± 0.1
**Unclassified [*Mogibacteriaceae*]**	0.6 ± 0.2	0.8 ± 0.0	0.7 ± 0.1	1.3 ± 0.1	0.4 ± 0.0	0.7 ± 0.1	0.6 ± 0.2	0.7 ± 0.1
Mogibacterium	0.6 ± 0.2	0.8 ± 0.0	0.9 ± 0.1	1.1 ± 0.1	0.5 ± 0.1	0.6 ± 0.1	0.2 ± 0.0	0.4 ± 0.1
Unclassified [*Tissierellaceae*]	0.0 ± 0.0	0.0 ± 0.0	0.0 ± 0.0	0.0 ± 0.0	0.0 ± 0.0	0.0 ± 0.0	0.0 ± 0.0	0.0 ± 0.0
Tissierella-Soehngenia	0.0 ± 0.0	0.0 ± 0.0	0.0 ± 0.0	0.0 ± 0.0	0.0 ± 0.0	0.0 ± 0.0	0.0 ± 0.0	0.0 ± 0.0
Unclassified *Erysipelotrichaceae*	0.0 ± 0.0	0.0 ± 0.0	0.0 ± 0.0	0.0 ± 0.0	0.0 ± 0.0	0.0 ± 0.0	0.0 ± 0.0	0.0 ± 0.0
Bulleidia	0.1 ± 0.0	0.3 ± 0.0	0.3 ± 0.1	0.4 ± 0.0	0.2 ± 0.0	0.2 ± 0.0	0.1 ± 0.0	0.2 ± 0.1
Erysipelothrix	0.0 ± 0.0	0.0 ± 0.0	0.0 ± 0.0	0.0 ± 0.0	0.0 ± 0.0	0.0 ± 0.0	0.0 ± 0.0	0.0 ± 0.0
L7A_E11 (*Erysipelotrichaceae*)	0.0 ± 0.0	0.1 ± 0.0	0.1 ± 0.0	0.1 ± 0.0	0.0 ± 0.0	0.1 ± 0.0	0.1 ± 0.1	0.1 ± 0.0
RFN20 *(Erysipelotrichaceae*)	0.8 ± 0.1	0.4 ± 0.0	0.1 ± 0.0	0.3 ± 0.0	0.3 ± 0.0	0.1 ± 0.0	0.4 ± 0.2	0.4 ± 0.1
Sharpea	0.0 ± 0.0	0.2 ± 0.0	0.0 ± 0.0	0.5 ± 0.2	0.0 ± 0.0	0.2 ± 0.2	0.0 ± 0.0	0.1 ± 0.1
[*Eubacterium*] (*Erysipelotrichaceae*)	0.0 ± 0.0	0.0 ± 0.0	0.0 ± 0.0	0.1 ± 0.0	0.0 ± 0.0	0.0 ± 0.0	0.0 ± 0.0	0.0 ± 0.0
p-75-a5 (*Erysipelotrichaceae*)	0.0 ± 0.0	0.1 ± 0.0	0.1 ± 0.0	0.1 ± 0.0	0.1 ± 0.0	0.1 ± 0.0	0.1 ± 0.0	0.1 ± 0.0
Fusobacterium	0.0 ± 0.0	0.0 ± 0.0	0.0 ± 0.0	0.0 ± 0.0	0.0 ± 0.0	0.0 ± 0.0	0.0 ± 0.0	0.0 ± 0.0
Unclassified *Leptotrichiaceae*	0.0 ± 0.0	0.0 ± 0.0	0.0 ± 0.0	0.0 ± 0.0	0.0 ± 0.0	0.0 ± 0.0	0.0 ± 0.0	0.0 ± 0.0
Leptotrichia	0.0 ± 0.0	0.0 ± 0.0	0.0 ± 0.0	0.0 ± 0.0	0.0 ± 0.0	0.0 ± 0.0	0.0 ± 0.0	0.0 ± 0.0
Unclassified *Victivallaceae*	0.6 ± 0.1	0.4 ± 0.0	0.1 ± 0.0	0.2 ± 0.0	0.4 ± 0.1	0.2 ± 0.0	0.3 ± 0.1	0.2 ± 0.1
R4-45B [*Lentisphaeria*]	0.1 ± 0.0	0.1 ± 0.0	0.1 ± 0.0	0.0 ± 0.0	0.0 ± 0.0	0.0 ± 0.0	0.1 ± 0.1	0.0 ± 0.0
Unclassified *Pirellulaceae*	0.1 ± 0.0	0.1 ± 0.0	0.1 ± 0.0	0.1 ± 0.0	0.3 ± 0.1	0.1 ± 0.0	0.0 ± 0.0	0.0 ± 0.0
Unclassified *Alphaproteobacteria*	1.8 ± 0.3	0.4 ± 0.0	0.2 ± 0.0	0.2 ± 0.0	0.3 ± 0.1	0.3 ± 0.1	0.2 ± 0.1	0.2 ± 0.1
RF32 (*Alphaproteobacteria*)	0.5 ± 0.1	0.2 ± 0.0	0.1 ± 0.0	0.1 ± 0.0	0.3 ± 0.1	0.1 ± 0.0	0.3 ± 0.1	0.1 ± 0.0
Unclassified *Rickettsiales*	0.6 ± 0.2	0.1 ± 0.0	0.1 ± 0.0	0.0 ± 0.0	0.1 ± 0.0	0.1 ± 0.0	1.0 ± 0.8	0.0 ± 0.0
Sutterella	0.0 ± 0.0	0.0 ± 0.0	0.0 ± 0.0	0.0 ± 0.0	0.0 ± 0.0	0.0 ± 0.0	0.0 ± 0.0	0.0 ± 0.0
Pelobacter	0.0 ± 0.0	0.0 ± 0.0	0.0 ± 0.0	0.0 ± 0.0	0.0 ± 0.0	0.0 ± 0.0	0.0 ± 0.0	0.0 ± 0.0
0319-6G20 (*Myxococcales*)	0.0 ± 0.0	0.0 ± 0.0	0.0 ± 0.0	0.0 ± 0.0	0.0 ± 0.0	0.0 ± 0.0	0.0 ± 0.0	0.0 ± 0.0

	buffalo	cattle	bison	sheep	goat	deer	giraffes	camelids
Arcobacter	0.0 ± 0.0	0.0 ± 0.0	0.0 ± 0.0	0.0 ± 0.0	0.0 ± 0.0	0.0 ± 0.0	0.0 ± 0.0	0.0 ± 0.0
Campylobacter	0.1 ± 0.0	0.0 ± 0.0	0.0 ± 0.0	0.0 ± 0.0	0.0 ± 0.0	0.0 ± 0.0	0.0 ± 0.0	0.0 ± 0.0
**Unclassified *Succinivibrionaceae***	1.7 ± 0.5	1.6 ± 0.2	2.4 ± 1.2	0.2 ± 0.1	2.3 ± 0.8	0.1 ± 0.0	0.1 ± 0.0	0.0 ± 0.0
Ruminobacter	1.3 ± 0.4	0.2 ± 0.0	0.4 ± 0.2	0.1 ± 0.1	1.1 ± 0.6	0.0 ± 0.0	1.8 ± 1.6	0.0 ± 0.0
Succinivibrio	0.2 ± 0.0	0.3 ± 0.1	0.1 ± 0.0	0.2 ± 0.1	0.3 ± 0.1	0.0 ± 0.0	0.0 ± 0.0	0.0 ± 0.0
Unclassified *Enterobacteriaceae*	0.4 ± 0.2	0.0 ± 0.0	0.0 ± 0.0	0.1 ± 0.0	0.0 ± 0.0	0.0 ± 0.0	0.0 ± 0.0	0.0 ± 0.0
Klebsiella	0.4 ± 0.2	0.0 ± 0.0	0.0 ± 0.0	0.0 ± 0.0	0.0 ± 0.0	0.0 ± 0.0	0.0 ± 0.0	0.0 ± 0.0
Serratia	0.0 ± 0.0	0.0 ± 0.0	0.0 ± 0.0	0.0 ± 0.0	0.0 ± 0.0	0.0 ± 0.0	0.0 ± 0.0	0.0 ± 0.0
Unclassified (SR1)	0.3 ± 0.0	0.3 ± 0.0	0.1 ± 0.0	0.1 ± 0.0	0.1 ± 0.0	0.2 ± 0.0	0.4 ± 0.3	0.2 ± 0.1
PL-11B10 (*Spirochaetes*)	0.0 ± 0.0	0.0 ± 0.0	0.0 ± 0.0	0.0 ± 0.0	0.0 ± 0.0	0.0 ± 0.0	0.0 ± 0.0	0.0 ± 0.0
Sphaerochaeta	0.1 ± 0.0	0.1 ± 0.0	0.0 ± 0.0	0.0 ± 0.0	0.1 ± 0.1	0.0 ± 0.0	0.2 ± 0.1	0.0 ± 0.0
Unclassified *Spirochaetaceae*	0.0 ± 0.0	0.0 ± 0.0	0.0 ± 0.0	0.0 ± 0.0	0.0 ± 0.0	0.0 ± 0.0	0.0 ± 0.0	0.0 ± 0.0
Treponema	0.3 ± 0.0	0.7 ± 0.0	0.7 ± 0.1	0.4 ± 0.1	0.6 ± 0.1	0.5 ± 0.1	1.0 ± 0.4	0.5 ± 0.1
Pyramidobacter	0.0 ± 0.0	0.0 ± 0.0	0.0 ± 0.0	0.0 ± 0.0	0.0 ± 0.0	0.0 ± 0.0	0.1 ± 0.0	0.0 ± 0.0
TG5 (*Dethiosulfovibrionaceae*)	0.0 ± 0.0	0.0 ± 0.0	0.0 ± 0.0	0.3 ± 0.1	0.7 ± 0.2	0.9 ± 0.2	0.4 ± 0.2	1.6 ± 0.4
**F16 (TM7)**	1.1 ± 0.1	0.7 ± 0.0	0.3 ± 0.0	0.5 ± 0.1	0.3 ± 0.1	0.4 ± 0.0	0.5 ± 0.1	0.6 ± 0.1
Unclassified *Mollicutes*	0.0 ± 0.0	0.0 ± 0.0	0.0 ± 0.0	0.0 ± 0.0	0.0 ± 0.0	0.0 ± 0.0	0.0 ± 0.0	0.0 ± 0.0
Unclassified *Anaeroplasmataceae*	0.1 ± 0.0	0.0 ± 0.0	0.0 ± 0.0	0.0 ± 0.0	0.0 ± 0.0	0.0 ± 0.0	0.0 ± 0.0	0.1 ± 0.0
Anaeroplasma	0.8 ± 0.1	0.3 ± 0.0	0.1 ± 0.0	0.2 ± 0.0	0.3 ± 0.1	0.1 ± 0.0	0.5 ± 0.3	0.4 ± 0.1
Unclassified *Mycoplasmataceae*	0.3 ± 0.1	0.0 ± 0.0	0.0 ± 0.0	0.0 ± 0.0	0.0 ± 0.0	0.0 ± 0.0	0.0 ± 0.0	0.0 ± 0.0
**RF39 (*Mollicutes*)**	1.5 ± 0.2	1.1 ± 0.0	0.7 ± 0.1	0.7 ± 0.1	0.4 ± 0.1	0.8 ± 0.1	0.3 ± 0.1	1.0 ± 0.1
ML615J-28 (*Tenericutes*)	0.0 ± 0.0	0.0 ± 0.0	0.0 ± 0.0	0.0 ± 0.0	0.0 ± 0.0	0.0 ± 0.0	0.0 ± 0.0	0.1 ± 0.0
Unclassified (*Opitutae*; HA64)	0.1 ± 0.0	0.0 ± 0.0	0.0 ± 0.0	0.0 ± 0.0	0.0 ± 0.0	0.0 ± 0.0	0.2 ± 0.1	0.0 ± 0.0
LD1-PB3 (*Verrucomicrobia*)	0.0 ± 0.0	0.0 ± 0.0	0.0 ± 0.0	0.0 ± 0.0	0.0 ± 0.0	0.0 ± 0.0	0.0 ± 0.0	0.0 ± 0.0
WCHB1-41 (*Verrucomicrobia*)	0.2 ± 0.0	0.1 ± 0.0	0.0 ± 0.0	0.0 ± 0.0	0.1 ± 0.0	0.0 ± 0.0	0.3 ± 0.2	0.0 ± 0.0
Unclassified (WPS-2)	0.0 ± 0.0	0.1 ± 0.0	0.0 ± 0.0	0.1 ± 0.0	0.1 ± 0.0	0.0 ± 0.0	0.0 ± 0.0	0.1 ± 0.0

Supplementary Table 3
The most abundant and prevalent rumen archaeal (Ar) species-level groups. The 5 most abundant groups are indicated in bold font.

Ar[a] ID	Group	Mean relative abundance (%, ±SEM[b]) All samples (n = 704)[c]			Mean relative abundance (%, ±SEM[b]) Adjusted for prevalence[d]			Maximum (%)[e]	Prevalence (%)[f]
1	No BLAST hit	0.0	±	0.0	0.1	±	0.0	0.8	4.4
2	*Methanobacterium formicicum*	0.0	±	0.0	0.2	±	0.1	1.1	0.9
3	*Methanobacterium palustre*	0.0	±	0.0	0.2	±	0.1	0.6	1.0
4	*Methanobacterium* sp.	0.6	±	0.1	1.3	±	0.1	13.2	44.7
5	*Methanobrevibacter acididurans*	0.0	±	0.0	0.6	±	0.1	2.9	8.2
6	*Methanobrevibacter boviskoreani*	0.9	±	0.2	15.9	±	2.5	86.6	5.7
7	***Methanobrevibacter gottschalkii* clade**	46.9	±	0.8	46.9	±	1.8	99.5	100
8	***Methanobrevibacter ruminantium* clade**	27.1	±	0.8	27.3	±	1.0	91.2	99.1
9	*Methanobrevibacter smithii*	0.5	±	0.0	0.6	±	0.0	20.4	77.0
10	*Methanobrevibacter* sp. RT	0.0	±	0.0	0.2	±	0.1	0.5	0.6
11	*Methanobrevibacter wolinii*	1.0	±	0.2	2.5	±	0.1	69.4	40.6
12	*Methanosphaera cuniculi*	0.2	±	0.0	0.7	±	0.1	7.3	27.4
13	*Methanosphaera* sp. Group 5	2.1	±	0.2	3.0	±	0.1	72.9	71.3
14	*Methanosphaera* sp. A4	0.1	±	0.0	1.4	±	0.2	7.2	5.1
15	***Methanosphaera* sp. ISO3-F5**	5.7	±	0.3	5.8	±	0.2	73.7	97.4
16	*Methanocorpusculum sinense*	0.0	±	0.0	0.3	±	0.1	1.2	0.9
17	*Methanoculleus* sp.	0.0	±	0.0	0.3	±	0.2	0.6	0.4
18	*Methanomicrobium mobile*	0.7	±	0.2	3.4	±	0.3	75.9	21.6
19	*Methanosaeta concilii*	0.0	±	0.0	0.4	±	0.3	1.2	0.4
20	*Methanimicrococcus blatticola*	0.6	±	0.1	2.1	±	0.1	39.0	30.7
21	*Methanosarcina horonobensis*	0.0	±	0.0	0.2	±	0.1	0.7	0.7
22	*Methanosarcina mazei*	0.0	±	0.0	0.6	±	0.2	4.4	2.0
23	***Mmc.*[g]; Group 10 sp.**	3.0	±	0.2	3.6	±	0.1	85.8	84.8
24	*Mmc*; *Candidatus* Methanomethylophilus alvus	0.8	±	0.2	6.3	±	0.7	84.6	13.2

Ar[a] ID	Group	Mean relative abundance (%, ±SEM[b])		Maximum (%)[e]	Prevalence (%)[f]
		All samples (n = 704)[c]	Adjusted for prevalence[d]		
25	*Mmc*; Group 11 sp. BRNA1	0.0 ± 0.0	0.2 ± 0.0	1.6	3.0
26	*Mmc.*; Group 11 sp. CRM1	0.2 ± 0.1	0.6 ± 0.0	32.6	26.4
27	*Mmc.*; Group 11 sp. ISO4-G11	0.2 ± 0.0	0.5 ± 0.0	12.3	43.5
28	*Mmc.*; Group 11 sp. from human dental plaque	0.0 ± 0.0	0.3 ± 0.1	1.8	1.7
29	***Mmc.*; Group 12 sp. ISO4-H5**	6.5 ± 0.4	7.4 ± 0.3	67.9	87.1
30	*Mmc.*; Group 3b sp.	0.1 ± 0.0	0.3 ± 0.0	1.9	26.0
31	*Mmc.*; Group 4 sp. MpT1	1.0 ± 0.1	1.4 ± 0.1	12.8	70.5
32	*Mmc.*; Group 8 sp.	0.4 ± 0.0	0.8 ± 0.0	14.0	51.8
33	*Mmc.*; Group 9 sp.	1.2 ± 0.1	1.7 ± 0.1	38.0	72.4
34	Other groups[h]	0.0 ± 0.0	0.1 ± 0.0	0.9	63.2

[a]Archaea; [b]standard error of the mean; [c]all samples for which at least 100 archaea sequencing reads were available for analysis; [d]mean relative abundance in samples that contain that group; [e]the highest relative abundance observed in any one sample; [f]the prevalence of a group in all samples (n = 704); [g]*Mmc.*, *Methanomassiliicoccaceae*; [h]relative abundance <0.5% in any one sample.

Supplementary Table 4

Mean relative abundances (%, ± standard error of the mean) of archaeal groups summarised by animal species group. The five most archaeal abundant groups are indicated in bold font.

	buffalo	cattle	bison	sheep	goat	deer	giraffes	camelids
Number of samples	26	396	14	106	52	59	12	20
Methanobacterium formicicum	0.0 ± 0.0	0.0 ± 0.0	0.0 ± 0.0	0.0 ± 0.0	0.0 ± 0.0	0.0 ± 0.0	0.0 ± 0.0	0.0 ± 0.0
Methanobacterium palustre	0.0 ± 0.0	0.0 ± 0.0	0.0 ± 0.0	0.0 ± 0.0	0.0 ± 0.0	0.0 ± 0.0	0.0 ± 0.0	0.0 ± 0.0
Methanobacterium sp.	0.3 ± 0.1	0.7 ± 0.1	0.5 ± 0.2	0.4 ± 0.1	0.3 ± 0.1	0.1 ± 0.0	0.0 ± 0.0	1.3 ± 0.6
Methanobrevibacter acididurans	0.0 ± 0.0	0.0 ± 0.0	0.0 ± 0.0	0.1 ± 0.0	0.2 ± 0.1	0.2 ± 0.1	0.2 ± 0.2	0.1 ± 0.1
Methanobrevibacter boviskoreani	0.0 ± 0.0	1.5 ± 0.4	0.0 ± 0.0	0.6 ± 0.4	0.0 ± 0.0	0.0 ± 0.0	0.0 ± 0.0	0.0 ± 0.0
***Methanobrevibacter gottschalkii* clade**	55.7 ± 3.2	42.6 ± 1.0	52.9 ± 5.9	54.5 ± 2.1	57.2 ± 3.6	52.7 ± 3.3	44.5 ± 7.7	24.9 ± 4.5
***Methanobrevibacter ruminantium* clade**	14.4 ± 2.5	31.6 ± 0.9	30.4 ± 6.3	25.7 ± 2.0	18.3 ± 2.7	17.0 ± 2.7	21.8 ± 5.3	37.3 ± 5.0
Methanobrevibacter smithii	1.1 ± 0.2	0.3 ± 0.0	0.4 ± 0.1	0.4 ± 0.0	0.3 ± 0.1	0.3 ± 0.1	0.5 ± 0.2	3.5 ± 1.2
Methanobrevibacter sp. RT	0.0 ± 0.0	0.0 ± 0.0	0.0 ± 0.0	0.0 ± 0.0	0.0 ± 0.0	0.0 ± 0.0	0.0 ± 0.0	0.0 ± 0.0
Methanobrevibacter wolinii	2.4 ± 0.6	0.5 ± 0.2	0.6 ± 0.4	1.5 ± 0.7	3.6 ± 1.3	0.2 ± 0.1	1.7 ± 0.9	2.8 ± 1.8
Methanosphaera cuniculi	0.1 ± 0.1	0.1 ± 0.0	0.2 ± 0.1	0.3 ± 0.1	0.1 ± 0.1	0.3 ± 0.1	0.2 ± 0.2	0.1 ± 0.0
Methanosphaera sp. Group 5	0.4 ± 0.2	1.3 ± 0.3	1.6 ± 0.4	1.8 ± 0.2	1.5 ± 0.5	7.6 ± 1.3	3.2 ± 1.4	4.5 ± 1.7
Methanosphaera sp. A4	0.0 ± 0.0	0.1 ± 0.0	0.0 ± 0.0	0.1 ± 0.1	0.0 ± 0.0	0.0 ± 0.0	0.0 ± 0.0	0.0 ± 0.0
***Methanosphaera* sp. ISO3-F5**	2.2 ± 0.4	4.9 ± 0.3	6.2 ± 2.0	7.3 ± 0.9	3.2 ± 0.5	9.5 ± 2.1	6.9 ± 2.9	9.3 ± 1.9
Methanocorpusculum sinense	0.0 ± 0.0	0.0 ± 0.0	0.0 ± 0.0	0.0 ± 0.0	0.0 ± 0.0	0.0 ± 0.0	0.0 ± 0.0	0.0 ± 0.0
Methanoculleus sp.	0.0 ± 0.0	0.0 ± 0.0	0.0 ± 0.0	0.0 ± 0.0	0.0 ± 0.0	0.0 ± 0.0	0.0 ± 0.0	0.0 ± 0.0
Methanomicrobium mobile	0.3 ± 0.2	0.5 ± 0.1	0.0 ± 0.0	0.3 ± 0.1	5.0 ± 2.4	0.1 ± 0.0	0.1 ± 0.1	0.4 ± 0.2
Methanosaeta concilii	0.0 ± 0.0	0.0 ± 0.0	0.0 ± 0.0	0.0 ± 0.0	0.0 ± 0.0	0.0 ± 0.0	0.0 ± 0.0	0.0 ± 0.0
Methanimicrococcus blatticola	0.4 ± 0.3	0.3 ± 0.1	0.0 ± 0.0	0.2 ± 0.1	0.2 ± 0.1	2.7 ± 0.8	1.6 ± 1.2	0.4 ± 0.2
Methanosarcina horonobensis	0.0 ± 0.0	0.0 ± 0.0	0.0 ± 0.0	0.0 ± 0.0	0.0 ± 0.0	0.0 ± 0.0	0.0 ± 0.0	0.0 ± 0.0
Methanosarcina mazei	0.0 ± 0.0	0.0 ± 0.0	0.0 ± 0.0	0.0 ± 0.0	0.0 ± 0.0	0.0 ± 0.0	0.0 ± 0.0	0.0 ± 0.0
***Mmc.*[a]; Group 10 sp.**	3.4 ± 0.5	3.3 ± 0.2	4.3 ± 1.2	1.6 ± 0.2	0.9 ± 0.2	3.1 ± 0.6	3.5 ± 2.0	5.1 ± 1.1

	buffalo	cattle	bison	sheep	goat	deer	giraffes	camelids
Mmc; *Candidatus* Methanomethylophilus alvus	0.0 ± 0.0	1.5 ± 0.4	0.0 ± 0.0	0.0 ± 0.0	0.0 ± 0.0	0.0 ± 0.0	0.0 ± 0.0	0.0 ± 0.0
Mmc; Group 11 sp. BRNA1	0.0 ± 0.0	0.0 ± 0.0	0.0 ± 0.0	0.0 ± 0.0	0.0 ± 0.0	0.0 ± 0.0	0.0 ± 0.0	0.0 ± 0.0
Mmc.; Group 11 sp. CRM1	0.1 ± 0.1	0.1 ± 0.0	0.1 ± 0.0	0.1 ± 0.0	0.1 ± 0.1	0.2 ± 0.2	0.5 ± 0.2	0.0 ± 0.0
Mmc.; Group 11 sp. ISO4-G11	0.1 ± 0.0	0.2 ± 0.0	0.1 ± 0.0	0.1 ± 0.0	0.1 ± 0.1	0.1 ± 0.0	0.4 ± 0.3	0.2 ± 0.1
Mmc.; Group 11 sp. from human dental plaque	0.0 ± 0.0	0.0 ± 0.0	0.0 ± 0.0	0.0 ± 0.0	0.0 ± 0.0	0.0 ± 0.0	0.0 ± 0.0	0.0 ± 0.0
***Mmc.*; Group 12 sp. ISO4-H5**	16.2 ± 2.6	7.8 ± 0.5	1.7 ± 0.7	3.0 ± 0.5	5.8 ± 1.5	3.3 ± 0.9	7.2 ± 2.3	4.5 ± 2.0
Mmc.; Group 3b sp.	0.2 ± 0.0	0.1 ± 0.0	0.0 ± 0.0	0.0 ± 0.0	0.0 ± 0.0	0.0 ± 0.0	0.4 ± 0.2	0.1 ± 0.0
Mmc.; Group 4 sp. MpT1	1.3 ± 0.2	1.2 ± 0.1	0.2 ± 0.1	0.7 ± 0.1	0.5 ± 0.2	0.7 ± 0.3	0.5 ± 0.3	0.6 ± 0.2
Mmc.; Group 8 sp.	0.1 ± 0.0	0.6 ± 0.1	0.2 ± 0.1	0.2 ± 0.1	0.3 ± 0.1	0.3 ± 0.1	0.1 ± 0.0	0.0 ± 0.0
Mmc.; Group 9 sp.	1.2 ± 0.3	0.7 ± 0.1	0.4 ± 0.1	1.2 ± 0.2	2.2 ± 0.7	1.5 ± 0.5	6.5 ± 2.1	4.8 ± 2.2

Supplementary Table 5

Metabolic classification of rumen methanogens.

Metabolism	Groups
Hydrogenotrophs[a]	*Methanobacterium* spp., *Methanobrevibacter* spp., *Methanocorpusculum* spp., *Methanoculleus* spp., *Methanomicrobium* spp.
Hydrogen dependent methylotrophs[b]	*Methanosphaera* spp., *Methanimicrococcus* spp., members of *Methanomassiliicoccaceae*
Aceticlastic methanogens[c]	*Methanosaeta* spp.
Generalists	*Methanosarcina* spp.
Unclassified metabolism	Other groups, No BLAST hits

[a]Form methane from hydrogen plus carbon dioxide, and some can use formate.

[b]Form methane from hydrogen plus methyl groups.

[c]Form methane from acetate.

Supplementary Table 6

The most abundant and prevalent rumen protozoal (Pr) genus-level groups.

Pr[a] ID	Group	Mean relative abundance (%, ±SEM[b]) All samples (n = 592)[c]			Adjusted for prevalence[d]			Maximum (%)[e]	Prevalence (%)[f]
1	*Anoplodinium-Diplodinium*	5.2	±	0.4	6.6	±	0.3	70.8	78.7
2	*Enoploplastron*	1.1	±	0.3	6.1	±	0.6	66.8	17.7
3	*Entodinium*	38.2	±	1.2	38.3	±	1.6	99.9	99.8
4	*Epidinium*	16.5	±	1.0	18.0	±	0.8	99.7	91.6
5	*Eremoplastron-Diploplastron*	4.4	±	0.3	5.8	±	0.3	74.5	75.5
6	*Eudiplodinium*	6.9	±	0.5	8.0	±	0.4	97.7	85.8
7	*Metadinium*	3.9	±	0.4	6.4	±	0.3	87.9	60.5
8	*Ophryoscolex*	2.0	±	0.3	5.4	±	0.4	59.6	37.5
9	*Ostracodinium*	6.8	±	0.5	9.0	±	0.4	73.4	75.3
10	*Polyplastron*	5.7	±	0.4	6.9	±	0.3	99.1	82.8
11	*Dasytricha*	3.4	±	0.2	4.2	±	0.2	45.9	79.4
12	*Isotricha*	6.0	±	0.5	6.9	±	0.3	99.4	86.7
13	No BLAST hit	0.0	±	0.0	0.1	±	0.1	0.3	0.5
14	Other groups[g]	0.0	±	0.0	0.0	±	0.0	0.0	45.4

[a]Protozoa, [b]standard error of the mean; [c]all samples for which at least 100 protozoal sequencing reads were available for analysis; [d]mean relative abundance in samples that contain that group; [e]the highest relative abundance observed in any one sample; [f]the prevalence of a group in all samples (n = 592); [g]relative abundance <0.5% in any one sample.

Supplementary Table 7
Diet classifications.

Criterion	Categories[a]	Examples
Forage to concentrate ratio	F	Forage- or browse-dominated (>70% forage or browse)
	FC	Mixed forage-concentrate (50-70% forage or browse)
	CF	Mixed concentrate-forage (50-70% concentrate)
	C	Concentrate-dominated (>70% concentrate)
Forage type (for diets with >30% forage)	Preserved whole crops (PWC)	Chaff, hay, haylage, silage
	Preserved non-seed residues of crops (PPC)	Straw, stover
	Fresh	Pasture (grass, clover, forage plantain, etc.) and browse
Forage plant (for diets with >30% forage)	Legume	Acacia, alfalfa, clover, leadtree
	Cereal	Maize, barley, rice, sorghum, wheat
	Tuber	Cassava
	Browse	Leaves, twigs, or other high-growing vegetation such as acacia (camelthorn), berry leaf, leadtree, matagouri, willow
	Grass	Ryegrass, sugarcane, and other tropical grasses

[a]Some diets contain components that fall into multiple categories. For example, acacia and leadtrees are both legumes and shrubs. Also, animals may be fed a mixture of forage plants and types.

Supplementary Table 8

Identities of the 50 most abundant and prevalent bacterial operational taxonomic units (OTUs[a]).

OTU	Abundance (%)	Prevalence (%)	Taxonomic assignment	Best BLAST match[b]				
				Material	Name	Strain	Accession	Similarity (%)
703605	0.12	63.01	Unclassified *Bacteroidales*	Cultured:	*Bacteroidetes* bacterium	G4	AB730781	100.0
				Type:	*Odoribacter splanchnicus*	1651/6	CP002544	81.9
454127	0.25	68.13	*Prevotella*	Cultured:	*Prevotella ruminicola*	Bryant 23	CP002006	100.0
				Type:	*Prevotella ruminicola*	Bryant 23	CP002006	100.0
664059	0.24	14.62	*Prevotella*	Cultured:	*Prevotella ruminicola*	Tc2-3	AF218617	99.4
				Type:	*Prevotella histicola*	T05-04	AB547685	90.7
762328	0.16	65.64	*Prevotella*	Cultured:	*Prevotella ruminicola*	BPI-34	AB501152	99.4
				Type:	*Prevotella ruminicola*	Bryant 23	CP002006	98.6
177612	0.13	56.29	*Prevotella*	Cultured:	*Prevotella* sp.	RM13	AB730677	99.2
				Type:	*Prevotella ruminicola*	Bryant 23	CP002006	93.2
142948	0.56	51.46	*Prevotella*	Cultured:	*Prevotella* aff. ruminicola	Tc2-24	AJ009933	95.3
				Type:	*Prevotella ruminicola*	Bryant 23	CP002006	91.5
4644	0.20	58.63	*Prevotella*	Cultured:	*Prevotella* sp.	BP1-145	AB501165	94.9
				Type:	*Prevotella ruminicola*	Bryant 23	CP002006	91.8
46566	0.14	55.41	*Prevotella*	Cultured:	Rumen bacterium	YS1	AF544206	94.5
				Type:	*Prevotella ruminicola*	Bryant 23	CP002006	89.4
576819	0.13	38.74	*Prevotella*	Cultured:	*Prevotella* aff. ruminicola	Tc2-24	AJ009933	94.1
				Type:	*Prevotella ruminicola*	Bryant 23	CP002006	93.0
369625	0.19	11.26	*Prevotella*	Cultured:	*Prevotella ruminicola*	Tf2-5	AF218620	93.3
				Type:	*Prevotella bryantii*	B14	AJ006457	89.6
158624	0.20	56.14	*Prevotella*	Cultured:	*Prevotella ruminicola*	CA61	AB849449	92.7
				Type:	*Prevotella ruminicola*	Bryant 23	CP002006	91.6
272296	0.16	18.27	*Prevotella*	Cultured:	*Prevotella* aff. ruminicola	Tc2-24	AJ009933	92.2
				Type:	*Prevotella brevis*	GA33	AJ011682	90.8
344518	0.13	37.13	*Prevotella*	Cultured:	*Prevotella ruminicola*	Tc2-28	AF218619	92.2
				Type:	*Prevotella ruminicola*	Bryant 23	CP002006	89.8
359129	0.12	34.94	*Prevotella*	Cultured:	Rumen bacterium	R-9	AB239482	91.6
				Type:	*Prevotella amnii*	JCM 14753	AB547670	89.2
362266	0.16	68.27	*Prevotella*	Cultured:	*Prevotella* sp.	R79	AB730668	91.3
				Type:	*Prevotella maculosa*	W1609	EF534315	88.2
758091	0.13	27.05	*Prevotella*	Cultured:	*Prevotella ruminicola*	Tc2-28	AF218619	89.5
				Type:	*Prevotella oulorum*	WPH 179	NR_029147	88.4
282075	0.22	42.25	*Prevotella*	Cultured:	*Prevotella* sp.	G57	AB730821	88.8
				Type:	*Prevotella shahii*	EHS11	AB108825	88.6
492371	0.10	61.70	*Prevotella*	Cultured:	*Prevotella ruminicola*	Tc2-28	AF218619	88.1
				Type:	*Prevotella loescheii*	Loesche 8B	AY836508	87.3
658563	0.13	59.06	RF16 (*Bacteroidales*)	Cultured:	Rumen bacterium	YS3	AF544208	93.8
				Type:	*Bacteroides coprophilus*	CB42	AB260026	82.4

OTU	Abundance (%)	Prevalence (%)	Taxonomic assignment	Best BLAST match[b]				
				Material	Name	Strain	Accession	Similarity (%)
480108	0.48	72.95	*Fibrobacter*	Cultured:	*Fibrobacter succinogenes*	R	AJ505938	100.0
				Type:	*Fibrobacter succinogenes*	S85	AJ496032	99.6
301049	0.15	64.04	*Fibrobacter*	Cultured:	*Fibrobacter succinogenes*	FGL 25	GU999989	99.4
				Type:	*Fibrobacter succinogenes*	S85	AJ496032	91.2
245302	0.23	70.03	*Fibrobacter*	Cultured:	*Fibrobacter succinogenes*	MC1	M62693	97.4
				Type:	*Fibrobacter succinogenes*	S85	AJ496032	90.4
97093	0.13	36.11	*Fibrobacter*	Cultured:	*Fibrobacter succinogenes*	AL225	AB275484	96.9
				Type:	*Fibrobacter succinogenes* subsp. *elongatus*	HM2	GU269553	93.1
25104	0.17	43.86	*Fibrobacter*	Cultured:	*Fibrobacter succinogenes*	FGL 25	GU999989	95.9
				Type:	*Fibrobacter succinogenes* subsp. *elongatus*	HM2	GU269553	93.3
439520	0.18	3.65	*Carnobacterium*	Cultured:	*Carnobacterium* sp.	LV62:W1	AF076637	100.0
				Type:	*Carnobacterium inhibens*	K1	Z73313	98.6
60575	0.14	6.43	*Lactobacillus*	Cultured:	Swine fecal bacterium	RF1A-Xy14	FJ753771	99.8
				Type:	*Lactobacillus mucosae*	S32	AB289204	99.8
451201	0.19	4.68	Unclassified *Leuconostocaceae*	Cultured:	*Weissella* sp.	H17	AB671284	99.8
				Type:	*Weissella hellenica*	LV346	X95981	99.8
178763	0.22	43.57	*Streptococcus*	Cultured:	*Streptococcus equinus*	C3	JQ837459	100.0
				Type:	*Streptococcus lutetiensis*	S182	DQ232532	99.8
248019	0.43	75.44	Unclassified *Clostridiales*	Cultured:	*Clostridiales* bacterium	R2	AB730625	99.8
				Type:	*Clostridium straminisolvens*	CSK1	AB125279	87.5
205298	0.08	65.35	Unclassified *Clostridiales*	Cultured:	*Lachnospiraceae* bacterium	RM29	AB730686	99.6
				Type:	*Eubacterium oxidoreducens*	G41	FR733672	91.6
662614	0.08	66.37	Unclassified *Clostridiales*	Cultured:	*Lachnospiraceae* bacterium	CA43	AB849443	98.9
				Type:	*Roseburia hominis*	A2-183	CP003040	90.6
206943	0.14	72.95	Unclassified *Clostridiales*	Cultured:	Rumen bacterium	R-7	AB239481	97.8
				Type:	*Clostridium clariflavum*	EBR45	AB186359	85.9
327162	0.27	78.22	Unclassified *Clostridiales*	Cultured:	*Clostridiales* bacterium	R47	AB730648	97.0
				Type:	*Clostridium clariflavum*	EBR45	AB186359	85.2
113096	0.06	58.63	Unclassified *Clostridiales*	Cultured:	*Clostridiales* bacterium	P37	AB730749	96.5
				Type:	*Clostridium clariflavum*	EBR45	AB186359	85.5
90393	0.50	82.89	Unclassified *Clostridiales*	Cultured:	*Clostridiales* bacterium	P37	AB730749	96.3
				Type:	*Clostridium clariflavum*	EBR45	AB186359	85.9
250686	0.07	61.26	Unclassified *Clostridiales*	Cultured:	*Clostridiales* bacterium	R47	AB730648	95.9
				Type:	*Clostrididum cellulolyuticum*	H10	CP001348	85.8
4854	0.05	63.01	Unclassified *Clostridiales*	Cultured:	*Clostridiales* bacterium	R47	AB730648	95.7
				Type:	*Clostridium cellobioparum*	Cc	NR_026104	88.5
330704	0.17	67.98	Unclassified *Clostridiales*	Cultured:	*Clostridiales* bacterium	R47	AB730648	95.1
				Type:	*Clostridium clariflavum*	EBR45	NR_102987	85.3
232168	0.05	61.55	Unclassified *Clostridiales*	Cultured:	*Clostridiales* bacterium	R47	AB730648	94.7
				Type:	*Clostridium cellobioparum*	Cc	NR_026104	88.5
137925	0.05	57.60	Unclassified *Clostridiales*	Cultured:	*Clostridiales* bacterium	P37	AB730749	93.7

OTU	Abundance (%)	Prevalence (%)	Taxonomic assignment	Best BLAST match[b]				
				Material	Name	Strain	Accession	Similarity (%)
				Type:	*Clostridium clariflavum*	EBR45	AB186359	85.7
311462	0.06	60.09	Unclassified *Clostridiales*	Cultured:	*Clostridium* sp.	DR6A	Y10028	92.5
				Type:	*Clostridium amygdalinum*	BR-10	AY353957	91.9
426876	0.12	61.55	Unclassified *Clostridiales*	Cultured:	*Clostridium straminisolvens*	CSK1	AB125279	85.4
				Type:	*Clostridium straminisolvens*	CSK1	AB125279	85.4
60316	0.07	62.72	*Mogibacterium*	Cultured:	Swine manure bacterium	37-2	AY167964	89.6
				Type:	*Mogibacterium neglectum*	P9a-h	AB037875	89.0
404437	0.04	57.89	*Mogibacterium*	Cultured:	Swine manure bacterium	37-2	AY167964	88.9
				Type:	*Mogibacterium vescum*	D5-2	AB021702	88.6
10546	0.11	65.06	Unclassified *Christensenellaceae*	Cultured:	*Christensenella minuta*	YIT 12065	AB490809	87.4
				Type:	*Christensenella minuta*	YIT 12065	AB490809	87.4
98494	0.15	59.80	Unclassified *Christensenellaceae*	Cultured:	*Clostridiales* bacterium	P37	AB730749	87.3
				Type:	*Christensenella minuta*	YIT 12065	AB490809	86.8
84178	0.06	58.04	*Clostridium*	Cultured:	Rumen bacterium	NK4A65	GU324373	96.2
				Type:	*Saccharofermentans acetigenes*	P6	AB910750	91.6
698124	0.26	64.04	Unclassified *Lachnospiraceae*	Cultured:	*Lachnospiraceae* bacterium	R62	AB730658	99.8
				Type:	*Clostridium hylemonae*	TN-271	AB910737	85.7
605934	0.07	58.48	Unclassified *Lachnospiraceae*	Cultured:	*Lachnospiraceae* bacterium	CG2	AB849413	93.8
				Type:	*Clostridium jejuense*	HY-35-12	AY494606	92.3
301314	0.05	58.92	*Butyrivibrio*	Cultured:	*Butyrivibrio hungatei*	AR10	FJ794074	100.0
				Type:	*Butyrivibrio hungatei*	JK615	AJ428553	99.4
164551	0.04	57.60	*Butyrivibrio*	Cultured:	*Butyrivibrio* sp.	G18	AB730792	98.0
				Type:	*Butyrivibrio proteoclasticus*	B316	CP001810	95.1
287012	0.25	54.53	*Butyrivibrio*	Cultured:	*Butyrivibrio sp.*	P79	AB730778	92.0
				Type:	*Butyrivibrio fibrisolvens*	D1	U41172	89.3
550791	0.16	57.89	*Butyrivibrio*	Cultured:	*Butyrivibrio* sp.	P79	AB730778	90.8
				Type:	*Clostridium clostridioforme*	Bryant T90	M59089	89.0
493059	0.35	80.85	*Butyrivibrio*	Cultured:	*Butyrivibrio* sp.	P79	AB730778	90.4
				Type:	*Clostrididum xylanivorans*	HESP1	AF116920	89.7
495334	0.13	63.01	*Butyrivibrio*	Cultured:	*Butyrivibrio* sp.	P79	AB730778	90.0
				Type:	*Clostrididum xylanivorans*	HESP1	AF116920	89.8
234051	0.07	64.33	*Coprococcus*	Cultured:	*Lachnospiraceae* bacterium	RM2	AB730670	99.8
				Type:	*Eubacterium ruminantium*	GA 195	AB008552	97.1
109054	0.05	59.65	*Moryella*	Cultured:	*Desulfotomaculum* sp.	CYP1	DQ479411	93.9
				Type:	*Desulfotomaculum guttoideum*	50	Y11568	93.5
237285	0.41	86.99	*Pseudobutyrivibrio*	Cultured:	*Butyrivibrio* sp.	3	EU714406	100.0
				Type:	*Pseudobutyrivibrio ruminis*	A12-1	X95893	98.5
139212	0.18	80.56	Unclassified *Ruminococcaceae*	Cultured:	Rumen bacterium	NK4A237	GU324386	100.0
				Type:	*Saccharofermentans acetigenes*	P6	AB910750	87.8
237588	0.07	57.89	Unclassified *Ruminococcaceae*	Cultured:	Rumen bacterium	NK3A39	GU324361	99.6
				Type:	*Ruminococcus bicirculans*	80/3	HF545616	90.4

OTU	Abundance (%)	Prevalence (%)	Taxonomic assignment	Best BLAST match[b]				
				Material	Name	Strain	Accession	Similarity (%)
494938	0.08	68.57	Unclassified *Ruminococcaceae*	Cultured:	Rumen bacterium	NK4A76	GU324387	99.4
				Type:	*Saccharofermentans acetigenes*	P6	AB910750	87.4
751129	0.08	67.40	Unclassified *Ruminococcaceae*	Cultured:	Rumen bacterium	NK4A214	GU324404	95.2
				Type:	*Oscillibacter ruminantium*	GH1	JF750939	88.8
577394	0.05	58.19	Unclassified *Ruminococcaceae*	Cultured:	Rumen bacterium	NK4A214	GU324404	94.8
				Type:	*Oscillibacter ruminantium*	GH1	JF750939	88.8
401207	0.12	77.05	Unclassified *Ruminococcaceae*	Cultured:	Rumen bacterium	NK4A214	GU324404	94.4
				Type:	*Oscillibacter ruminantium*	GH1	JF750939	88.0
732718	0.14	15.79	Unclassified *Ruminococcaceae*	Cultured:	*Blautia wexlerae*	WAL 14507	EF036467	93.9
				Type:	*Blautia wexlerae*	WAL 14507	EF036467	93.9
295461	0.15	50.15	Unclassified *Ruminococcaceae*	Cultured:	*Anaerotruncus colihominis*	WAL 14565	NR_027558	87.6
				Type:	*Anaerotruncus colihominis*	WAL 14565	NR_027558	87.6
580981	0.14	37.57	*Ruminococcus*	Cultured:	*Ruminococcaceae* bacterium	P7	AB730726	94.6
				Type:	*Ruminococcus bromii*	ATCC 27255	L76600	93.4
268113	0.11	64.77	*Succiniclasticum*	Cultured:	*Succiniclasticum ruminis*	SE10	X81137	99.8
				Type:	*Succiniclasticum ruminis*	SE10	X81137	99.8
284365	0.27	75.58	*Succiniclasticum*	Cultured:	*Succiniclasticum ruminis*	SE10	X81137	93.8
				Type:	*Succiniclasticum ruminis*	SE10	X81137	93.8
694360	0.06	64.91	RFN20 (*Erysipelotrichaceae*)	Cultured:	*Acholeplasma axanthum*	118	AJ311394	84.7
				Type:	*Acholeplasma parvum*	H23M	AY538170	84.6
410550	0.13	65.35	Unclassified *Victivallaceae*	Cultured:	*Cytophaga* sp.	PRPR22	DQ903989	88.3
				Type:	*Hydrogenispora ethanolica*	LX-B	AB669474	87.9
365725	0.24	17.54	Unclassified *Succinivibrionaceae*	Cultured:	*Succinivibrio dextrinosolvens*	0554	Y17600	96.7
				Type:	*Succinivibrio dextrinosolvens*	0554	Y17600	96.7
15480	0.26	34.06	Unclassified *Succinivibrionaceae*	Cultured:	*Succinivibrio dextrinosolvens*	CA76	AB849336	86.4
				Type:	*Succinivibrio dextrinosolvens*	0554	Y17600	86.2
722152	0.56	17.98	Unclassified *Succinivibrionaceae*	Cultured:	*Haemophilus sputorum*	HK 2154	JF506645	86.0
				Type:	*Haemophilus sputorum*	CCUG 13788	JF506642	86.0
9138	0.23	22.37	*Ruminobacter*	Cultured:	*Ruminobacter* sp.	RM87	AB730720	97.1
				Type:	*Ruminobacter amylophilus*	H-18	Y15992	96.9
571472	0.19	6.87	*Succinivibrio*	Cultured:	*Succinivibrio dextrinosolvens*	0554	Y17600	96.5
				Type:	*Succinivibrio dextrinosolvens*	0554	Y17600	96.5
60839	0.08	64.91	Unclassified (SR1)	Cultured:	*Sulfurospirillum alkalitolerans*	HTRB-L1	GQ863490	84.1
				Type:	*Sulfurospirillum alkalitolerans*	HTRB-L1	GQ863490	84.1

[a]The 77 most abundant and prevalent OTUs represented 12.9% of all sequence data (588,053 of 4,557,252 sequencing reads for 684 samples). These were the unique OTUs from the 50 most abundant (i. e., mean number of reads per samples) and 50 most prevalent (i. e., the number of samples in which the OTU was detected). [b]Matches and similarities were calculated using megablast against the nt database on 4 November 2014, minimum sequence alignment was 250 bp.

Supplementary Table 9

Identities of the 50 most abundant and prevalent archaeal operational taxonomic units (OTUs[a]).

OTU	Abundance (%)	Prevalence (%)	Taxonomic assignment	Best BLAST match [b]				
				Material	Name	Strain	Accession	Similarity (%)
23313	0.24	35.94	*Methanobacterium* sp.	Cultured:	*Methanobacterium flexile*	GH	NR_116276	98.45
				Type:	*Methanobacterium flexile*	GH	NR_116276	98.45
7020	0.97	4.69	*Methanobrevibacter boviskoreani*	Cultured:	*Methanobrevibacter* sp.	AbM4	AJ550156	100
				Type:	*Methanobrevibacter boviskoreani*	JH1	NR_118565	100
1042	0.22	69.60	*Methanobrevibacter gottschalkii* clade	Cultured:	*Methanobrevibacter* sp.	110	EF112194	100
				Type:	*Methanobrevibacter gottschalkii*	HO	NR_044789	98.68
5935	23.83	100.00	*Methanobrevibacter gottschalkii* clade	Cultured:	*Methanobrevibacter* sp.	1Y	DQ135988	99.78
				Type:	*Methanobrevibacter gottschalkii*	HO	NR_044789	98.9
7973	1.91	87.07	*Methanobrevibacter gottschalkii* clade	Cultured:	*Methanobrevibacter* sp.	SM9	AJ009958	99.56
				Type:	*Methanobrevibacter millerae*	ZA-10	NR_042785	98.68
30520	2.30	77.56	*Methanobrevibacter gottschalkii* clade	Cultured:	*Methanogenic archaeon*	LGM-ZA4	KF649310	99.53
				Type:	*Methanobrevibacter gottschalkii*	HO	NR_044789	98.25
12472	3.14	84.23	*Methanobrevibacter gottschalkii* clade	Cultured:	*Methanobrevibacter* sp.	SM9	AJ009958	99.34
				Type:	*Methanobrevibacter millerae*	ZA-10	NR_042785	98.02
23113	0.59	85.94	*Methanobrevibacter gottschalkii* clade	Cultured:	*Methanobrevibacter* sp.	1Y	DQ135988	99.34
				Type:	*Methanobrevibacter gottschalkii*	HO	NR_044789	98.47
23186	0.22	54.97	*Methanobrevibacter gottschalkii* clade	Cultured:	*Methanobrevibacter* sp.	1Y	DQ135988	99.34
				Type:	*Methanobrevibacter gottschalkii*	HO	NR_044789	98.47
7383	0.11	52.98	*Methanobrevibacter gottschalkii* clade	Cultured:	*Methanobrevibacter* sp.	110	EF112194	99.33
				Type:	*Methanobrevibacter gottschalkii*	HO	NR_044789	97.46
31163	0.14	45.74	*Methanobrevibacter gottschalkii* clade	Cultured:	*Methanobrevibacter* sp.	110	EF112194	99.33
				Type:	*Methanobrevibacter gottschalkii*	HO	NR_044789	98.03
8158	0.47	58.81	*Methanobrevibacter gottschalkii* clade	Cultured:	*Methanobrevibacter* sp.	1Y	DQ135988	99.13
				Type:	*Methanobrevibacter gottschalkii*	HO	NR_044789	98.27
980	0.39	69.18	*Methanobrevibacter gottschalkii* clade	Cultured:	*Methanobrevibacter* sp.	1Y	DQ135988	99.12
				Type:	*Methanobrevibacter gottschalkii*	HO	NR_044789	98.25
23622	0.15	55.97	*Methanobrevibacter gottschalkii* clade	Cultured:	*Methanobrevibacter* sp.	1Y	DQ135988	99.12
				Type:	*Methanobrevibacter gottschalkii*	HO	NR_044789	98.25
24768	0.69	65.34	*Methanobrevibacter gottschalkii* clade	Cultured:	*Methanobrevibacter* sp.	1Y	DQ135988	99.12
				Type:	*Methanobrevibacter gottschalkii*	HO	NR_044789	98.25
16049	0.21	60.94	*Methanobrevibacter gottschalkii* clade	Cultured:	*Methanobrevibacter* sp.	110	EF112194	99.1
				Type:	*Methanobrevibacter gottschalkii*	HO	NR_044789	97.81
3866	0.10	48.15	*Methanobrevibacter gottschalkii* clade	Cultured:	*Methanobrevibacter* sp.	1Y	DQ135988	98.9
				Type:	*Methanobrevibacter gottschalkii*	HO	NR_044789	98.03
14179	0.25	32.67	*Methanobrevibacter gottschalkii* clade	Cultured:	*Methanobrevibacter* sp.	SM9	AJ009958	98.9
				Type:	*Methanobrevibacter millerae*	ZA-10	NR_042785	98.02
29352	0.14	41.90	*Methanobrevibacter gottschalkii* clade	Cultured:	*Methanobrevibacter* sp.	1Y	DQ135988	98.9
				Type:	*Methanobrevibacter gottschalkii*	HO	NR_044789	98.03

OTU	Abundance (%)	Prevalence (%)	Taxonomic assignment	Best BLAST match [b]				
				Material	Name	Strain	Accession	Similarity (%)
2221	0.17	66.34	*Methanobrevibacter gottschalkii* clade	Cultured:	*Methanobrevibacter* sp.	1Y	DQ135988	98.68
				Type:	*Methanobrevibacter smithii*	PS	CP000678	98.02
14816	0.32	70.74	*Methanobrevibacter gottschalkii* clade	Cultured:	*Methanobrevibacter* sp.	1Y	DQ135988	98.46
				Type:	*Methanobrevibacter gottschalkii*	HO	NR_044789	97.59
24688	0.91	89.06	*Methanobrevibacter gottschalkii* clade	Cultured:	*Methanobrevibacter* sp.	1Y	DQ135988	98.24
				Type:	*Methanobrevibacter gottschalkii*	HO	NR_044789	97.37
3477	0.12	42.61	*Methanobrevibacter gottschalkii* clade	Cultured:	*Methanogenic archaeon*	LGM-ZA4	KF649310	97.87
				Type:	*Methanobrevibacter gottschalkii*	HO	NR_044789	96.71
28940	0.13	53.98	*Methanobrevibacter gottschalkii* clade	Cultured:	*Methanobrevibacter* sp.	1Y	DQ135988	96.48
				Type:	*Methanobrevibacter gottschalkii*	HO	NR_044789	95.61
4830	10.98	94.60	*Methanobrevibacter ruminantium* clade	Cultured:	*Methanobrevibacter* sp.	AK-87	AY615202	100
				Type:	*Methanobrevibacter ruminantium*	M1	CP001719	99.12
30008	0.21	51.56	*Methanobrevibacter ruminantium* clade	Cultured:	*Methanobrevibacter* sp.	M1	CP001719	100
				Type:	*Methanobrevibacter ruminantium*	M1	CP001719	100
23690	0.41	62.22	*Methanobrevibacter ruminantium* clade	Cultured:	*Methanobrevibacter* sp.	NT7	AJ009959	99.78
				Type:	*Methanobrevibacter ruminantium*	M1	CP001719	98.89
4213	0.17	56.53	*Methanobrevibacter ruminantium* clade	Cultured:	*Methanobrevibacter* sp.	AK-87	AY615202	99.56
				Type:	*Methanobrevibacter ruminantium*	M1	CP001719	98.68
24771	0.35	34.80	*Methanobrevibacter ruminantium* clade	Cultured:	*Methanobrevibacter* sp.	Z4	AY196670	99.56
				Type:	*Methanobrevibacter ruminantium*	M1	CP001719	98.69
5808	0.18	57.81	*Methanobrevibacter ruminantium* clade	Cultured:	*Methanobrevibacter* sp.	Z4	AY196670	99.34
				Type:	*Methanobrevibacter ruminantium*	M1	CP001719	98.45
18930	2.32	70.45	*Methanobrevibacter ruminantium* clade	Cultured:	*Methanobrevibacter* sp.	Z4	AY196670	99.12
				Type:	*Methanobrevibacter ruminantium*	M1	CP001719	98.89
19640	0.12	46.88	*Methanobrevibacter ruminantium* clade	Cultured:	*Methanobrevibacter* sp.	Z4	AY196670	99.12
				Type:	*Methanobrevibacter ruminantium*	M1	CP001719	98.01
2022	0.08	42.47	*Methanobrevibacter ruminantium* clade	Cultured:	*Methanobrevibacter* sp.	Z4	AY196670	98.89
				Type:	*Methanobrevibacter ruminantium*	M1	CP001719	97.79
30272	0.11	50.57	*Methanobrevibacter ruminantium* clade	Cultured:	*Methanobrevibacter* sp.	NT7	AJ009959	98.89
				Type:	*Methanobrevibacter ruminantium*	M1	CP001719	98.89
10213	3.62	56.25	*Methanobrevibacter ruminantium* clade	Cultured:	*Methanobrevibacter* sp.	Z4	AY196670	98.45
				Type:	*Methanobrevibacter olleyae*	KM1H5-1P	NR_043024	98.01
24733	0.73	76.14	*Methanobrevibacter ruminantium* clade	Cultured:	*Methanobrevibacter* sp.	Z4	AY196670	98.45
				Type:	*Methanobrevibacter ruminantium*	M1	CP001719	97.35
12140	0.12	50.28	*Methanobrevibacter ruminantium* clade	Cultured:	*Methanobrevibacter* sp.	AK-87	AY615202	98.01
				Type:	*Methanobrevibacter ruminantium*	M1	CP001719	97.12
15117	0.26	55.97	*Methanobrevibacter ruminantium* clade	Cultured:	*Methanobrevibacter* sp.	NT7	AJ009959	97.57
				Type:	*Methanobrevibacter ruminantium*	M1	CP001719	97.57
21114	0.10	42.19	*Methanobrevibacter ruminantium* clade	Cultured:	*Methanobrevibacter* sp.	SM9	AJ009958	97.12
				Type:	*Methanobrevibacter millerae*	ZA-10	NR_042785	96.26
11748	0.19	46.59	*Methanobrevibacter ruminantium* clade	Cultured:	*Methanobrevibacter* sp.	NT7	AJ009959	96.9

OTU	Abundance (%)	Prevalence (%)	Taxonomic assignment	Best BLAST match [b]				
				Material	Name	Strain	Accession	Similarity (%)
				Type:	*Methanobrevibacter ruminantium*	M1	CP001719	96.46
26071	0.59	77.27	*Methanobrevibacter ruminantium* clade	Cultured:	*Methanobrevibacter* sp.	NT7	AJ009959	96.68
				Type:	*Methanobrevibacter olleyae*	KM1H5-1P	NR_043024	96.46
23917	0.28	69.18	*Methanobrevibacter ruminantium* clade	Cultured:	*Methanobrevibacter smithii*	ALI-A	AY196667	96.48
				Type:	*Methanobrevibacter smithii*	PS	CP000678	96.48
3174	0.10	42.90	*Methanobrevibacter ruminantium* clade	Cultured:	*Methanobrevibacter* sp.	AK-87	AY615202	96.02
				Type:	*Methanosphaera stadtmanae*	MCB-3	CP000102	95.35
1149	0.62	28.84	*Methanobrevibacter wolinii*	Cultured:	*Methanobrevibacter* sp	AbM4	AJ550156	94.74
				Type:	*Methanobrevibacter boviskoreani*	JH1	NR_118565	94.74
17220	3.24	94.03	*Methanosphaera* sp. ISO3-F5	Cultured:	*Methanosphaera stadtmanae*	MCB-3	CP000102	95.81
				Type:	*Methanosphaera stadtmanae*	MCB-3	CP000102	95.81
13735	0.27	65.06	*Methanosphaera* sp. ISO3-F5	Cultured:	*Methanosphaera stadtmanae*	MCB-3	CP000102	94.26
				Type:	*Methanosphaera stadtmanae*	MCB-3	CP000102	94.26
8203	1.37	55.54	*Methanosphaera* sp. Group 5	Cultured:	*Methanosphaera cuniculi*	1R7	NR_104874	96.47
				Type:	*Methanosphaera cuniculi*	1R7	NR_104874	96.47
18417	0.68	17.90	*Methanomicrobium mobile*	Cultured:	*Methanomicrobium mobile*	BP	NR_044726	100
				Type:	*Methanomicrobium mobile*	BP	NR_044726	100
1562	0.17	15.48	*Methanimicrococcus blatticola*	Cultured:	*Methanimicrococcus blatticola*	PA	NR_115170	94.24
				Type:	*Methanimicrococcus blatticola*	PA	NR_115170	94.24
21128	0.22	6.11	*Methanimicrococcus blatticola*	Cultured:	*Methanimicrococcus blatticola*	PY-22	JQ268014	94.91
				Type:	*Methanimicrococcus blatticola*	PA	NR_115170	93.14
10999	0.46	54.40	*Mmc.*[c]*;* Group 10 sp.	Cultured:	Archaeon	PY-11	JQ268005	96.01
				Type:	*Methanomassiliicoccus luminyensis*	B10	NR_118098	88.74
19443	0.46	42.61	*Mmc;* Group 10 sp.	Cultured:	Methanogenic archaeon	CH1270	DQ445723	95.49
				Type:	*Methanomassiliicoccus luminyensis*	B10	NR_118098	88.52
21341	0.90	60.23	*Mmc;* Group 10 sp.	Cultured:	Methanogenic archaeon	WGK1	GQ339877	95.11
				Type:	*Methanomassiliicoccus luminyensis*	B10	NR_118098	88.94
22286	1.08	9.09	*Mmc; Candidatus* Methanomethylophilus alvus	Cultured:	*Candidatus* Methanomethylophilus alvus	Mx1201	CP004049	98.89
				Type:	*Methanomassiliicoccus luminyensis*	B10	NR_118098	84.55
15334	0.27	0.43	*Mmc; Candidatus* Methanomethylophilus alvus	Cultured:	*Candidatus* Methanomethylophilus alvus	Mx1201	CP004049	97.81
				Type:	*Methanomassiliicoccus luminyensis*	B10	NR_118098	84.35
9844	0.18	8.95	*Mmc;* Group 11 sp. CRM1	Cultured:	*Thermoplasmatales* archaeon	BRNA1	CP002916	96.89
				Type:	*Thermoplasma acidophilum*	122-1B2	AL445067	80.57
16960	0.22	40.77	*Mmc;* Group 11 sp. ISO4-G11	Cultured:	*Thermoplasmatales* archaeon	BRNA1	CP002916	97.11
				Type:	*Thermoplasma acidophilum*	122-1B2	AL445067	81.06
25835	1.76	56.39	*Mmc;* Group 12 sp. ISO4-H5	Cultured:	*Candidatus* Methanomethylophilus alvus	Mx1201	CP004049	97.12
				Type:	*Methanomassiliicoccus luminyensis*	B10	NR_118098	84.99
28326	3.21	63.35	*Mmc;* Group 12 sp. ISO4-H5	Cultured:	*Candidatus* Methanomethylophilus alvus	Mx1201	CP004049	96.02
				Type:	*Methanomassiliicoccus luminyensis*	B10	NR_118098	85.21
30594	0.90	63.21	*Mmc;* Group 12 sp. ISO4-H5	Cultured:	Archaeon	PY-11	JQ268005	95.14
				Type:	*Methanomassiliicoccus luminyensis*	B10	NR_118098	87.61

OTU	Abundance (%)	Prevalence (%)	Taxonomic assignment	Best BLAST match [b]				
				Material	Name	Strain	Accession	Similarity (%)
25646	0.23	41.19	*Mmc;* Group 4 sp. MpT1	Cultured:	Archaeon	PY-11	JQ268005	96.67
				Type:	*Methanomassiliicoccus luminyensis*	B10	NR_118098	89.18
25606	0.61	58.81	*Mmc;* Group 4 sp. MpT1	Cultured:	Archaeon	PY-11	JQ268005	96.24
				Type:	*Methanomassiliicoccus luminyensis*	B10	NR_118098	89.16
7359	0.23	34.38	*Mmc;* Group 8 sp.	Cultured:	Methanogenic archaeon	WGK1	GQ339877	97.11
				Type:	*Methanomassiliicoccus luminyensis*	B10	NR_118098	88.27
10221	0.48	51.42	*Mmc;* Group 9 sp.	Cultured:	Methanogenic archaeon	DCM1	GQ339876	96.01
				Type:	*Methanomassiliicoccus luminyensis*	B10	NR_118098	85.68

[a]The 64 most abundant and prevalent OTUs represented 76.2% of all sequence data (696,612 of 914,807 sequencing reads for 704 samples). These were the unique OTUs from the 50 most abundant (i. e., mean number of reads per samples) and 50 most prevalent (i. e., the number of samples in which the OTU was detected).[b]Matches and similarities were calculated using megablast against the nt database on 19 November 2014, minimum sequence alignment was 420 bp. [c]*Mmc*, *Methanomassiliicoccaceae*.

Supplementary Table 10
Bacterial groups considered to be exogenous to the rumen.

Group	Mean relative abundance (%, ±SEM[a]) All samples (n = 684)[b]	Maximum (%)[c]	Prevalence (%)[d]
Unclassified *Actinomycetaceae*	0.0 ± 0.0	0.7	11.5
Brevibacterium	0.1 ± 0.1	40.3	7.2
Dietzia	0.0 ± 0.0	0.9	10.0
Leucobacter	0.0 ± 0.0	0.9	5.0
Arthrobacter	0.1 ± 0.1	30.7	11.7
Nesterenkonia	0.0 ± 0.0	0.6	3.1
Rhodococcus	0.0 ± 0.0	2.9	18.2
Unclassified *Nocardiopsaceae*	0.0 ± 0.0	11.7	12.1
Nocardiopsis	0.0 ± 0.0	0.5	1.1
Sanguibacter	0.0 ± 0.0	1.7	3.3
Streptomyces	0.0 ± 0.0	0.5	5.8
Wautersiella	0.0 ± 0.0	0.6	2.6
Unclassified *Flavobacteriaceae*	0.0 ± 0.0	10.5	2.6
Sphingobacterium	0.0 ± 0.0	0.7	4.3
Unclassified *Streptophyta*	0.2 ± 0.0	15.2	44.6
Unclassified *Bacillales*	0.1 ± 0.0	24.0	5.7
Exiguobacterium	0.0 ± 0.0	10.6	5.7
Unclassified *Bacillaceae*	0.0 ± 0.0	7.6	13.3
Bacillus	0.1 ± 0.0	9.8	34.6
Unclassified *Listeriaceae*	0.0 ± 0.0	11.6	0.8
Brochothrix	0.0 ± 0.0	0.6	0.3
Unclassified *Paenibacillaceae*	0.0 ± 0.0	0.5	4.9
Brevibacillus	0.0 ± 0.0	5.7	5.6
Paenibacillus	0.0 ± 0.0	8.6	14.8
Unclassified *Planococcaceae*	0.0 ± 0.0	13.7	14.2
Kurthia	0.0 ± 0.0	20.3	2.0
Lysinibacillus	0.2 ± 0.0	14.6	9.5
Planococcus	0.3 ± 0.2	91.3	4.7
Planomicrobium	0.0 ± 0.0	2.7	3.8
Rummeliibacillus	0.1 ± 0.0	24.1	11.7
Solibacillus	0.4 ± 0.1	44.1	13.7
Sporosarcina	0.0 ± 0.0	1.3	3.8
Ureibacillus	0.0 ± 0.0	0.9	4.2
Jeotgalicoccus	0.0 ± 0.0	0.7	6.2
Macrococcus	0.0 ± 0.0	0.9	1.2
Salinicoccus	0.0 ± 0.0	2.7	3.5
Staphylococcus	0.1 ± 0.0	26.8	16.3
Laceyella	0.0 ± 0.0	0.6	3.1
Planifilum	0.0 ± 0.0	0.5	2.8
Thermoactinomyces	0.0 ± 0.0	1.5	8.3
Alkalibacterium	0.0 ± 0.0	6.6	3.8
Facklamia	0.0 ± 0.0	0.7	9.1
Alkaliphilus	0.1 ± 0.0	23.6	5.6

Group	Mean relative abundance (%, ±SEM[a]) All samples (n = 684)[b]	Maximum (%)[c]	Prevalence (%)[d]
Brevundimonas	0.0 ± 0.0	1.6	9.5
Unclassified *Rhizobiales*	0.0 ± 0.0	2.9	10.6
Ochrobactrum	0.0 ± 0.0	0.7	3.1
Agrobacterium	0.0 ± 0.0	0.7	18.9
Unclassified *Rhodobacteraceae*	0.0 ± 0.0	0.8	4.9
Rhodobacter	0.2 ± 0.1	30.1	6.8
Acetobacter	0.0 ± 0.0	0.5	8.3
Mitochondria	0.0 ± 0.0	1.7	15.5
Unclassified *Erythrobacteraceae*	0.0 ± 0.0	0.5	6.8
Unclassified *Sphingomonadaceae*	0.0 ± 0.0	0.8	5.2
Unclassified *Comamonadaceae*	0.0 ± 0.0	0.7	10.0
Comamonas	0.2 ± 0.1	28.0	11.8
Delftia	0.0 ± 0.0	1.2	2.7
Variovorax	0.0 ± 0.0	3.5	3.8
Unclassified *Aeromonadaceae*	0.0 ± 0.0	2.6	2.4
Shewanella	0.0 ± 0.0	4.4	2.8
Bibersteinia	0.0 ± 0.0	2.1	2.7
Acinetobacter	0.2 ± 0.0	28.0	16.8
Moraxella	0.0 ± 0.0	10.4	3.0
Psychrobacter	0.0 ± 0.0	5.5	2.8
Unclassified *Pseudomonadaceae*	0.0 ± 0.0	0.7	7.2
Pseudomonas	0.2 ± 0.1	35.2	19.7
Stenotrophomonas	0.1 ± 0.0	30.2	11.0

[a]Standard error of the mean; [b]all samples for which at least 500 bacterial sequencing reads were available for analysis; [c]the highest relative abundance observed in any one sample; [d]the prevalence of a group in all samples (n = 684).
